# Comparison of Joint Mobilization and Movement Pattern Training for Patients With Hip-Related Groin Pain: A Pilot Randomized Clinical Trial

**DOI:** 10.1093/ptj/pzad111

**Published:** 2023-08-22

**Authors:** Marcie Harris-Hayes, Patricia Zorn, Karen Steger-May, Megan M Burgess, Rebecca D DeMargel, Suzanne Kuebler, John Clohisy, Simon Haroutounian

**Affiliations:** Program in Physical Therapy, Department of Orthopaedic Surgery, Washington University School of Medicine, St. Louis, Missouri, USA; Patricia Zorn Center for Physical Therapy and Spine Rehabilitation, St. Louis, Missouri, USA; Center for Biostatistics and Data Science, Washington University School of Medicine, St. Louis, Missouri, USA; Program in Physical Therapy, Department of Orthopaedic Surgery, Washington University School of Medicine, St. Louis, Missouri, USA; Program in Physical Therapy, Washington University School of Medicine, St. Louis, Missouri, USA; Program in Physical Therapy, Washington University School of Medicine, St. Louis, Missouri, USA; Department of Orthopaedic Surgery, Washington University School of Medicine, St. Louis, Missouri, USA; Department of Anesthesiology and Washington University Pain Center, Washington University School of Medicine, St. Louis, Missouri, USA

**Keywords:** Femoroacetabular Impingement, Hip Dysplasia, Joint Mobilization, Kinematics, Movement System, Musculoskeletal, Physical Therapy, Physiotherapy, Rehabilitation

## Abstract

**Objective:**

The objective of this study was to assess the feasibility of completing a randomized clinical trial (RCT) and examine the preliminary effects of 2 interventions for hip-related groin pain (HRGP).

**Methods:**

In this pilot RCT, patients with HRGP, who were 18 to 40 years old, were randomized (1:1 ratio) to a joint mobilization (JtMob) group or a movement pattern training (MoveTrain) group. Both treatments included 10 supervised sessions and a home exercise program. The goal of JtMob was to reduce pain and improve mobility through peripherally and centrally mediated pain mechanisms. The key element was physical therapist-provided JtMob. The goal of MoveTrain was to reduce hip joint stresses by optimizing the biomechanics of patient-specific tasks. The key element was task-specific instruction to correct abnormal movement patterns displayed during tasks. Primary outcomes were related to future trial feasibility. The primary effectiveness outcome was the Hip Disability and Osteoarthritis Outcome Score. Examiners were blinded to group; patients and treatment providers were not. Data collected at baseline and immediately after treatment were analyzed with analysis of covariance using a generalized linear model in which change was the dependent variable and baseline was the covariate. The study was modified due to the coronavirus disease 2019 (COVID-19) pandemic.

**Results:**

The COVID-19 pandemic affected participation; 127 patients were screened, 33 were randomized (18 to the JtMob group and 15 to the MoveTrain group), and 29 (88%) provided posttreatment data. Treatment session adherence was 85%, and home exercise program component adherence ranged from 71 to 86%. Both groups demonstrated significant mean within-group improvements of ≥5 points on Hip Disability and Osteoarthritis Outcome Score scales. There were no between-group differences in effectiveness outcomes.

**Conclusions:**

A large RCT to assess the effects of JtMob and MoveTrain for patients with HRGP may be feasible. Preliminary findings suggested that JtMob or MoveTrain may result in improvements in patient-reported pain and activity limitations.

**Impact:**

The COVID-19 pandemic interfered with participation, but a randomized controlled trial may be feasible. Modification may be needed if the trial is completed during future pandemics.

## Introduction

Hip-related groin pain (HRGP),^1^ also referred to as hip-related pain,^2^ is often associated with femoroacetabular impingement, developmental dysplasia of the hip, and soft tissue injury, such as labral tears and chondral lesions; however, may also present with no imaging findings.^1^^,^^2^ Rehabilitation to address modifiable factors associated with HRGP may lead to improved function and reduced pain in people with HRGP, yet little is known about its effectiveness. Consensus statements^3^^,^^4^ recommend rehabilitation for patients with HRGP prior to considering surgery; however, the available evidence provides little guidance on which components to include in such intervention.^3^^,^^5^ Current studies assessing rehabilitation report small samples^6–9^ and often include a nonstandardized, multimodal approach,^8–11^ thus limiting translation to clinical practice. A clear need exists to rigorously assess interventions for HRGP to better understand the specific effect of each approach, potentially leading to better treatment personalization. In this paper, we report the results of a pilot randomized clinical trial (RCT) to assess the feasibility of completing a large RCT comparing the effects of 2 rehabilitation interventions, joint mobilization (JtMob), and movement pattern training (MoveTrain).

Multiple mechanisms underlying the therapeutic effect of manual therapy have been proposed.^12^^,^^13^ For this study using JtMob, we focused on the theoretical concept that mechanical forces provided during mobilization techniques result in a neurophysiological response that leads to reduction of pain perception and thus improved overall mobility.^14^ Mechanisms that lead to increased responsiveness of peripheral nociceptors (ie, peripheral sensitization) as well as within the central nervous system (ie, central sensitization) may contribute to pain persistence long after initial tissue injury.^15–17^ In their perspective related to management of pain processing mechanisms, Chimenti et al^18^ highlighted the potential effects of JtMob on peripherally and centrally mediated pain mechanisms. Peripherally, JtMob may reduce nociceptive pain by activating endocannabinoid systems in the periphery,^19^ reducing inflammation and promoting tissue healing through mediators such as resolvins,^20^^,^^21^ and possibly restoring joint motion that may remove the mechanical irritation to local nociceptors.^22^ Centrally, JtMob may produce analgesia by activating descending inhibitory pathways^23^ and reducing central nervous system excitability.^24–26^ Although JtMob has been shown to be effective in reducing pain in patients with hip and knee osteoarthritis,^27–29^ little has been reported in HRGP.

The theoretical concept underlying MoveTrain is that a person’s movement pattern may contribute to the development and persistence of pain related to HRGP. The Physical Stress Theory^30^ states that repetitive or prolonged stresses may result in tissue injury if the capacity of the tissue to accept stress is exceeded. The Movement System Impairment Theory^31^ builds upon the Physical Stress Theory by stating that mechanical hip pain may be the consequence of repeated use of abnormal movement patterns during daily activities. Abnormal movement patterns, such as excessive hip adduction, may create altered mechanical forces on joint structures, thus changing the location and magnitude of stress to specific joint tissues, such as the articular cartilage and acetabular labrum.^31^ Repeated loading of the hip joint with altered mechanical forces may contribute to cumulative tissue stress, micro-trauma, and pain.^32^ Until the abnormal movement pattern is modified, the hip pain may persist or recur. The key element of MoveTrain is task-specific instruction to correct abnormal movement patterns during a patient’s activities.

Our primary purpose was to determine the feasibility of completing a future RCT to determine the efficacy of JtMob compared to MoveTrain for people with HRGP. Our primary outcome measures included patient recruitment, retention and treatment adherence, and treatment fidelity. Our secondary purpose was to compare the preliminary effects of JtMob and MoveTrain on patient-reported outcomes measures, movement patterns, and pain pressure thresholds.

## Methods

### Study Design

We completed a 12-week parallel-group pilot RCT^33^^,^^34^ using a 1:1 allocation ratio. This study was approved by the Human Research Protection Offices of the Washington University School of Medicine and follows the ethics principles of the Helsinki Declaration.^35^ All patients signed an informed consent statement prior to participating. We wrote this report in accordance with Consolidated Standards of Reporting Trials (CONSORT) guidelines.^36^ The trial is registered at Clinicaltrials.gov (#NCT03959319).

### Modifications After Trial Commencement

On March 16, 2020, we suspended on-site activities, including enrollment, assessment, and treatment, due to the COVID**-**19 pandemic. Once the study resumed (November 11, 2020), we modified methods to reduce risks associated with COVID-19. We screened potential patients for risk of serious COVID-19 complications and only enrolled those with low risk. We screened patients and personnel for COVID-19 symptoms and exposure prior to each visit. All were required to wear personal protective equipment. We reduced the number of people in the assessment or treatment room to accommodate physical distancing recommendations. To decrease assessment time, we performed only those tests specific to study aims. Unrelated to the COVID-19 pandemic, we added Ehlers-Danlos syndrome as an exclusion criterion in May 2021.

### Manual of Operations Development and Training

Prior to study start, we developed a manual of operations documenting standard methods for all study components. To optimize treatment fidelity, we used our established training and assessment methods^37^ based on the treatment fidelity framework developed by the NIH Behavioral Change Consortium.^38^^,^^39^ Training was provided by the first (20 years of experience) and second (32 years of experience, advanced training in manual therapy) authors. Four physical therapists, with 9 to 18 years of clinical experience, participated in training and provided treatment (2 JtMob, 2 MoveTrain). Training included reading of the manual of operations and participation in on-site sessions with protocol developers that included review and discussion of overall study design and methodology, theoretical background of each treatment, assessment and treatment guidelines, and documentation methods. Case scenarios were used to demonstrate assessment and treatment concepts and to practice treatment implementation. Specific to JtMob, hands-on practice of mobilization techniques were used to standardize techniques among physical therapists. During training sessions, physical therapists were encouraged to provide feedback for methods proposed, and protocol modifications were made accordingly. Initial training time was approximately 20 hours, including 10 hours of on-site training and communications via webcast, phone calls, and emails.

### Participants

Table 1 provides inclusion and exclusion criteria. Potential patients from the community self-identified by viewing flyers posted in the community, advertisements, and social media (eg, Facebook, Twitter). Clinicians in Washington University Orthopaedic and Physical Therapy clinics identified potential patients based on study criteria and provided the patients with study and contact information. Interested patients contacted the research coordinator, who described the study, completed initial eligibility screening, and scheduled the baseline assessment for those qualified based on the screening. Informed written consent was obtained, and final eligibility was determined at baseline assessment prior to data collection. Data collection and treatment occurred in the Movement Science Research Center at the Washington University Program in Physical Therapy.

**Table 1:** Inclusion and Exclusion Criteria

**Time When Information Was Obtained**	**Inclusion Criteria**	**Exclusion Criteria**
Initial screening	Young and middle-aged adults,^2^^,^^78^ 18–40 years old*^a^*	Previous hip surgery, fracture, pelvic/hip infection, avascular necrosis, or cancer
	Report of frequent hip joint or groin symptoms, defined as pain, aching, or stiffness within the hip joint for at least 3 mo during the past 12 mo^79–81^	Pain due to high-impact trauma
	Report of usual pain^82^ of ≥3/10^46^^,^*^b^*	Inflammatory disease, such as rheumatoid arthritis or gout
	Report of functional limitation, as demonstrated by modified Harris Hip Score of <90^46^	Acute pain in another joint that limits functional activities
		Perthes disease or slipped capital femoral epiphysis
		Neurological involvement affecting balance or coordination
		Use of assistive gait device for >50% of time walking
		Pain, numbness, or tingling in the lower extremity
		Pregnancy or having given birth in the previous 12 wk
		Unwillingness to refrain from taking nonsteroidal antiinflammatory drugs for 1 or 2 days prior to testing sessions (criterion specific to quantitative sensory testing)^58^
		Inability to attend regular physical therapist sessions
		Inability to complete 12 mo follow-up session*^c^*
		Ehlers-Danlos syndrome, resulting in a precaution for mobilization (added to criteria May 27, 2021)
Baseline assessment	Hip joint pain during Flexion Adduction Internal Rotation Test, confirmed upon physical examination^83^	Hip pain referred from another source, such as the lumbar spine
	Protective sensation in the feet (for quantitative sensory testing)	

*^a^* At the time of study development, there was no consensus on the use of joint mobilization in patients <18 y old; therefore, we excluded those <18 y old. To reduce the likelihood that patients enrolled would have signs of osteoarthritis, we took a conservative approach and excluded those >40 y old.

*^b^* The pain rating was taken at the time of the initial screening for study inclusion.

*^c^* Potential study participants were asked during the screening process if they would be able to return to the testing site for the 12 month follow-up. Given that students were included in the study, there was a potential for them to move away from the testing site if they graduated within the study time.

### Assessment

Patients who met final eligibility criteria completed self-report questionnaires and participated in a clinical examinationPatients were asked to refrain from nonsteroidal antiinflammatory drugs during the 2 days preceding the assessment session to limit their influence on sensory testing. Self-report questionnaires included demographics, medical and hip pain history, the University of California Los Angeles (UCLA) Activity Score, Central Sensitization Index,^40^ the Hip Disability and Osteoarthritis Outcome Score (HOOS)^41^ (effectiveness outcome described below), and patient-specific tasks reported by the patient to be symptom provoking. Patient-specific tasks included daily tasks, such as walking or stair negotiation, fitness, or work-related tasks. Assessment of hip range of motion was performed to assist the treating physical therapists in selecting hip joint mobilization techniques for those randomized to the JtMob group. The following motions were assessed: in the sitting position, internal rotation and external rotation with the knee and hip flexed to 90 degrees^42^; in the supine position: flexion,^43^ flexion adduction internal rotation (FADIR) test (symptoms only),^43^ flexion abduction external rotation test,^44^ abduction,^45^ adduction,^45^ and hip extension.^45^ For extension, the patient was positioned with their buttocks at the edge of the table, their contralateral limb flexed toward the chest, to obtain neutral spine alignment, and the assessed limb lowered below the edge of the mat. For each motion, the examiner moved the hip passively to its end range of motion, determined by a firm end-feel while preventing compensatory motion at the pelvis or knee joint through stabilization or monitoring. The examiner also documented the patient’s report of pain. The range of motion values along with the range of motion at which the patient reported the first onset of pain was used by the treating physical therapist (Suppl. Appendix 1). Movement assessment and quantitative sensory assessment were performed to assess the theoretical mechanism associated with MoveTrain and JtMob respectively, and are described below. Patients were then randomized into JtMob or MoveTrain. Immediately after treatment, patients returned for posttreatment testing.

### Randomization and Blinding

Patients were randomized to 1 of 2 treatment groups in a 1:1 ratio stratified by sex and the HOOS symptoms subscale median (≤ 72.5 or > 72.5) as determined from preliminary data.^46^ Within each stratum, patients were allocated using a block size of 4 to maintain the desired allocation ratio at intervals throughout the recruitment process. A priori, the study biostatistician generated randomization sequences using a formal probability model (with the RANUNI function in SAS software, version 9.4, of the SAS System for Windows [SAS Institute Inc, Cary, NC, USA]) and uploaded them to the data capture system. For each patient who was eligible and consented, the research coordinator obtained the treatment assignment from the data capture system and directed the patient to their assigned group. Personnel who completed assessments and measurements were blinded to treatment assignment; the treating physical therapists and patients were not.

### Treatments

The Template for Intervention Description and Replication Guidelines was used to develop and report treatment provided.^47^ Treatment in both groups included: 10 individualized, supervised visits (30–60 minutes each) with a trained physical therapist provided over 12 weeks; instruction in a home exercise program (HEP); and handouts that provided patient education, general description and benefits of assigned treatment, and specific instructions for their HEP. Both treatment groups included assessment of patient goals and patient education which focused on patient-specific tasks reported by the patient to be symptom-producing. Based on feedback received from physical therapists and patients who participated in our previous study,^46^ we modified HEP parameters used previously to optimize HEP adherence. We decreased the number of tasks/exercises to be performed by each patient during their HEP from 8 to 5 and decreased the recommended frequency of HEP performance from daily to 5 times per week.

#### Joint Mobilization

Full details for JtMob treatment are provided in Supplementary Appendix 1. Focus of treatment was on reducing hip joint pain using joint mobilization techniques provided by the physical therapist and exercises performed in the HEP. Based on published literature,^10^^,^^27^^,^^29^^,^^48–52^ we developed a standard set of mobilizations to target hip joint motion limitations, defined as stiffness or pain that limited joint range of motion. Joint mobilization techniques were prioritized based on patient-specific tasks and the motion used during those tasks, followed by the hip motion limitations. The choice of joint mobilization techniques and grade used was based on direction of hip motion limitation and the relationship of pain and stiffness during hip motion assessment. The HEP included commonly used joint motion and stretching exercises to complement techniques performed during supervised sessions.

#### Movement Pattern Training

Full details for MoveTrain treatment are provided in Supplementary Appendix 2. Focus of treatment was on task-specific training to improve lower extremity movement patterns during patient-specific tasks. Tasks were prioritized based on patient-specific tasks identified by the patient during baseline examination. Exercises included repeated practice of the identified tasks using optimized movement patterns. Based on the patient’s performance, the difficulty of patient-specific tasks was progressed by increasing repetitions performed, increasing load, or changing support surface. The HEP consisted of repeated practice of tasks performed during supervised sessions.

### Outcomes

#### Trial Feasibility

To determine feasibility of future, larger RCT, we assessed patient recruitment and retention, adherence to treatment session attendance, and HEP performance. Recruitment rate was defined as the number of patients determined to be eligible and agreed to participate. Retention rate was defined as the percentage of those enrolled at baseline who completed testing after treatment. Patient adherence to treatment session attendance was defined as the number of patients who attended at least 90% (9 of 10) of their sessions. To assess HEP adherence, patients completed a questionnaire, administered weekly, reporting the percentage of days per week they completed their exercises, the percentage of time per week they followed instructions to modify their patient-specific tasks, and the percentage of prescribed exercises they performed each day. To assess treatment fidelity,^38^^,^^39^ chart reviews were performed to assess physical therapists’ protocol adherence, more specifically their delivery of active treatment ingredients and patients’ treatment receipt, which refers to the patients’ ability to perform treatment exercises/tasks independently.^53^ Independent performance was determined and documented by the physical therapist using our previously published methods.^53^ For each exercise, the physical therapist observed the patient’s performance and asked questions to determine the patient’s ability to perform each exercise independently and to understand key treatment concepts. Additionally, at each patient contact, the physical therapist or research coordinator asked standardized questions about the patient’s symptoms or other changes in medical status to monitor for adverse events.

### Preliminary Effectiveness Outcomes

#### Patient-Reported Outcome Measures

To indicate posttreatment change in impairments (pain and symptoms) and activity limitations, we used the HOOS.^41^ The HOOS is a hip-specific patient-reported outcomes measure representing 5 domains: pain, symptoms, activities of daily living (ADL), sport and recreation, and quality of life. Each subscale, representing a domain, is rated from 0 to 100, with higher scores indicating higher levels of function. The test–retest reliability of the HOOS subscales has been reported to be excellent (ICC = 0.84–0.96).^54^^,^^55^ Construct validity has been established among patients with HRGP, with all HOOS subscales being strongly correlated with 36-Item Short-Form Health Survey questionnaire physical subscales (ρ = 0.568–0.778).^55^ The primary effectiveness outcomes were the HOOS ADL and symptoms subscales. The other HOOS subscales and the International Hip Outcome Tool–33 questions (iHOT-33) were used as secondary effectiveness outcome measures.^56^

#### Two-Dimensional Kinematics

To assess change in the theoretical mechanism associated with MoveTrain, we used our previously published methods^46^ using a digital camera to capture 3 trials of a single-leg squat. Two-dimensional kinematic variables in the frontal plane were assessed, including hip adduction, pelvic drop/hike, and trunk lean. An average of the 3 trials was calculated for each variable. Full description of our methods is provided in Supplementary Appendix 3. Our group has established excellent interrater reliability of these measures [ICC(3,3)^57^ > 0.98; standard error of measurement <1 degree).^46^

#### Pain Pressure Threshold

To assess change in the theoretical mechanism associated with JtMob, we used previously published methods^58^^,^^59^ to assess mechanical pain pressure threshold at the anterior groin of the most bothersome hip (local pressure hypersensitivity) and the dominant thenar eminence (generalized pressure hypersensitivity). We used a Wagner Instruments (Greenwich, CT, USA) FPN 100 analog algometer with a 1 cm^2^ contact area. The algometer was applied perpendicular to the skin surface and stimulus applied in an ascending intensity at a rate of 0.5 kg/cm^2^/s.^58^ Patients were instructed to say stop when the pressure sensation first became a painful sensation. Three trials^58^^,^^60^ were performed at each location and were averaged for analysis. Lower thresholds, indicating increased sensitivity at the hip compared to the thenar eminence, may indicate peripheral sensitization.^61^ Lower thresholds at both the hip and thenar eminence may indicate central sensitization.^61^ Using the described methods, our test–retest reliability values for the pain pressure threshold were high at the hip [ICC(3, 3) = 0.93; standard error of measurement = 0.3] and thenar eminence [ICC(3, 3) = 0.97; standard error of measurement = 0.2], values consistent with previous reports.^62^^,^^63^

#### A Priori Criteria for Success

To indicate a future RCT would be feasible, we expected to achieve the following: 40 patients enrolled, 90% patient retention, 90% treatment session attendance by patients, 80% HEP adherence reported by patients, and 80% protocol adherence by physical therapists. This pilot study was not powered to find statistically significant between-group differences.

### Sample Size

Based on the Cohen^64^*d* effect size, calculations were performed to determine the statistical power of the study assuming that 40 patients were enrolled and 90% were expected to provide complete data at baseline and after treatment.^46^ Within each treatment group, the proposed sample size would have 80% power to detect large effect sizes—0.7 or greater—with 95% CIs for the detected effect size ranging from 0.2 to 1.2. Between-group comparisons of change between baseline and posttreatment phase would have 80% power to detect a minimum effect size of 1.0 (95% CI = 0.3 to 1.6).

### Statistical Analysis

Feasibility outcomes were compared across groups using the Fisher exact test (categorical variables) or the Wilcoxon test (ordinal variables). Secondary outcomes that are continuously distributed and measured at baseline and after treatment were analyzed with analysis of covariance (ANCOVA) using a generalized linear model in which the change in response to treatment (posttreatment minus baseline) is the dependent variable and baseline is the covariate. The adjusted treatment effect was calculated by subtracting the least-squares mean change between baseline and posttreatment data for MoveTrain minus JtMob from the ANCOVA, to assess the between-group difference in change in response for treatment after adjusting for baseline. When data were not normally distributed, nonparametric ANCOVA^65^ was used. For ordinal outcomes (ie, pain rating), between-group differences in change in response to treatment were assessed by ANCOVA using multinomial generalized estimating equations. For all outcomes, adjusted between-group differences in change by ANCOVA are reported with 95% CIs.

For continuous outcomes, *t*-tests for dependent samples were used to test the null hypothesis that the change in response to treatment within each treatment group is not statistically different from 0. When the data were not normally distributed, the Wilcoxon signed rank test was used as the nonparametric equivalent. The data analysis was generated using SAS software, version 9.4, of the SAS System for Windows.

### Role of the Funding Source

Funders played no role in study design, conduct, or reporting of this study.

## Results

### Feasibility

#### Recruitment and Retention Rate

A flow diagram of patient recruitment and retention is provided in the Figure. Table 2 provides baseline characteristics, and Table 3 provides the results related to study feasibility. Patient recruitment occurred between July 2019 and July 2021, and posttreatment assessments were completed by October 2021. Thirty-three patients were randomized, and 29 (88%) completed posttreatment assessments. Six of the 29 patients completed posttreatment questionnaires but were unable to complete posttreatment laboratory testing due to COVID-19 pandemic restrictions. Four patients (2 due to the COVID-19 pandemic) did not complete treatment or posttreatment testing.

**Figure:**
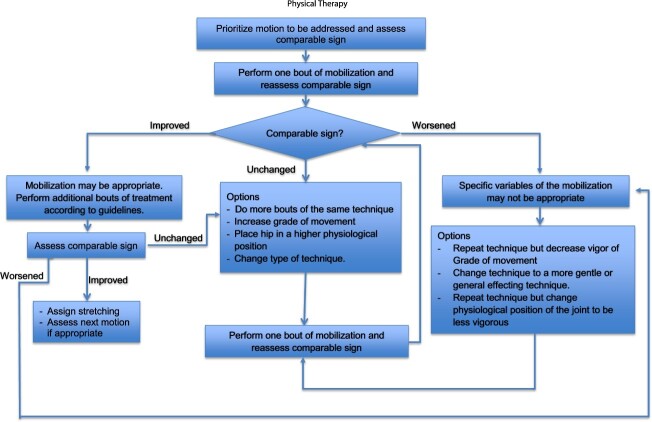
Consolidated Standards of Reporting Trials (CONSORT) flow diagram adapted from Eldridge et al^36^ *The most frequent reasons for ineligibility included pain/numbness/tingling that radiated (*n* = 15), age (*n* = 8), high-impact trauma (*n* = 8), pain in another joint that limited use of stairs/squats (*n* = 8), and modified Harris Hip score (*n* = 8). An individual might be ineligible for more than 1 reason. Not all eligibility criteria were assessed for all individuals. ^†^Reasons for refusal included inability to contact patient to schedule eligibility assessment (*n* = 7), patient was too busy (*n* = 5), patient wanted their own physical therapist (*n* = 2), patient did not want to get a physician’s prescription for physical therapy (*n* = 2), patient was considering surgery (*n* = 1), and patient lived too far away (*n* = 1). ^‡^Reasons for ineligibility included failure of reproduction of groin/hip joint pain in at least 1 hip during the eligibility assessment (*n* = 6) and reproduction of hip joint pain with spine test during the eligibility assessment (*n* = 11). ^§^Of the 15 patients allocated to the joint mobilization (JtMob) group with follow-up ascertained, 2 did not complete the allocated intervention (1 patient completed 7 treatment visits and 1 patient completed 9 treatment visits due to restrictions put in place for patient safety during the COVID-19 pandemic). Of the 14 patients allocated to the movement pattern training (MoveTrain) group with follow-up ascertained, 2 did not complete the allocated intervention (1 patient completed 7 treatment visits and 1 patient completed 9 treatment visits due to restrictions put in place for patient safety during the COVID-19 pandemic).

**Table 2:** Demographics for All Randomized Patients Who Provided Follow-Up Data, Overall and by Treatment Group*^a^*

**Variable**	**Overall Sample (*n* = 29)**	**By Treatment Group**	
		**MoveTrain (*n* = 14)**	**JtMob (*n* = 15)**
Age,*^b^* y			
Mean (SD)	30 (5.7)	31 (6.1)	28 (5.2)
Range	22–39	24–39	22–38
Sex*^c^*			
Male	6 (21)	3 (21)	3 (20)
Female	23 (79)	11 (79)	12 (80)
Race			
White	27 (93)	13 (93)	14 (93)
African American	1 (3)	0 (0)	1 (7)
Biracial: Asian and White	1 (3)	1 (7)	0
Ethnicity			
Hispanic or Latino	2 (7)	2 (14)	0 (0)
Not Hispanic or Latino	27 (93)	12 (86)	15 (100)
Education level*^d^*			
College	17 (59)	7 (50)	10 (66)
Nondoctoral graduate degree	11 (38)	7 (50)	4 (27)
Doctoral degree	1 (3)	0 (0)	1 (7)
Hand dominance			
Right	25 (86)	12 (86)	13 (87)
Left	4 (14)	2 (14)	2 (13)
Measured BMI, kg/m^2^			
Mean (SD)	25.1 (5.3)	26.1 (4.5)	24.2 (6.0)
Range	18.1–41.5	19.5–34.5	18.1–41.5
Study limb			
Left	14 (48)	7 (50)	7 (47)
Right	15 (52)	7 (50)	8 (53)
Limb with pain			
Unilateral left	9 (31)	4 (29)	5 (33)
Unilateral right	8 (28)	2 (14)	6 (40)
Bilateral	12 (41)	8 (57)	4 (27)
UCLA,*^e^* median (range)	9 (4–10)	7.5 (4–10)	10 (4–10)
Pain duration, categorical			
3–<6 months	5 (17)	2 (14)	3 (20)
6–<12 months	5 (17)	2 (14)	3 (20)
1–2 years	8 (28)	4 (29)	4 (27)
3–<5 years	3 (10)	1 (7)	2 (13)
5–10 years	7 (24)	5 (36)	2 (13)
> 10 years	1 (3)	0 (0)	1 (7)
CSI severity			
Subclinical, <29	22 (76)	10 (71)	12 (80)
Mild, 30–39	4 (14)	1 (7)	3 (20)
Moderate, 40–49	2 (7)	2 (14)	0 (0)
Severe, 50–59	0 (0)	0 (0)	0 (0)
Extreme, 60–100	1 (3)	1 (7)	0 (0)

*^a^* Data are reported as numbers (percentages) of patients unless otherwise indicated. BMI = body mass index; CSI = Central Sensitization Index; JtMob = joint mobilization; MoveTrain = movement pattern training; SD = standard deviation; UCLA = University of California Los Angeles Activity Score.

*^b^* Age was self-reported by patients in a screening interview.

*^c^* Sex was self-reported by patients on a self-report questionnaire.

*^d^* Patients were asked, “What is the highest-level formal education that you have completed?” Choices were as follows: elementary school, 9th–11th grade, 12th grade or general equivalency diploma, some college/associate’s degree, college (includes multiple degrees), nondoctoral graduate degree, and doctoral degree.

*^e^* Patients were asked to rate their activity level over the previous 6 months as follows: 10 = regularly participate in impact sports; 1 = wholly inactive, dependent on others.

**Table 3:** Summary of Results for Primary Outcomes Related to Study Feasibility*^a^*

**Variable**	**Overall Sample**	**By Treatment Group**		
		**MoveTrain**	**JtMob**	***P***
Retention and session attendance*^b^*				
Retention rate, no. (%) of patients*^c^*	29 (88)	14 (93)	15 (83)	.61*^d^*
Treatment session attendance, no. (%) of patients*^e^*	28 (85)	13 (87)	15 (83)	1.0*^d^*
HEP adherence and treatment receipt*^f^*				
% of days/week able to complete prescribed exercises*^g^*				.32*^h^*
Median (IQR)	71 (24)	67 (18)	71.1 (25)	
Range	38–100	38–91	37.8–100	
% of times/week followed instructions to modify activities/tasks that aggravated symptoms*^i^*				.10*^h^*
Median (IQR)	82 (19)	74 (13)	88 (24)	
Range	32–100	51–93	32–100	
% of prescribed exercises performed per day*^j^*				.11*^h^*
Median (IQR)	86 (17)	81 (13)	94 (16)	
Range	34–100	34–100	37–100	
% of exercises patients performed independently *^k^*				.003*^h^*
Median (IQR)	93 (11)	88 (12)	98 (8)	
Range	42–100	42–98	90–100	
Treatment fidelity*^l^*				
% of active ingredients of treatment provided by the physical therapists*^m^*				.69*^h^*
Median (IQR)	98 (5)	98 (2)	98 (7)	
Range	87–100	87–100	91–100	
No. of treatment visits				.80*^h^*
Median (IQR)	10 (0)	10 (0)	10 (0)	
Range	7–10	7–10	7–10	

*^a^* HEP = home exercise program; IQR = interquartile range, defined as the 75th percentile minus the 25th percentile; JtMob = joint mobilization; MoveTrain = movement pattern training.

*^b^* Numbers of patients in the overall sample, MoveTrain group, and JtMob group were 33, 15, and 18, respectively.

*^c^* Retention rate was defined as the percentage of patients who were enrolled at baseline and completed testing after treatment.

*^d^* Fisher exact test for group comparison.

*^e^* Treatment session attendance was documented by the physical therapist. The variable for treatment session attendance was the number of patients who attended at least 90% (9/10) of their sessions. Of the 33 patients in the study, 1 in the MoveTrain group (not COVID-19 pandemic related), and 1 in the JtMob group (COVID-19 pandemic related) withdrew prior to starting treatment. One patient in the JtMob group completed 2 treatment visits and withdrew due to COVID-19. One patient in the MoveTrain group and 1 patient in the JtMob group completed 7 treatment visits due to restrictions put in place for patient safety during the COVID-19 pandemic. One patient in the MoveTrain group and 1 patient in the JtMob group completed 9 treatment visits due to restrictions put in place for patient safety during the COVID-19 pandemic. One patient in the JtMob group completed 9 treatment visits and withdrew from the study for reasons other than the COVID-19 pandemic.

*^f^* Numbers of patients completing at least 9 supervised visits. Numbers of patients in the overall sample, MoveTrain group, and JtMob group were 28, 13, and 15, respectively.

*^g^* Patients were instructed to perform their prescribed exercises 5 times/week. This variable was calculated as the average percentage of days/week (divided by 5) over the entire treatment period reported by patients to have completed the prescribed tasks/exercises.

*^h^* Wilcoxon test.

*^i^* Patients in both groups received instructions in methods to modify activities/tasks that aggravated their symptoms. This variable was calculated as the average percentage of time/wk over the entire treatment period reported by patients to have followed those instructions. Patients reported their participation each week.

*^j^* This variable was calculated as the average percentage of prescribed exercises reported by patients to have performed per day over the entire treatment period. Patients reported their participation each week.

*^k^* The total number of exercises the physical therapist reviewed and the total number of those exercises they rated as independent were each summed across all treatment visits to derive the percentage of exercises rated as independent across the entire treatment, ie (sum independent/sum reviewed) × 100.

*^l^* Numbers of patients completing at least 1 supervised treatment visit. Numbers of patients in the overall sample, MoveTrain group, and JtMob group were 30, 14, and 16, respectively.

*^m^* Treatment delivery assessed using chart reviews to determine whether the active ingredients of the treatment were provided. The variable for treatment delivery was the proportion of active ingredients completed and documented by the treating physical therapist for the number of treatment visits.

#### Patient Treatment Adherence

Overall, 28 of 33 patients attended at least 90% of their sessions. These 28 patients reported completing their HEP 71% (range = 38–100%) of prescribed days, following instructions to modify patient-specific tasks 82% (range = 32–100%) of the time and performing 86% of the prescribed exercises each prescribed day.

#### Treatment Fidelity

Regarding physical therapist treatment delivery, the overall proportion of active ingredients completed by the physical therapists during treatment visits provided was 98% (range = 87–100%). Regarding patient treatment receipt overall, the proportion of exercises for which the patients displayed independent performance was 93% (range = 42–100%). There was a significant difference between groups; the JtMob group displayed greater exercise independence than the MoveTrain group (98 and 88%, respectively).

#### Protocol Deviations

We had 3 protocol deviations due to patients’ personal schedules. Six patients were in the treatment phase when the study was suspended. Of the 6 patients, 2 completed all 10 visits, 2 completed 9 visits, and 2 completed 7 visits prior to the suspension. Based on our feasibility study,^6^ we determined, a priori, that completion of 6 of 10 visits would indicate sufficient treatment completion; therefore, we collected their posttreatment questionnaires.

#### Safety

The following adverse events were reported; 1 patient was diagnosed with and received treatment for cancer and 4 patients reported new onset of musculoskeletal pain in another joint/segment that was associated with activity participation but unrelated to treatment.

### Preliminary Effectiveness

Preliminary effectiveness outcomes are provided in Table 4. Both groups reported clinically important improvements in HOOS subscales and iHot-33 (*P* ≤ .01). Patients in MoveTrain displayed improvements in hip adduction (*P* = .025) and pelvic drop (*P* = .044) during a single-leg squat that were greater than measurement error. No changes were noted in trunk lean motion or pain pressure threshold for either group. After adjustment for baseline, there were no between-group differences (*P* > .10) in the change in outcomes in comparisons of JtMob and MoveTrain.

**Table 4:** Summary of Results for Patient-Reported Outcome Measures Assessed at Baseline and After Treatment (*n* = 29)*^a^*

**Variable**	**Baseline** ^***b***^	**After Treatment** ^***b***^	**Unadjusted Within-Group Change** ^***b***^ ^***,***^ ^***c***^	***P*** ^***d***^	**Adjusted Immediate Treatment Effect** ^***e***^ **(95% CI)**	***P*** ^***f***^
HOOS scores						
HOOS pain*^g^*						
MoveTrain (*n* = 14)	72.5 (8.1)	83.4 (9.2)	10.9 (7.7)	.0001	−4.1 (−9.1 to 1.0)	.11
JtMob (*n* = 15)	77.5 (10.5)	90.0 (6.5)	12.5 (7.9)	<.0001		
HOOS symptoms*^g^*						
MoveTrain (*n* = 14)	68.6 (15.1)	77.5 (14.9)	8.9 (9.8)	.005	−4.2 (−11.7 to 3.2)	.25
JtMob (*n* = 15)	74.0 (10.7)	85.0 (9.1)	11.0 (11.5)	.002		
HOOS ADL*^g^*						
MoveTrain (*n* = 14)	80.3 (13.5)	92.0 (9.3)	11.8 (8.3)*^h^*	.0002	0.73 (−2.4 to 3.9)	.65*^i^*
JtMob (*n* = 15)	90.1 (8.2)	96.1 (4.1)	6.0 (5.7)*^h^*	.0001		
HOOS sport*^g^*						
MoveTrain (*n* = 14)	72.3 (23.1)	89.3 (12.4)	17.0 (17.9)*^h^*	.006	3.9 (−3.6 to 11.5)	.31*^i^*
JtMob (*n* = 15)	75.0 (12.3)	86.3 (11.9)	11.3 (13.8)*^h^*	.01		
HOOS QOL*^g^*						
MoveTrain (*n* = 14)	54.9 (13.9)	67.0 (14.0)	12.1 (14.6)*^h^*	.005	−7.1 (−16.2 to 2.0)	.13*^i^*
JtMob (*n* = 15)	50.8 (10.0)	72.5 (12.7)	21.7 (13.3)*^h^*	.0001		
Frontal plane kinematics for study limb,°*^j^*						
Hip adduction angle*^k^*						
MoveTrain (*n* = 11)	24.2 (6.8)	16.7 (7.4)	−7.5 (9.9)*^h^*	.025	−2.9 (−8.7 to 2.8)	.32*^i^*
JtMob (*n* = 11)	21.9 (8.5)	18.8 (7.8)	−3.2 (6.1)*^h^*	.12		
Pelvic drop/hike excursion*^l^*						
MoveTrain (*n* = 11)	11.7 (4.9)	8.4 (3.0)	−3.3 (5.8)*^h^*	.044	−0.76 (−3.7 to 2.1)	.61*^i^*
JtMob (*n* = 11)	11.2 (7.4)	9.0 (4.6)	−2.2 (5.7)*^h^*	.43		
Trunk lean excursion*^l^*						
MoveTrain (*n* = 11)	3.4 (3.4)	2.8 (3.3)	−0.52 (2.8)*^h^*	.81	−0.22 (−2.7 to 2.3)	.86*^i^*
JtMob (*n* = 11)	2.0 (3.5)	2.2 (4.2)	0.18 (3.7)*^h^*	.94		
Pain pressure threshold						
Thenar eminence						
MoveTrain (n = 11)	4.0 (1.1)	3.8 (1.6)	−0.22 (1.1)*^h^*	.46	−0.10 (−0.97 to 0.78)	.78*^i^*
JtMob (*n* = 11)	3.9 (0.74)	3.8 (1.1)	−0.11 (1.1)*^h^*	.21		
Anterior groin						
MoveTrain (*n* = 11)	2.7 (1.4)	2.7 (2.1)	0.03 (2.3)*^h^*	.76	−0.14 (−1.4 to 1.1)	.83*^i^*
JtMob (*n* = 11)	3.1 (1.7)	3.0 (1.0)	−0.08 (1.0)*^h^*	.53		
Overall pain rating*^m^*						
Average pain intensity over past week in study hip, median (IQR)						
MoveTrain (*n* = 14)	4 (3, 5)	1 (0, 1)	−3 (−5, −1)*^h^*	<.001	1.2 (−0.26 to 2.6)	.11*^n^*
JtMob (*n* = 15)	3 (2, 5)	1 (1, 2)	−2 (−3, −1)*^h^*	<.001		
Worst pain intensity over past week in study hip, median (IQR)						
MoveTrain (*n* = 14)	6 (4, 6)	2 (1, 4)	−4 (−5, −1)*^h^*		0.39 (−0.94 to 1.7)	.57*^n^*
JtMob (*n* = 15)	6 (4, 8)	3 (2, 4)	−3 (−4, −1)*^h^*	<.001		
iHOT-33*^m^*						
Total score*^g^*						
MoveTrain (*n* = 14)	56.1 (17.2)	72.9 (20.7)	16.9 (17.1)	.003	−3.2 (−14.6 to 8.2)	.57
JtMob (*n* = 15)	60.6 (14.9)	79.4 (16.5)	18.8 (13.0)	<.001		

*^a^* HOOS = Hip Disability and Osteoarthritis Outcome Score; HOOS ADL = function in activities of daily living; HOOS pain =; HOOS QOL = quality of life; HOOS sport = function in sports and recreation; HOOS symptoms = symptoms; HOOS pain = pain; iHOT-33 = International Hip Outcome Tool–33 questions; IQR = interquartile range (25th, 75th percentiles); JtMob = joint mobilization; MoveTrain = movement pattern training.

*^b^* Data are reported as mean (SD) unless otherwise indicated.

*^c^* Unadjusted within-group change was calculated by subtracting baseline value from value after treatment within each treatment group.

*^d^* *P* values were determined by *t*-tests for dependent samples unless otherwise indicated.

*^e^* The adjusted immediate treatment effect was calculated by subtracting the least-squares mean change between baseline and posttreatment data for MoveTrain JtMob from the analysis of covariance (ANCOVA) to assess the between-group difference in change after adjustment for baseline.

*^f^* Unless otherwise indicated, *P* values were determined by ANCOVA using a generalized linear model in which change was the dependent variable and baseline was the covariate.

*^g^* Patient-reported outcome measure with 100 indicating no disability.

*^h^* *P* value was determined with the Wilcoxon signed rank test.

*^i^* *P* value and adjusted treatment effect were determined by nonparametric ANCOVA.^44^

*^j^* Kinematics represent the mean of up to 3 trials in which the sign of the measurements (ie, negative or positive) was included when computing the average.

*^k^* Hip adduction angle was measured at the lowest depth of the single-leg squat. Larger positive values indicate a larger hip adduction angle.

*^l^* Values for excursion were calculated from the first initiation of descent to the end of descent on the stance leg. For pelvic drop/hike excursion, larger positive values indicate a larger contralateral pelvic drop. For trunk lean excursion, larger positive values indicate trunk lean toward the weight-bearing limb.

*^m^* Patient-reported outcome measure with 0 indicating no pain and 10 indicating worst pain.

*^n^* *P* value was determined by ANCOVA using multinomial generalized estimating equations.

## Discussion

Based on our findings, a large RCT to assess the efficacy of JtMob and MoveTrain may be feasible. We were on target to meet our recruitment and retention goals prior to the COVID-19 pandemic. Collection of posttreatment patient-reported outcomes was high, but laboratory testing for 6 patients could not be performed while on-site activities were suspended. Session attendance was also affected by the COVID-19 pandemic. Patient HEP adherence was greater than 80% in 2 of the 3 components assessed. Treatment fidelity was 98%. Our preliminary findings suggest that 12 weeks of rehabilitation, either JtMob or MoveTrain, is safe and may result in improvements in patient-reported pain and activity limitations.

We previously established the safety and feasibility of MoveTrain.^6^^,^^46^ In this study, we also established the safety and feasibility of JtMob for patients with HRGP. In our proof-of-concept study assessing MoveTrain,^6^ we used a wait-list design. Patients randomized to a 6-week waitlist reported a worsening or no change in their HOOS subscales compared to their baseline^6^; therefore for this study, we decided against including a control group, who would receive no treatment.

Joint mobilization techniques have been used in treatment studies for HRGP^11^^,^^49^^,^^66^; however, treatment description is often insufficient for replication. Most notably, authors did not describe procedures used to guide treatment provider’s decision making while administering joint mobilization throughout the clinical course.^10^^,^^11^^,^^49^^,^^66^^,^^67^ Additionally, it was unclear how the treatment provider uses the patient’s response to a technique to determine treatment parameters.^10^^,^^11^^,^^49^^,^^66^^,^^67^ Although some authors report that pain experienced during treatment is recorded,^49^ few outline pain ratings that are deemed acceptable during treatment or if treatment is modified based on the patient’s pain ratings.^10^^,^^11^^,^^66^^,^^67^ Finally, procedures to assess treatment fidelity among treatment providers often goes unreported. Using common principles associated with joint mobilization, we developed a standardized joint mobilization protocol that allows for individualization of treatment parameters within an RCT. We developed specific guidelines and decision tree for patient assessment and treatment allowing for individualization of treatment parameters that can be implemented consistently among different treatment providers (Suppl. Appendix 1). Techniques used in our study were prioritized based on each patient’s self-identified activity limitations and limited hip motion noted during baseline assessment. Technique parameters were determined by the patient’s response to the technique performed.

Prior to the COVID-19 pandemic, we were enrolling an average of 3 patients per month. This suggests that we could enroll 153 patients during a 5-year project. Given our experience in developing and implementing a multicenter pilot RCT,^46^ a larger sample would be possible. Efforts to increase the diversity of patients enrolled are needed. All participants in our study had a college degree, 93% were White, and 79% were female. During the initial phase of our study, we believed we had a comprehensive recruitment strategy; however, as the study progressed, we determined we were not reaching a diverse population. Approximately 1 week prior to COVID-19 pandemic, we met with an expert in community engagement and devised a recruitment strategy to expand our reach by contacting businesses, volunteer organizations, community centers, and participating local health fairs in neighboring communities. These efforts were limited by COVID-19; however, we will expand our future recruitment efforts to reach a larger, more diverse network of potential patients.

We did not achieve our target retention rate of 90%, which was based on our previous work.^46^ Nevertheless, we collected posttreatment patient-reported outcome measures for 88% of patients, a value that is higher than the 80% required for a high-quality RCT.^68^ Our high retention rate may be partially due to our study location, a large academic healthcare facility, where many potential study patients are engaged in healthcare or research. Other factors that may contribute to our high retention include providing compensation for testing sessions, free treatment, and maintaining consistent contact with each patient during the study.

Attendance to weekly treatment sessions was high, although lower than our targeted 90%^46^ due to COVID-19 pandemic. Further work would be needed to determine if session attendance would be affected if visits were more or less frequent. Based on our previous study,^46^ we modified the HEP, and therefore expected high HEP adherence (80%). Patients reported high adherence in 2 of the 3 assessed HEP components, the percentage of time/week they followed instructions to modify the activities/tasks that aggravated their symptoms (82%), and the percentage of prescribed exercises performed each day (86%). They reported slightly lower adherence (71%) in the percentage of days/week they completed their HEP. It may be easier for patients to incorporate activity modifications into their daily lives than it is to perform a specific set of exercises each day. We must acknowledge the limitations of self-report adherence data, including the potential of patients either overestimating or underestimating their actual HEP participation.^69^

We implemented a number of strategies to optimize treatment fidelity among treating physical therapists. We developed a manual of operations that included specific assessment and treatment guidelines for each treatment arm. During training, we obtained feedback from the physical therapists to ensure the guidelines we proposed were consistent with their practice and would be feasible to use. Any perceived barriers to performing the treatment protocol were discussed and resolved prior to study start. Regular communication among the protocol developers and physical therapists occurred to address any issues noted during the treatment sessions. Finally, we developed standard documentation forms that are easy to complete during the treatment session; they serve to collect information related to treatment and also provide cues to remind physical therapists of specific treatment elements. We believe these methods would be useful for future RCTs.

Patients displayed excellent independence, aka patient receipt, in HEP performance. The JtMob group displayed higher independence than the MoveTrain group, 98 and 88%, respectively. This may be due to the complexity of the exercises/tasks assigned. In MoveTrain, instructions for each task included the steps to perform the task and key concepts related to movement quality. In JtMob, instructions for each exercise were focused on steps to perform the exercise; no specific instruction on movement quality was provided. For a patient in MoveTrain to be independent, they must be able to replicate the activity and display the corrected movement pattern. This can be difficult for some patients, particularly early in the treatment phase.

Given the nature of the pilot RCT, the study was not powered to detect significant between-group differences in secondary outcome measures. Both groups reported improvements in all HOOS subscales, suggesting both groups improved in their hip-specific function. The MoveTrain group displayed improvements in their movement quality during the single-leg task; however, neither group displayed changes in pain pressure threshold. These findings are interesting; however, we are hesitant to speculate on their meaning given the small sample size; 29 patients completed the HOOS and 22 patients completed posttreatment assessments of movement and pain pressure threshold. A larger, powered study is needed to further explore these findings. Inclusions of an attention control group or a sham treatment in future studies may provide additional information related to patient outcomes and potential mechanism.

### Limitations

Our patient recruitment and retention were negatively affected by the COVID-19 pandemic. We considered using telehealth methods; however, we ultimately decided against these to maintain equal patient–personnel contact among both groups. MoveTrain may be performed using video and webcast software; however, JtMob requires the patient to be with the physical therapist.

Recent consensus statements recommend the iHOT-33 or the Copenhagen Hip and Groin Outcome Score (HAGOS) for use as patient-reported outcomes measures in patients with HRGP.^70^ Our investigations were initiated prior to these publications, and based on the best evidence at the time, we selected the HOOS subscales as our primary patient-reported outcomes measures. We used a clinically feasible method using a digital camera to assess 2-dimensional kinematics. Two-dimensional measures are poorly correlated with measures from sophisticated 3D motion analysis and can be affected by out of plane movement.^71^ Additionally, we used an average of 3 kinematic trials for our analysis, to be consistent with our previous work.^46^^,^^72–74^ Other methods, such as investigating movement variability across trials, may be worth investigating. The clinical relevance of kinematic changes at the hip and pelvis has yet to be determined. In our previous proof-of-concept study, however, we noted that a patient’s ability to reduce hip adduction motion was associated with patient-reported outcomes after MoveTrain.^7^

Given the paucity of evidence related to the HEP that should be recommended to complement joint mobilization, we developed the HEP based on clinical expertise, which may limit the internal and external validity of the study. It is unlikely 1 single mechanism will explain a patient’s response to joint mobilization. The proposed mechanisms underlying the therapeutic effects of manual therapy and the interactions of these mechanisms are complex,^12^^,^^13^^,^^75^^,^^76^ therefore we refer the reader to the model proposed by Bialosky et al^12^^,^^13^ for a thorough overview and research recommendations. Nevertheless, in this pilot study, we focused on the potential effects of JtMob on peripherally and centrally mediated pain mechanisms, using an indirect measure, quantitative sensory testing. Other potential mechanisms should be considered, and the appropriate measures (eg, imaging, somatosensory evoked potentials, inflammatory mediators, and other quantitative sensory tests, including those recommended by the German Research Network on Neuropathic Pain) should be used.^58^ Additionally, for either treatment, we cannot account for other factors that may be associated with treatment outcomes, such as placebo or nocebo effect, patient factors, and patient-provider interactions. The inclusion of an attention control or sham intervention may provide further insight to outcomes in the future trial.^77^ Finally, we are unable to report the use of medication by patients during the trial; however, we will collect these data in the future trial. The primary purpose of this pilot study was to assess feasibility. A future larger study will benefit from inclusion of additional measures.

We developed this pilot RCT to assess the feasibility of completing a larger RCT to assess the effects of JtMob and MoveTrain. The COVID-19 pandemic limited our patient recruitment, patient retention, and treatment session attendance. Nevertheless, our enrollment rate prior to the pandemic and our overall retention rate suggests that a larger trial may be feasible, barring future pandemics. We established high treatment fidelity among treatment providers and patients. Based on our preliminary findings, 12 weeks of rehabilitation, either JtMob or MoveTrain, is safe and may result in improvements in patient-reported pain and activity limitations. Further investigation is needed to determine the long-term effects of each treatment and if specific patient factors are associated with treatment prognosis.

## Supplementary Material

a_appendix_1_final_pzad111

b_appendix_2_final_pzad111

c_appendix_3_final_pzad111

## Data Availability

Data will be shared with qualified users under an agreement that provides for: (1) a commitment to using the data only for research purposes and not to identify any individual participant; (2) a commitment to securing the data using appropriate computer technology; (3) a commitment to destroying or returning the data after analyses are completed; and (4) a requirement to acknowledge the funding source and research team that generated the data in publications and presentations resulting from the shared dataset.

## Clinical Trial Registration

This trial is registered at Clinicaltrials.gov (NCT03959319).
