# Supplementary material for: Comparison of Joint Mobilization and Movement Pattern Training for Patients With Hip-Related Groin Pain: A Pilot Randomized Clinical Trial
Source: Phys Ther. 2023 Aug 22;103(11):pzad111. doi: 10.1093/ptj/pzad111 (PMC10683042; doi:10.1093/ptj/pzad111)
Supplement: a_appendix_1_final_pzad111 [file a_appendix_1_final_pzad111.pdf]

Appendix 1: Joint Mobilization Protocol

The procedures used in this treatment arm were developed by two physical therapists with specific interest in hip-relate groin pain (HRGP). One is a Fellowship-trained manual therapist with 37 years of experience in clinical practice and in teaching principles and techniques of manual therapy in both entry-level, post-graduate manual therapy and movement based fellowship programs. The second is a clinical investigator with 19 years of musculoskeletal experience and expertise in clinical trial development and implementation.[1,2] Resources used to develop the joint mobilization protocol included previous publications[3-8], fellowship training coursework, and communications with expert manual therapists. Key elements for the protocol were determined and guidelines developed to standardize assessment, decision-making and treatment procedures among treatment providers. A decision tree describing the assessment and decision-making procedures is provided in Figure 1.

- Joint mobilization principles and techniques
    - Handout used by the physical therapist providing the general overview of the principles and techniques used in the study.
- Decision tree for physical therapists – Figure 1
    - Decision tree demonstrating physical therapist's assessment and decision-making procedures related to joint mobilization techniques.
- Treatment techniques – Table 1
    - Handout used by the physical therapist with techniques and stretching exercise to use per the patient's hip joint restrictions, defined as stiffness or pain that was limiting joint range of motion
- Patient education
    - Handouts provided to each patient during their first treatment session.
- Exercise instructions
    - Handouts with exercise instructions that were provided to a patient as each exercise was prescribed.

Joint Mobilization
Principles and Techniques

Overall Goals of treatment

1. Reduce pain by decreasing peripheral and central sensitization.
2. Increase pain-free range of motion of the hip joint and surrounding soft tissues.

General Guidelines for Joint Mobilization Protocol

Prior to meeting the patient

Review the patient's chart for information collected during the baseline assessment.

1. The treatment provider reviews patient demographic, patient-reported outcomes and other patient-specific information.
2. In the patient's chart, the examiner provides range of motion (ROM) values that indicate the patient's first onset of pain (P1) and the end ROM of the joint, which is characterized by a restriction that stops further motion (R2). This information will assist the treatment provider in determining treatment priorities.
3. The treatment provider will prioritize techniques to be used based on Baseline exam ROM information and patient-specific task limitations.
   a. For visit 1 – treatment provider will use max, 2 manual techniques
   b. Patient-specific tasks will be prioritized over the ROM limitations.
   c. ROM – most limited ROM

Communication Guidelines

Continuous communication between the patient and treatment provider is one of the key elements to the successful application of passive joint mobilization techniques. Using previously proposed methods,[3,5,6] we developed specific guidelines for pain monitoring and treatment modification.

1. Communication during the initial assessment on Day 1
   a. For each passive motion performed, the treatment provider will
      i. tell the patient what they are going to do.
      ii. explain the direction they are going to move their leg.
      iii. ask the patient to report the onset of pain as soon as they are aware of it (P1)
      iv. question the patient regarding location of symptoms and severity while they are moving the leg.
      v. confirm with the patient that it is okay to continue the test motion, based on their current pain.
      vi. explain to the patient that the motion may need to proceed beyond the onset of pain (P1) to thoroughly assess the joint. As the motion is continued, the patient is encouraged to report if the pain changes. This will allow the treatment provider to assess R1, P2 and R2 as needed.
      vii. always be ready to halt and reverse the motion per patient request.

2. Symptoms monitoring throughout the treatment session
   a. The patient is instructed in the description of mild, moderate or strong pain. The patient will use this scale to communicate with the treatment provider about where the treatment provider is able to treat within this scale.

        i. Mild = it is painful but very tolerable.
        ii. Moderate – it is painful and the patient can tolerate for a short period of time.
        iii. Strong = it is very painful and cannot be tolerated.
    b. The presence of symptoms should be documented prior to starting the technique, even before placing hands on the patient.
    c. Initially, the patient should be in a position that is non-painful or no worse than that reported in 2a.
    d. As techniques are progressed, the treatment provider will decide when and to what vigor the technique will be performed, based on the patient's pain tolerance. Permission to progress the technique must be acquired from the patient.
    e. The treatment provider asks the patient frequently during the technique whether the symptoms are changing.
    f. The treatment provider obtains pain report after performance of technique. Hip should be in resting position.

Treatment approach

Technique selection: We **used published literature[4,9-11] to develop a standard set of joint mobilizations to target hip joint impairments, defined as stiffness or pain that is limiting joint range of motion (See Table 1). Joint mobilization techniques used are prioritized based on each patient's self-identified functional limitations, and hip mobility impairments determined during the baseline examination.**

Technique progression: **We chose a joint mobilization approach that emphasizes the use of gentle passive techniques to reduce pain early in the course of treatment, then progressing to more vigorous techniques to treat joint mobility impairments.[6] This progression, focusing on pain reduction first, followed by improving joint impairments by using higher grades of joint mobilization has been successfully used in other conditions, such as low back pain,[12] frozen shoulder,[13,14] Post-colles fracture[15] and knee osteoarthritis.[3] In the presence of moderate to severe hypomobility, passive joint mobilization techniques performed at more vigorous ranges of motion have shown efficacy in reducing pain, improving range of motion and function.[12,15-17] We believe this progressive approach builds rapport with the patient by developing a specific communication pattern to be used throughout the treatment.**

1. General procedures
    a. At least two, however no more than five, manual techniques must be performed at each visit.
    b. Manual techniques to be used with each patient will be prioritized based on the restrictions, defined as stiffness or pain that is limiting joint range of motion, noted on baseline examination.
    c. At the first session, the treatment provider will begin with the first two physiological joint motions identified during the baseline assessment.
    d. Technique parameters will be based on the direction of restriction and pain (P)/restriction (R) pattern. In order to determine which grade of movement to use, the treatment provider will need to asesss the following values:
        i. R1 is the first onset of restriction (resistance).
        ii. P1 is the first onset of patient-reported pain.
        iii. R2 is the end range of the joint that is characterized by increased restriction that stops further motion. This may be at the end of normal motion for the hip or the patient may have decreased end range due to hypomobility.
        iv. P2 is the limit of pain that is tolerable to the patient.

2. Specific Assessment: The treatment provider will
    a. assess baseline pain using a numeric rating scales (0 being no pain or symptoms and 10 being worst imaginable).
    b. assess comparable sign, which is the passive physiological range of motion of the hip joint and the presence of pain. The treatment provider will determine onset of P1 in relation to R1.
    c. assess accessory motion and determine stiffness and pain response using the onset of P1 in relation to R1.
    d. select parameters based on the stiffness/pain relationship:
        i. If R1 occurs before P1, choose Grade III, IV or V joint mobilization. In this case, you may be able to progress the passive motion to determine the end range or R2 and P2.
        ii. If P1 occurs before R1, choose Grade I and II joint mobilization.
        iii. If P1 occurs at the same time as R1, begin with Grade I and II. Mobilization may be progressed based on the patient's response.
    e. begin with more conservative approach and then progress, based on patient tolerance.
    f. perform 1 bout of mobilization and monitor symptoms.
    g. re-assess comparable sign.
    h. use Figure 1 to determine if technique should be continued or modified.
3. Treatment: The treatment provider will
    a. decide if they are treating primarily pain or stiffness and will choose the vigor of the joint mobilization techniques based on their assessment. All patients will initially be treated in a non-pain aggravating manner. The vigor and direction of technique will be progressed based on the patients' response to initial treatment. Monitoring of the patient's response will be performed before, during and after each manual technique.
    b. monitor symptoms throughout performance of the passive technique. During manual techniques, patients may experience some pain, however their pain should be no more than minimal. If pain is more than minimal during the execution of the technique, the treatment provider will revise the technique.

4. Parameters for each technique: The treatment provider will determine the following parameters for each technique, based on the patient's chief complaint and associated comparable signs during assessment.
    a. Patient position: standard or modified
        i. Supine: the patient may need pillow support under the knee of the affected leg to reduce extension of the hip at rest.
        ii. Prone: The patient may need pillow support under the pelvis to reduce extension of the hip at rest.
    b. Joint position: resting/neutral, mid-range, end-range
        i. The treatment provider will use resting/neutral position for patients that have limited physiological motion due to pain. As the patient improves, they may progress the physiological motion into a higher range to perform the technique.
        ii. When the patient has at least 50% of passive physiological range of motion that is non-painful, the treatment provider will perform techniques into the highest range the patient comfortably tolerates.
        iii. End range techniques should be performed when the patient has minimal symptoms in the early and mid range and/or have shown improvement with earlier progression of treatment.
    c. Direction of force:
        i. Accessory motions include distraction and glides

ii. Physiologic motions include flexion, extension, abduction, adduction, internal rotation, external rotation
iii. Begin with straight plane motions. You may progress to combining motions that are limited based on the patient's response.
iv. If combined motion is progressed to include greater than 50% of a second motion. Stop and check comparable sign, then change to technique to address second motion.

d. Grades of movement:
   i. Goal to reduce pain: Grades I and II
      1) Oscillatory or sustained: 5-10 seconds; 2-4 bouts
   ii. Goal to reduce stiffness: Grades III, IV and V.
      1) Oscillatory or Sustained: 10-60 seconds; 4-10 bouts
   iii. Thrust is a high velocity, low amplitude movement performed once per treatment session.

5. Technique modification: Each joint mobilization technique may be modified by altering the following variables based on the clinical decision of the treatment provider.
   a. Physiological position of the hip joint in relation to the direction that the technique improves. For instance, lateral glide with the hip in 90° flexion may be progressed by moving the hip into 5-10° of adduction. A caudal glide at 90° flexion may be progressed to 100° flexion or higher depending on pain levels and patient response.
   b. Add variations of direction to the passive glide techniques.
   c. Caudal glide may be performed perpendicular to the treatment plane of the acetabulum thus effecting distraction in the hip. The treatment provider may direct the caudal glide into some amount of lateral or posterior glide if noted that these motions are hypomobile or relate to the physiological motion that is restricted.
   d. Lateral glide may be performed directly lateral to the acetabulum. The treatment provider may add some posterior or caudal direction to the lateral glide if noted that these motions are hypomobile or relate to the physiological motion that is restricted.
   e. Combine physiological motions to progress the vigor of the techniques.
   f. Change the grade of movement and length of time mobilizing.
   g. Alter the resting position of the patient, for instance, from prone to sitting.
6. Therapeutic stretches
   a. must be performed at each visit. These stretches may be performed independently by the patient or with assistance of the treatment provider.
7. Home program
   a. will include flexibility exercises performed during the supervised session. The treatment provider will give written instructions with pictures of the exercises and document changes/additions to the home program at each session.

TECHNIQUES

Joint Mobilization Techniques: Techniques will be prioritized based on restrictions noted on baseline examination.

1. Caudal glide
2. Lateral glide
3. Posterior glide
4. Mobilization with movement (hip flexion)
5. Quadrant (Flexion, Adduction, Internal rotation)
6. Anterior Glide
7. Internal Rotation in Prone

Below is a description of all technique variations that may be performed with each technique described from **least vigorous to most vigorous.** Each technique will be described based on the physiological motion to be improved. The Impaired hip motions are listed below based on the Joint Mobilization Task Table document.

1. **FLEXION**

a. Passive Physiological Flexion can be performed passively through all ranges of motion. This technique may be performed in Grade I, II, III, IV. The motion can be altered by adding some amounts of Abduction, Adduction, Internal or External rotation.

b. Caudal glide: Caudal glide can be performed in any degree of hip flexion. In lower ranges of flexion, the treatment provider may hold the patients lower leg above the medial and lateral malleoli. This technique can be performed in Grade I, II, III, IV and V. In higher ranges of hip flexion, the handhold will be close to the anterior hip joint line and performed in Grade III, IV.

c. Posterior glide: Posterior glide can be performed in any degree of hip flexion. In lower degrees of flexion, the treatment provider may have the patient resting the knee on a pillow. One hand will be placed on the anterior hip medial to the greater trochanter. The other hand will be under the distal thigh. Grade I, II, III, IV can be performed in this position. In higher positions of flexion, the treatment provider will change the handhold to clasping hands over the anterior knee. This glide can be performed in varying positions of abduction, adduction, internal and external rotation. Perform Grade III and IV as the range of motion increases. .

d. Mobilization with Movement (MWM): This technique is performed in higher degrees of hip flexion, approximately 70° to end range flexion. This glide can be performed in varying positions of abduction, adduction, internal and external rotation with Grade III and IV.

e. Quadrant: This technique can vary in the degree of all component motions of flexion, adduction and internal rotation. Use Grade III and IV.

2. **EXTERNAL ROTATION**

a. External rotation in supine: This technique can be performed passively through all ranges of motion. The motion can be altered by adding some amounts of Abduction and Flexion. This technique may be performed in Grade I, II, III, IV.

b. External rotation in prone: This technique can be performed in approximately 30° hip flexion (supported with pillows) to 10° extension. Grade I, II, III, IV can be used. The treatment provider will stabilize the ipsilateral pelvis with one hand on the posterior ilium/buttock.

c. Caudal glide: Caudal glide can be performed in any degree of hip flexion and external rotation. In lower ranges of flexion, the treatment provider may hold the patients lower leg above the medial and lateral malleoli. This technique can be performed in Grade I, II, III, IV and V. In higher ranges of hip flexion and/or external rotation, the handhold will be close to the anterior hip joint line being sure to maintain the desired physiological position of the joint. In this position, Grade III and IV will be used.

d. Anterior glide in prone: Can be performed in any degree of hip flexion/extension, external rotation and abduction. Grade I, II, III, IV can be used.

3. **INTERNAL ROTATION**

a. Internal rotation in supine: This technique can be performed passively through all ranges of motion. The motion can be altered by adding some amounts of Adduction and Flexion. This technique may be performed in Grade I, II, III, IV.

b. Internal rotation in prone: This technique can be performed in approximately 30° hip flexion (supported with pillows) to 10° extension. Grade I, II, III, IV can be used. The treatment provider will stabilize the contralateral pelvis with one hand on the posterior ilium/buttock.

c. Caudal glide: Caudal glide can be performed in any degree of hip flexion, adduction and internal rotation. In lower ranges of flexion, the treatment provider may hold the patients lower leg above the medial and lateral malleoli. This technique can be performed in Grade I, II, III, IV and V. In higher ranges of hip flexion and internal rotation, the handhold will be close to the anterior hip joint line being sure to maintain the desired physiological position of the joint. In this position, Grade III and IV will be used.

d. Lateral glide: Lateral glide can be performed in varying degrees of hip flexion, adduction and internal rotation. Grade I, II, III, IV can be used.

e. Quadrant: This technique is performed in higher degrees of hip flexion from 70° to 110°. The addition of hip internal rotation may be the secondary motion introduced followed by adduction. The handhold is both hands over the patient’s knee with forearms controlling the thigh and lower leg motion. Grade III, IV can be used.

4. **ADDUCTION**

a. Adduction: This technique can be performed through ranges from 10° hip extension and up to 110° hip flexion. Use Grade I, II, III, IV. In higher degrees of flexion, use Grade III and IV.

b. Caudal glide: Caudal glide can be performed in any degree of hip flexion and adduction. In lower ranges of flexion, the treatment provider may hold the patients lower leg above the medial and lateral malleoli. This technique can be performed in Grade I, II, III, IV and V. In higher ranges of hip flexion and adduction, the handhold will be close to the anterior hip joint line being sure to maintain the desired physiological position of the joint. In this position, Grade III and IV will be used.

c. Lateral glide: Lateral glide can be performed in varying degrees of hip flexion and adduction. Grade I, II, III, IV can be used.

d. Posterior glide: Posterior glide can be performed in any degree of hip flexion. In lower degrees of flexion, the treatment provider may have the patient resting the knee on a pillow. To emphasize Adduction, the hip may be placed in some degree of adduction during the glide. One hand will be placed on the anterior hip medial to the greater trochanter. The other hand will be under the distal thigh. Grade I, II, III, IV can be performed in this position. In higher positions of flexion, the treatment provider will change the handhold to clasping hands over the anterior knee. This glide can be performed in varying positions of adduction. Perform Grade III and IV in this position.

e. Quadrant: This technique is performed in higher degrees of hip flexion from 70° to 110°. The addition of hip adduction may be the secondary motion introduced followed by internal rotation. Grade III and IV can be used.

5. **ABDUCTION**

a. Abduction: This technique can be performed through all ranges of hip extension to 0° and flexion up to 110°. Use Grade I, II, III, IV.

b. Caudal glide: Caudal glide can be performed in any degree of hip flexion and abduction. In lower ranges of flexion, the treatment provider may hold the patients lower leg above the medial and lateral malleoli. This technique can be performed in Grade I, II, III, IV and V. In higher ranges of hip flexion and abduction, the handhold will be close to the anterior hip joint line being sure to maintain the desired physiological position of the joint. In this position, Grade III and IV will be used.

6. **EXTENSION**

a. Extension in prone: Patient lies prone with enough pillows under the trunk to flex the hip to a comfortable, least painful position. The treatment provider holds the anterior distal thigh and stabilizes the ipsilateral posterior pelvis. As the range of motion improves, the treatment provider can decrease pillow support allowing the hip to move into more extension. Grade I, II, III, IV can be used.

b. Caudal glide: Caudal glide can be performed in the lower degrees of hip flexion from 20-30°. Grade I, II, III, IV and V can be used. The treatment provider will hold the patients lower leg above the medial and lateral malleoli.

c. Anterior glide in prone: Patient lies prone with enough pillows under the trunk to flex the hip to a comfortable, least painful position. The treatment provider holds the anterior distal thigh and places the palm of their hand along the posterior head of the femur. Grade I, II, III and IV can be used. As the patient progress, the pillows can be removed allowing the technique to be performed in greater degrees of hip extension. Abduction and external rotation can be added to the glide.

7. FABER: Patient lies supine as the treatment provider passively performs hip flexion, abduction and external rotation in varying degrees determined by patient tolerance.

8. FADIR: Patient lies supine as the treatment provider passively performs hip flexion, adduction, and internal rotation in varying degrees determined by patient tolerance.

1. **Caudal Glide**

   Uses: Decrease pain, Increase general mobility

   Comparable Sign: Any limited or painful movement especially flexion when performed in flexion.

   Patient Position (Figure A): Performed in Neutral Hip position: Supine with the hip in the resting position (30˚flexion, 30° abduction, and slight lateral rotation). **Although not required, a stabilization belt may be placed over groin and under the body, attaching to the leg of the plinth. When using a belt, a towel may be placed over the groin area for comfort.

   Treatment Provider Position and Hand Placement: Grasp medial and lateral ankle above distal tibia and fibula with fingers around the posterior ankle. While holding the ankle lean, backwards to transmit a caudal glide. In the presence of knee dysfunction, face the patient and grab the distal thigh near the femoral condyles. The treatment provider may include the addition of varying degrees of hip abduction or external rotation.

   Direction of Movement: Caudal glide. This technique may be angled to pull perpendicular to the treatment plane of the hip joint to effect traction of the hip joint.

   Grades of Movement: Grades I-V.

   Grade V: This technique is performed in approximately 30° flexion and 10-20° abduction. The treatment provider will perform Grade IV or III glides and assess the patients' pain response. If the patient has more than "mild" pain, the thrust should not be performed. If the patient is tolerant of the position with a grade III/IV glide, the treatment provider can perform a high velocity, low amplitude movement.

   The treatment provider will take up the slack in the hip joint and assess symptoms. Release the glide and repeat 3 times to feel the range of motion and patient response. The treatment provider will then take up the slack and perform a quick HVLA motion then re-test the comparable sign.

Figure A

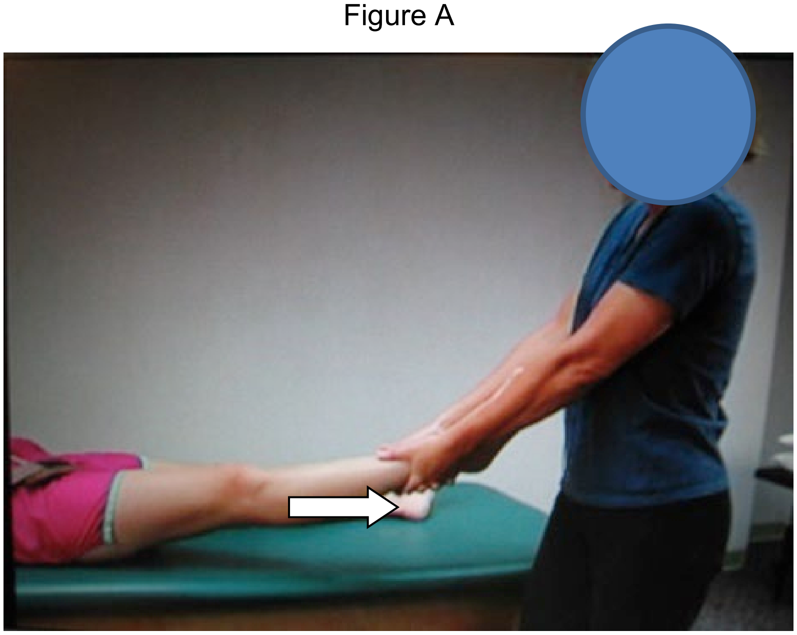

Caudal Glide, continued:

Patient Position (Figure B): Place knee over the treatment provider's shoulder and keep the quadriceps relaxed. Place a towel over the groin area. A lumbar support may be needed to prevent lumbar flexion. The treatment provider may include the addition of varying degrees of hip abduction or external rotation.

Treatment Provider Position and Hand Placement: Standing on the side of the hip to be mobilized. Fingers clasped with ulnar borders of the hands at the most proximal and anterior aspect of the femur. The treatment provider stands in a "walk" stance position to shift weight from front foot to back foot as the caudal glide is applied.

Direction of Movement: Caudal

Grades of Movement: III and IV

Figure B

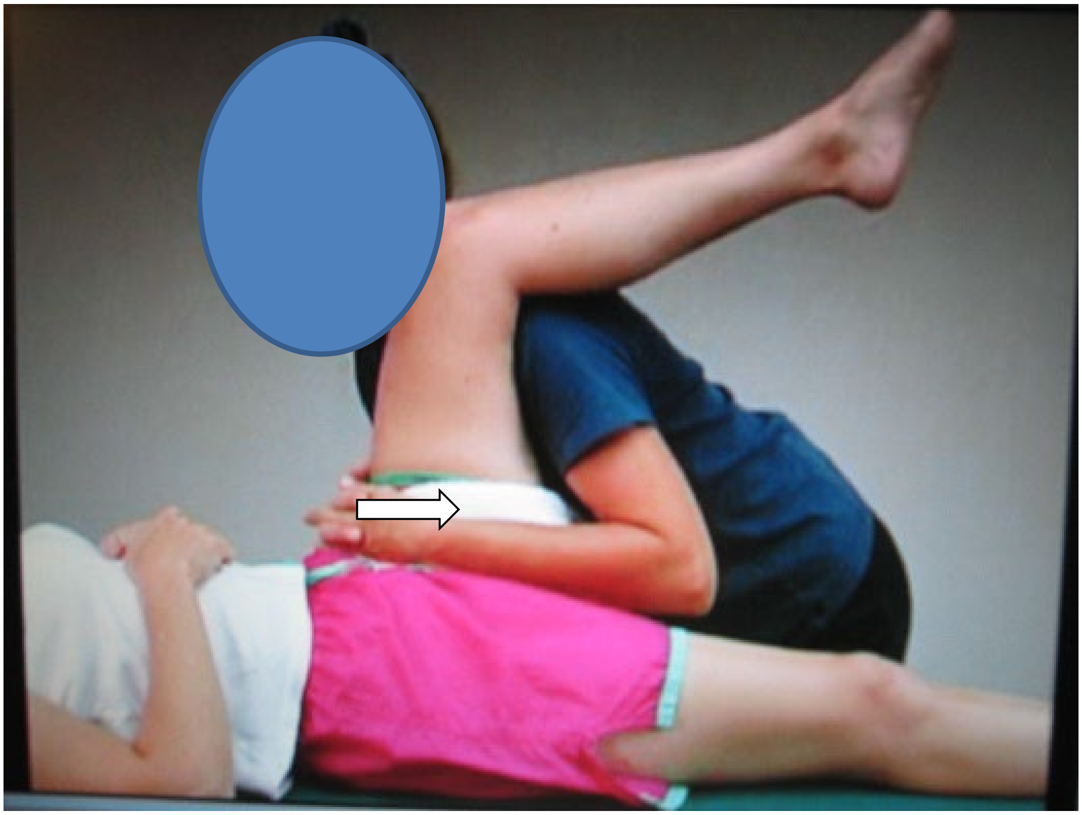

2. Lateral Glide in Flexion

Uses: Decrease pain, Increase flexion, internal rotation and adduction.

Comparable Sign: Painful or limited hip flexion, internal rotation and/or adduction.

Patient Position (Figure C): Supine with hip in flexion and knee flexed. The thigh rests on the treatment provider's chest. A towel is placed over the groin for comfort. A lumbar support may be needed to prevent spinal flexion. The treatment provider may include the addition of varying degrees of hip adduction, flexion and/or internal rotation.

Treatment Provider Position and Hand Placement: Standing on the side of the hip to be mobilized. Fingers clasped with ulnar borders of the hands at the most proximal and medial aspect of the femur in the groin. The treatment provider is in a "walk" stance position.

Direction of Movement: Lateral glide

Grades of Movement: III and IV

Figure C

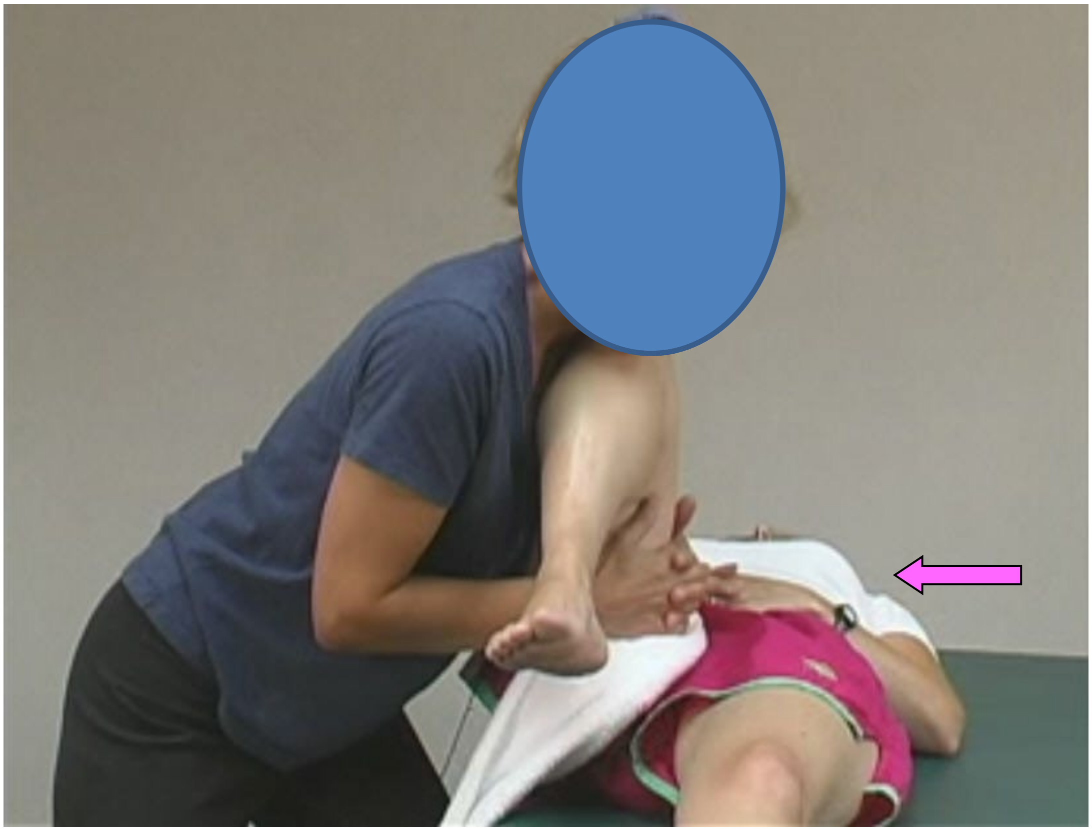

3. **Posterior Glide**

Uses: Decrease pain, Increase adduction and flexion by stretching the postero-lateral capsule.

Comparable Sign: Painful or limited hip flexion or adduction.

Patient Position (Figure D): Supine with hip in flexion. The treatment provider may include the addition of varying degrees of hip adduction, flexion and/or internal rotation.

Treatment Provider Position and Hand Placement: Standing on the side of the hip to be mobilized. Both hands cupped over the knee. Treatment provider's forearms are in line with the long axis of the femur.

Direction of Movement: Posterior glide.

Grades of Movement: Grade III and IV

Figure D

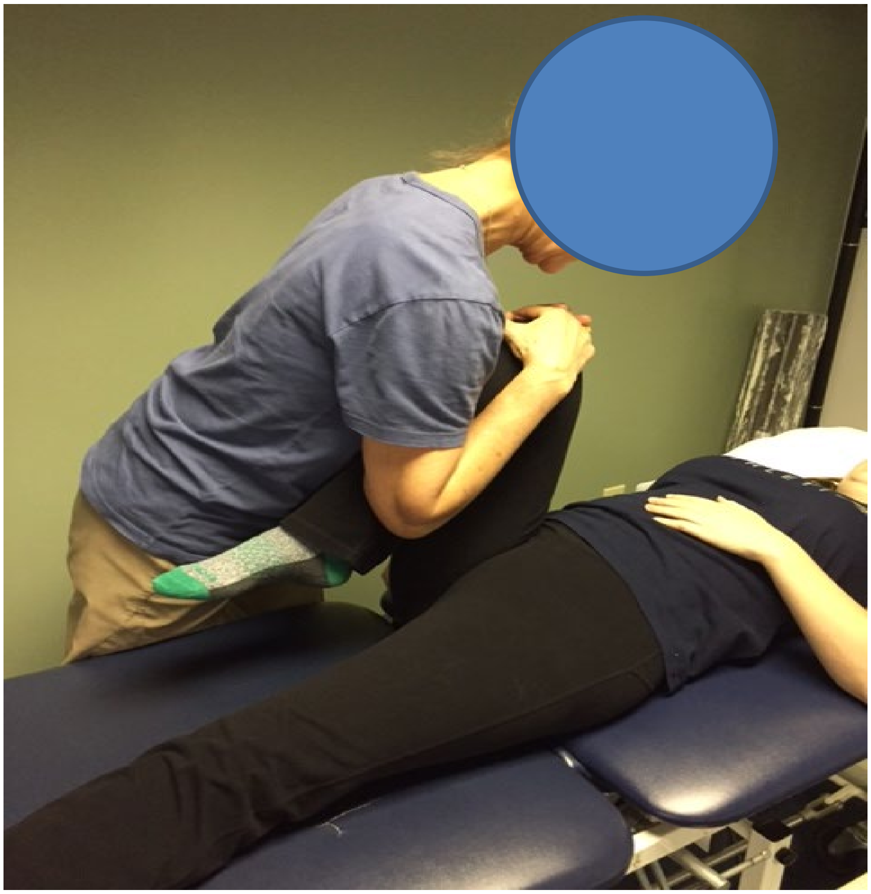

4. **Mobilization with movement (Caudal glide): Hip Flexion**

Uses: Decrease pain, Increase hip joint flexion ROM

Comparable Sign: Painful or limited hip flexion

Patient Position (Figure E): Supine with the hip in 90° of flexion and knee relaxed in a flexed position. A lumbar support may be needed to prevent lumbar flexion during the mobilization.

Treatment Provider Position and Hand Placement: Standing on the side of the hip to be mobilized. Wrap a belt around the treatment provider's pelvis and the patient's proximal thigh as close to the joint line as possible. The belt should be snug enough so that the patient's thigh can be supported by the treatment provider. Hand placement may vary depending on the size of the patient's thigh. For a larger thigh, place stabilizing hand on the proximal lateral side of the hip/thigh and the guiding/mobilizing hand on the top and medial side of the knee. For smaller thighs, the treatment provider may wrap the guiding/mobilizing arm and hand around the distal thigh and proximal knee and the stabilizing hand remains at the proximal lateral side of the hip/thigh.

Direction of Movement: Caudal glide. The treatment provider applies a sustained caudal glide by gently leaning pelvis away from patient and then instructs the patient to actively flex their hip, stopping before onset of pain. One hand guides the motion and the other hand monitors the pelvis to prevent compensatory pelvic tilt. The treatment provider should maintain the glide as the patient extends hip back to the starting position.

Figure E.

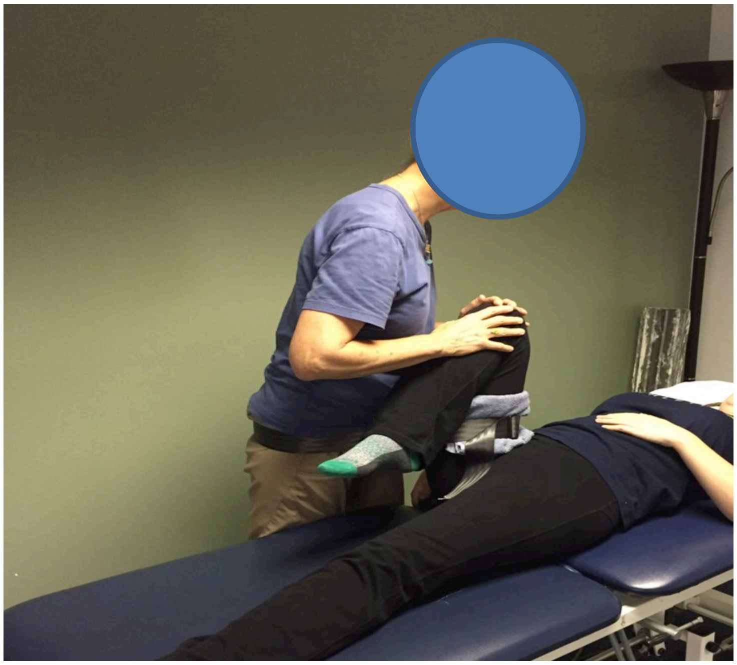

5. **Quadrant (FADIR)**

Uses: Decrease pain, Increase joint play, Decrease stiffness in the combined movements of flexion, adduction and internal rotation.

Comparable Sign: Painful or limited combination of hip flexion, adduction and internal rotation

Patient Position (Figure F): Supine with hip in determined amount of hip flexion, adduction and internal rotation.

Treatment Provider Position and Hand Placement: The treatment provider grasps their hands over the patient's knee with forearms controlling the leg.

Direction of Movement: Flexion, adduction and internal rotation

Grade of Movement: III and IV.

Figure F

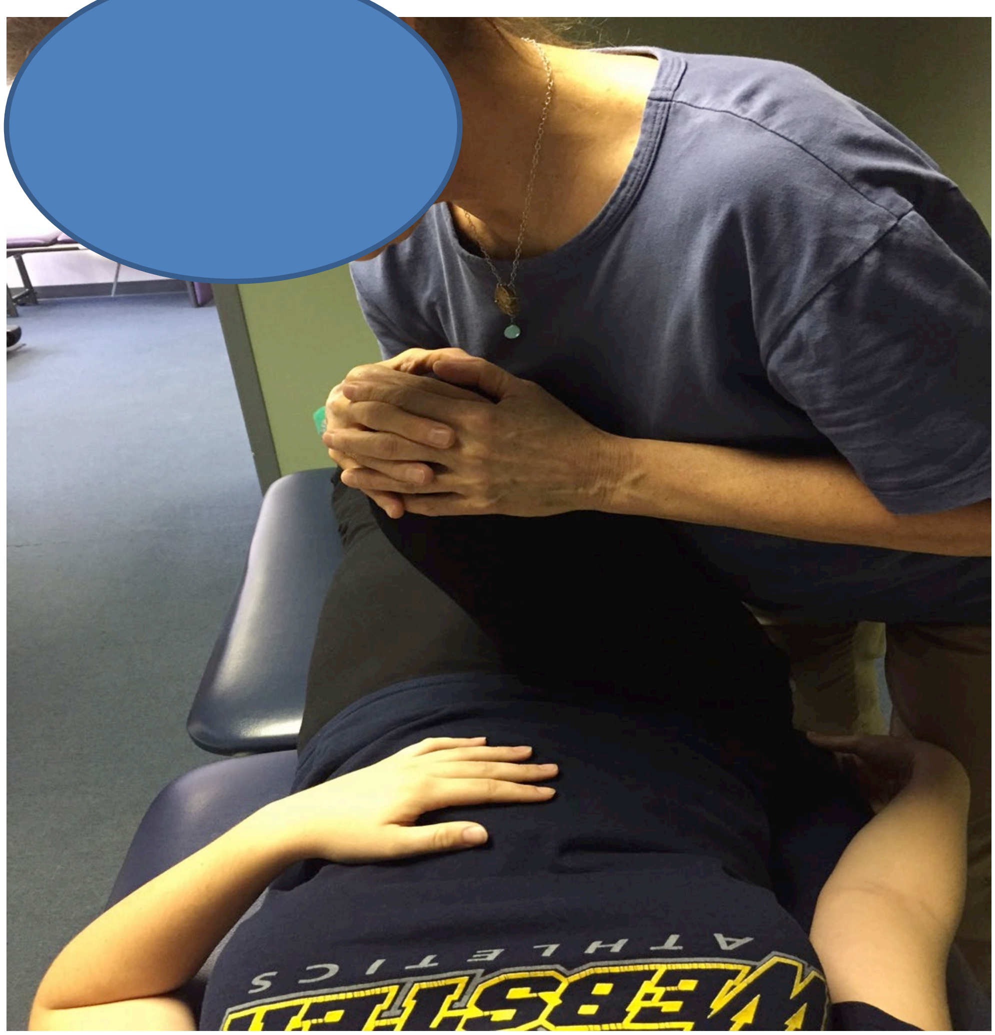

6. **Anterior Glide**

Uses: Decrease pain, Increase joint play, increase hip extension and/or external rotation

Comparable Sign: Painful or limited hip extension and/or external rotation.

Patient Position (Figure G): Prone with a pillow under the pelvis so the hips are flexed an appropriate amount for the patient. The treatment provider may include the addition of varying degrees of hip abduction, flexion and external rotation (Figure H).

Treatment Provider's Position and Handhold:

Stabilizing hand: distal aspect of femur just superior to patella.

Moving hand: heel of hand at posterior aspect of proximal.

Direction of Movement: anterior glide.

Grade of Movement: I-IV

Figure G

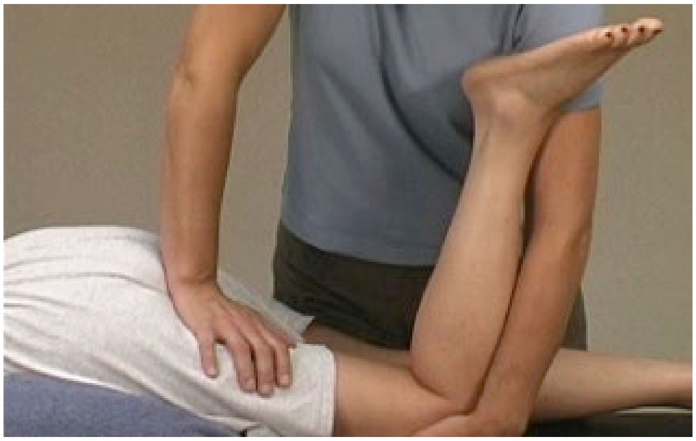

Figure H

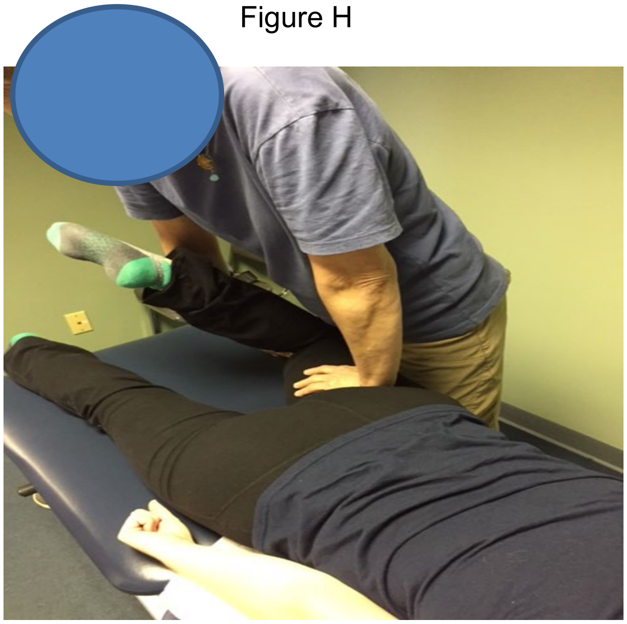

7. Internal Rotation in Supine

Uses: Decrease pain, increase internal rotation.

Comparable sign: Painful or limited hip internal rotation.

Patient Position: Patient lies supine with hip flexed. (Figure I)

Treatment Provider's Position and Handhold:

Stabilizing hand is on the knee/thigh.

Moving hand holds the patients ankle or lower leg.

Direction of movement: Internal rotation.

Grade of Movement: I-IV

Grade I: This can be performed with a pillow under the knee to put the hip in mild flexion. The treatment provider holds the patients knee with both hands and performs internal rotation of the femur, short of R1.

Grade II: This can be performed in the lower degrees for flexion, approximately 30° to 70°. If the leg is resting on a pillow, the same handhold is used over the knee.

If the leg is in higher degrees of flexion, the treatment provider will hold the lower leg and perform internal rotation.

Grade III and IV's: These can be performed in any position the P.T desires. In lower degrees of hip flexion the treatment provider will use a handhold over the knee. In higher degrees of flexion, the treatment provider will hold the lower leg.

Figure I

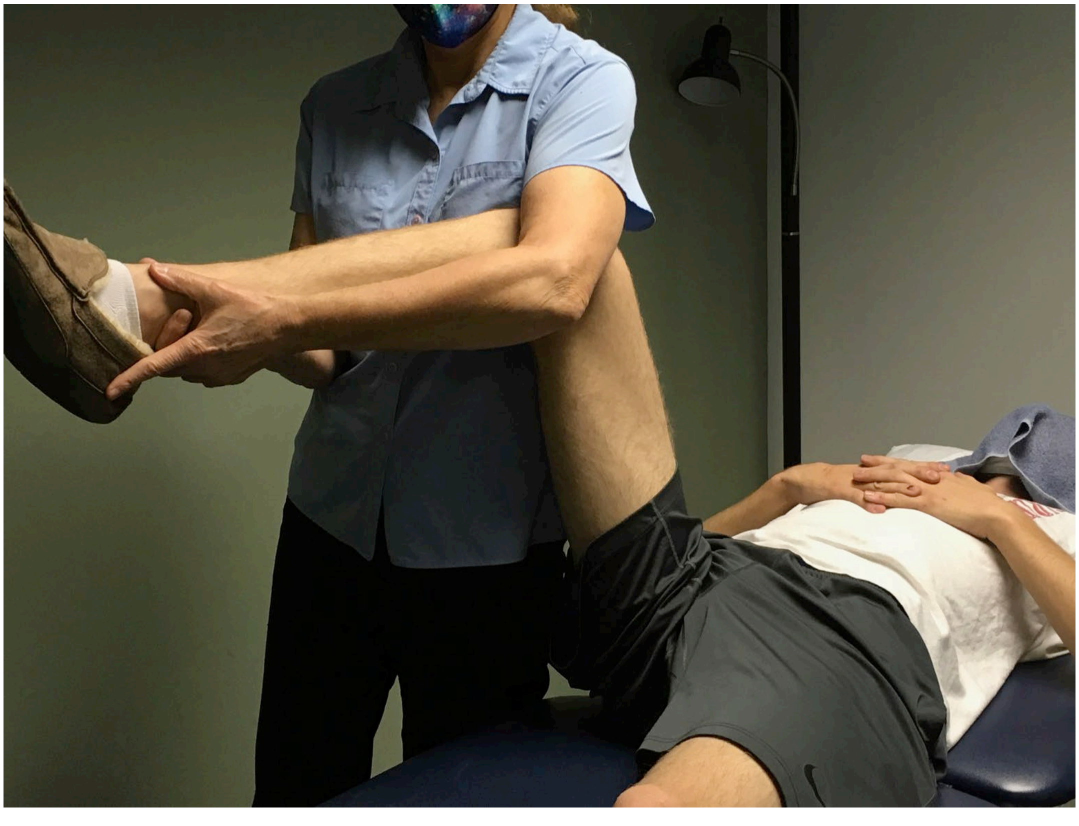

8. Internal Rotation in Prone

Uses: Decrease pain, increase internal rotation and/or extension

Comparable Sign: Painful or limited hip internal rotation or extension

Patient Position: (Figure J) The patient is prone with the knee flexed to 90° with the hip in neutral.

Treatment Provider's Position and Handhold:

Stabilizing hand: Provides an anterior force to the contralateral pelvis to prevent pelvis motion.

Moving hand: Grasp the anterior ankle.

Direction of Movement: Internal rotation.

Grade of Movement: I-IV

Figure J

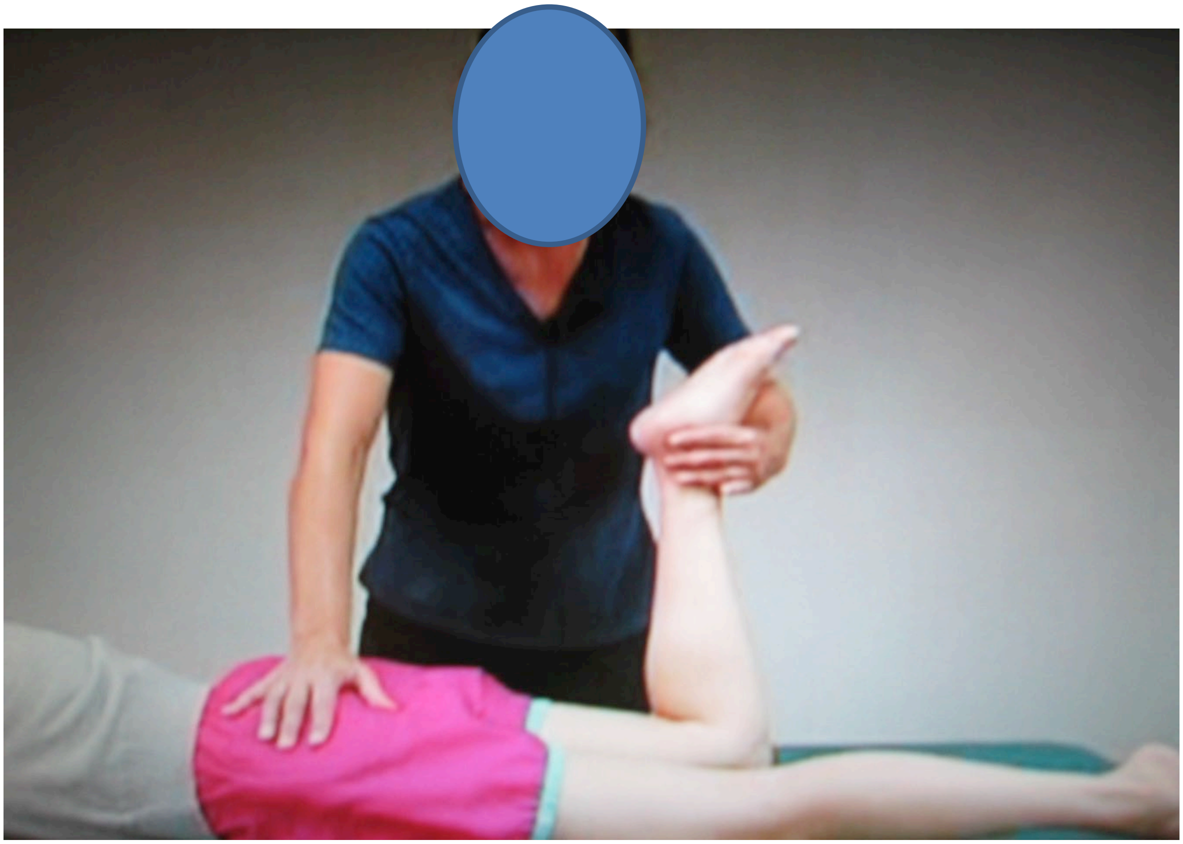

9. External Rotation in Supine

Uses: Decrease pain, increase external rotation and abduction

Comparable Sign: External rotation

Patient Position: (Figure K) Supine with pillow under the knee or higher degrees of hip flexion.

Treatment Provider's Position and Handhold:

In lower degrees of hip flexion: treatment provider's hands hold distal tibia and proximal tibia anteriorly.

In higher degrees of hip flexion: treatment provider's hands hold lower leg with hands cupping distal tibia.

Direction of Movement: External rotation

Grade of Movement: Grades I-IV

Figure K

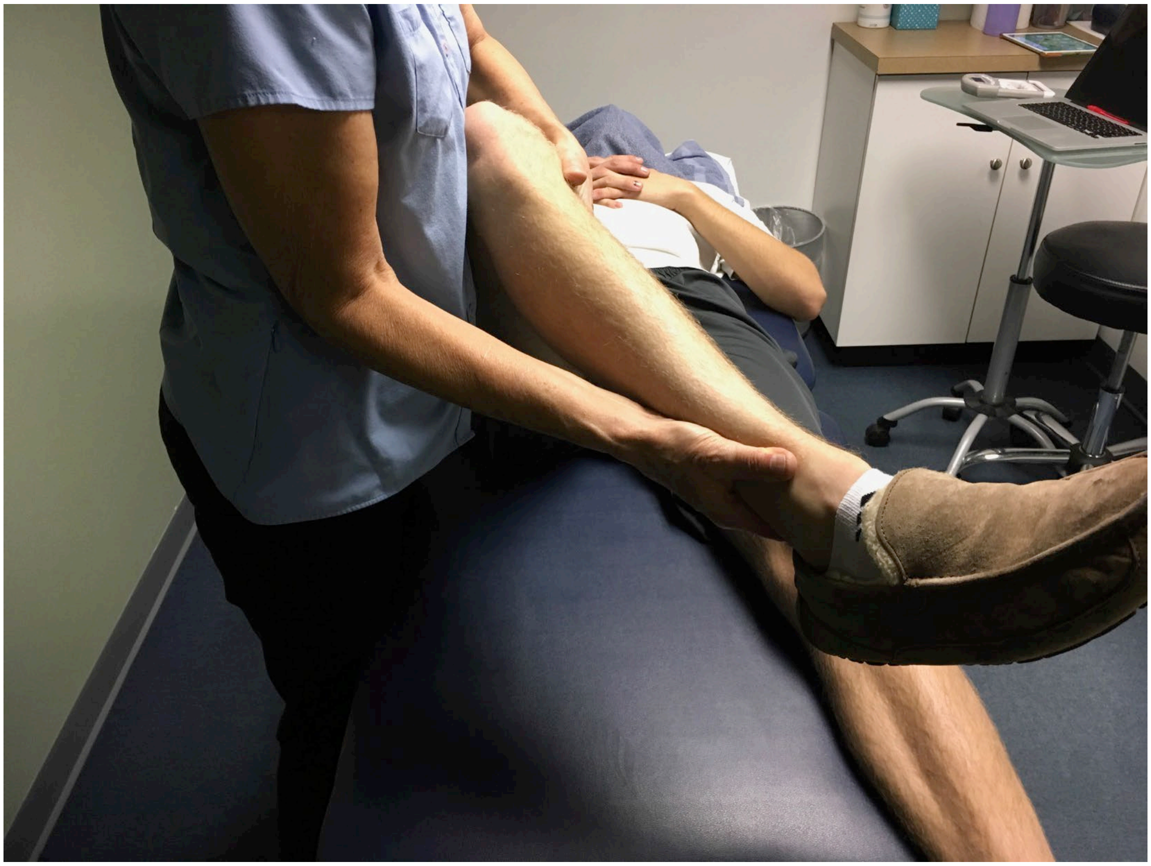

10. External Rotation in Prone

Uses: Increase External rotation, abduction and extension.

Comparable Sign: External rotation

Patient Position: (Figure L) Prone. Patient may need a pillow under the trunk depending on the amount of hip extension that is tolerable.

Treatment Provider's Position and Handhold:

Stabilizing hand: Posterior ipsilateral hip

Moving hand: lower leg above the ankle.

Direction of Movement: External Rotation

Grade of Movement: Grade I-IV

Figure L

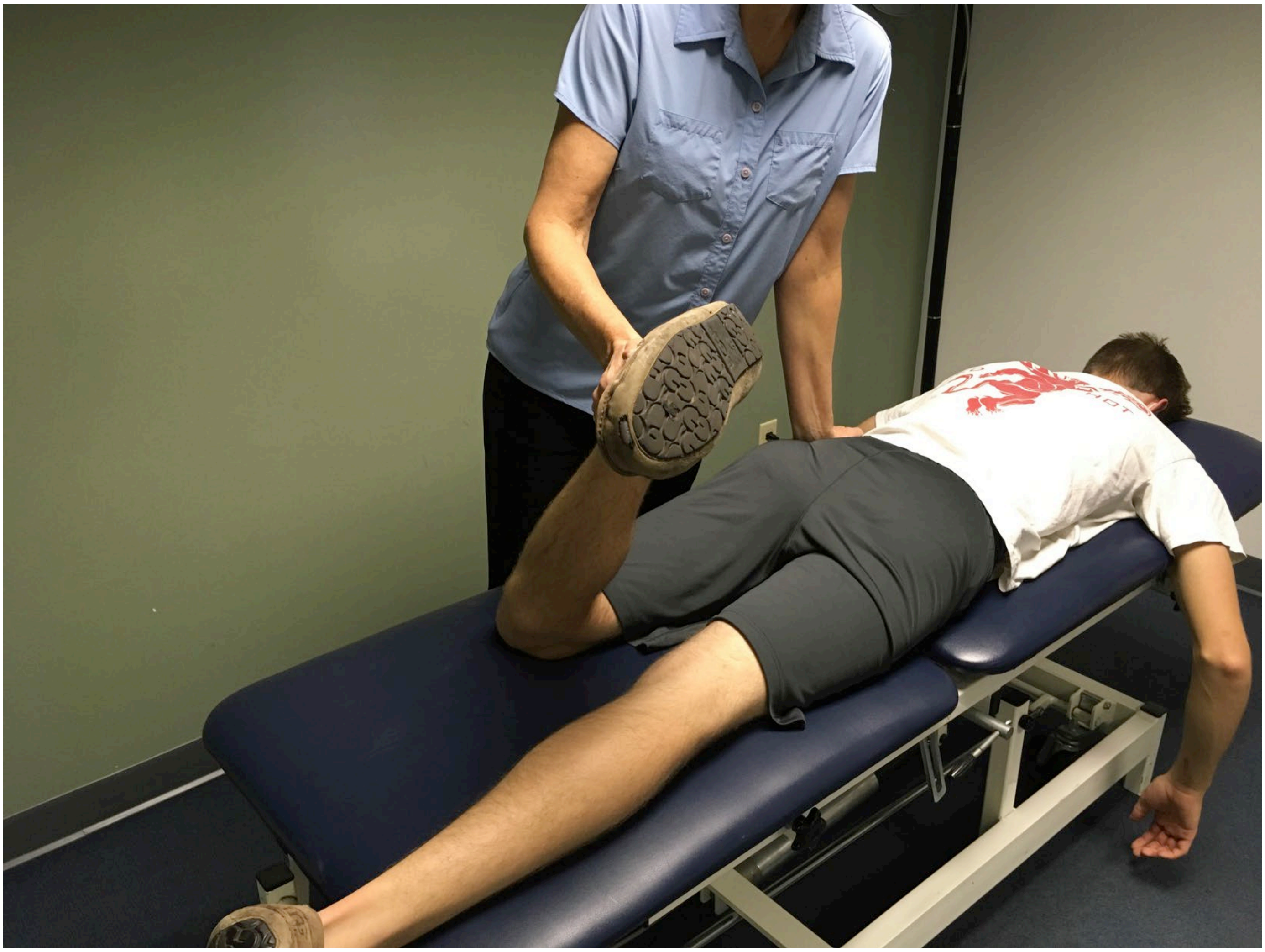

11. Adduction

Uses: Increase Adduction

Comparable Sign: Adduction

Patient Position: (Figure M) Supine or Prone

Treatment Provider's Position and Handhold:

Stabilizing hand: In lower degrees of hip flexion, one hand stabilizes the pelvis.

Moving hand: Distal femur or lower leg. In higher degrees of hip flexion, the treatment provider grasps over the patients flexed knee.

Direction of Movement: Adduction

Grade of Movement: Grade I-IV

Figure M

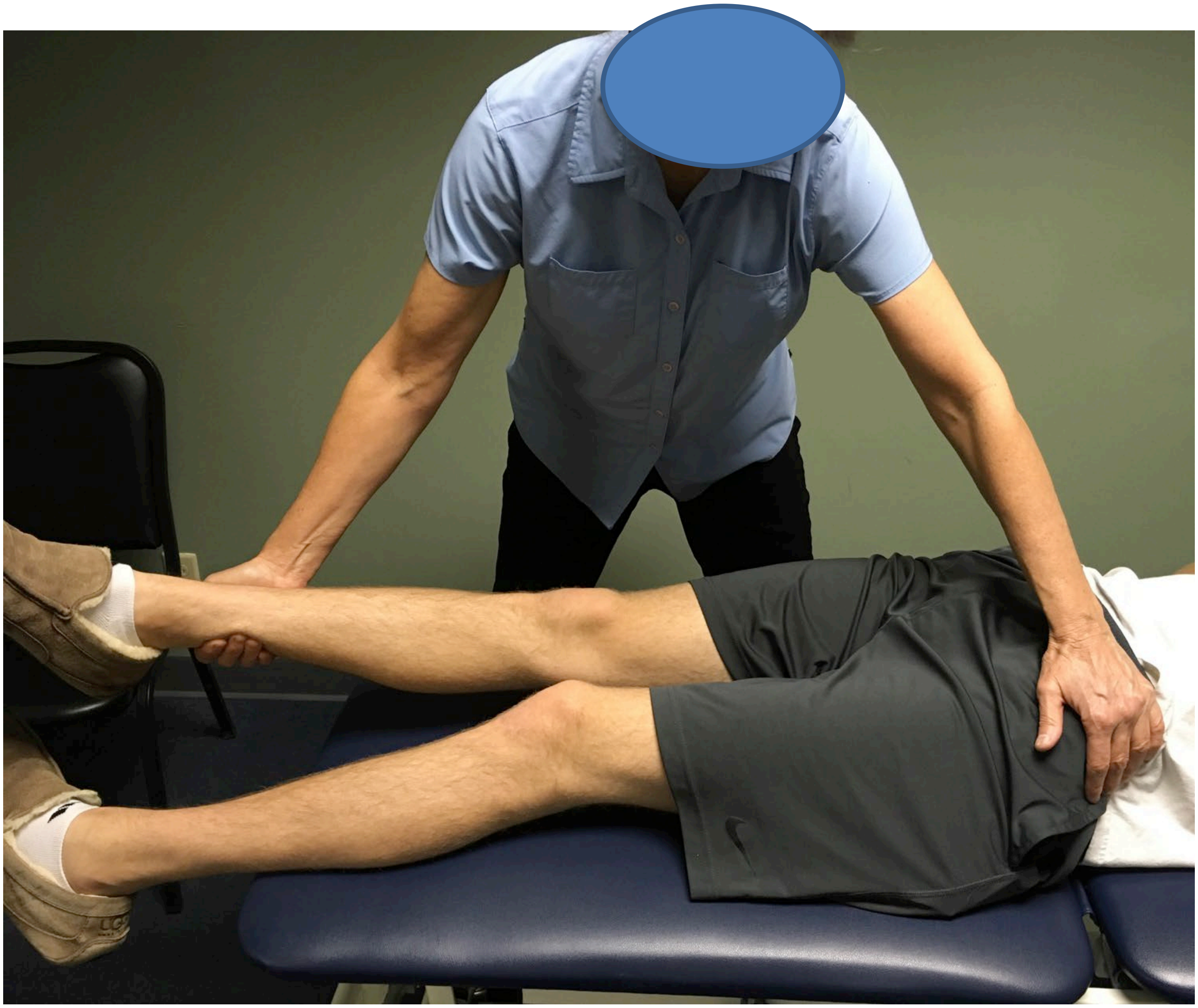

12. Abduction

Uses: Increase abduction

Comparable Sign: Abduction

Patient Position: (Figure N) Supine or prone

Treatment Provider's Position and Handhold:

Stabilizing hand: Lateral hip above the greater trochanter.

Moving hand: Distal thigh.

Direction of Movement: Abduction

Grade of Movement: Grade I-IV

Figure N

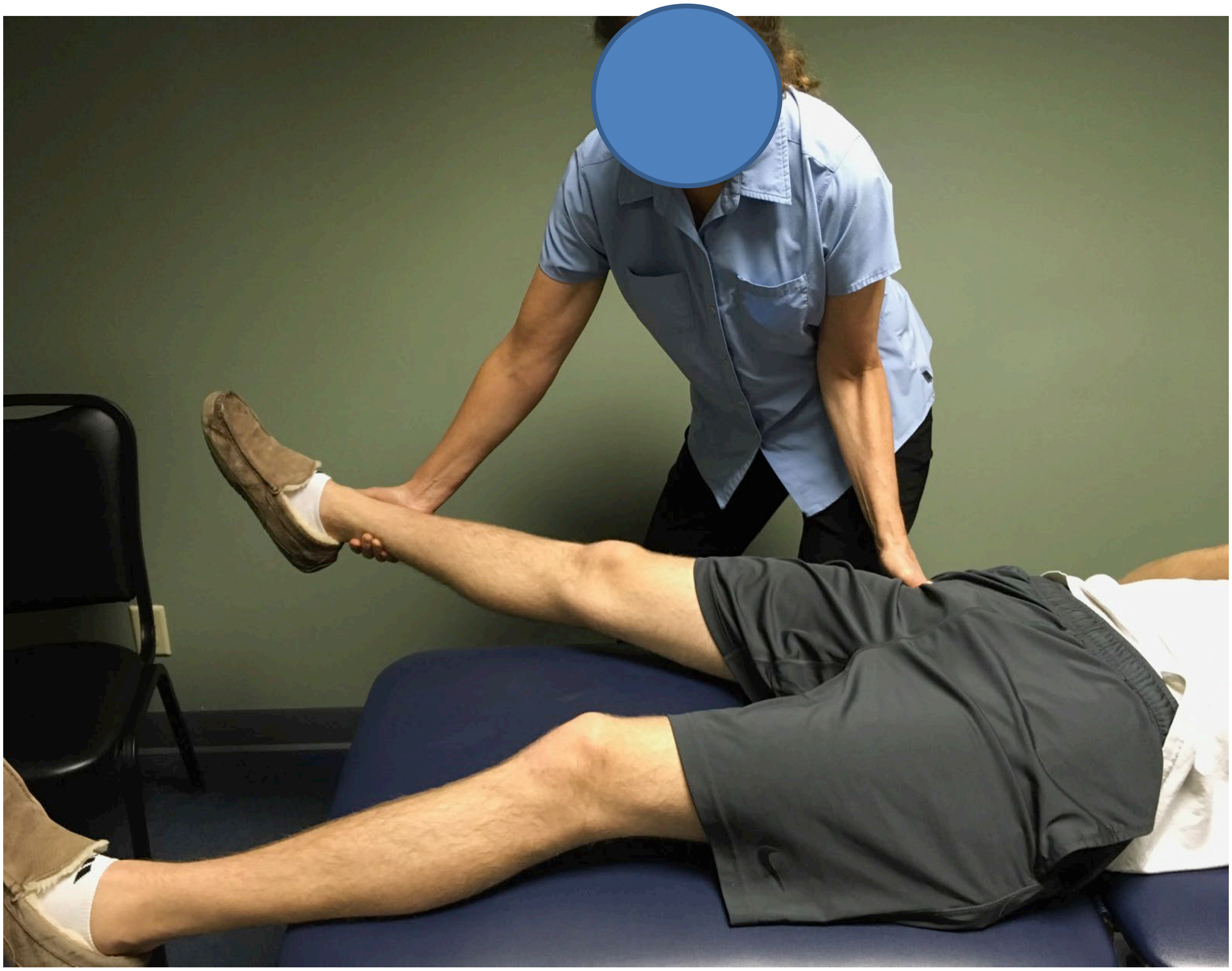

13. Extension

Uses: To increase Extension

Comparable Sign: Extension

Patient Position: (Figure O) Prone. May need pillows under the trunk depending on the amount of hip extension that is tolerable.

Treatment Provider's Position and Handhold:

Stabilizing hand: Posterior hip.

Moving hand: Distal anterior thigh above or on the knee.

Direction of Movement: Extension

Grade of Movement: Grade I-IV

Figure O

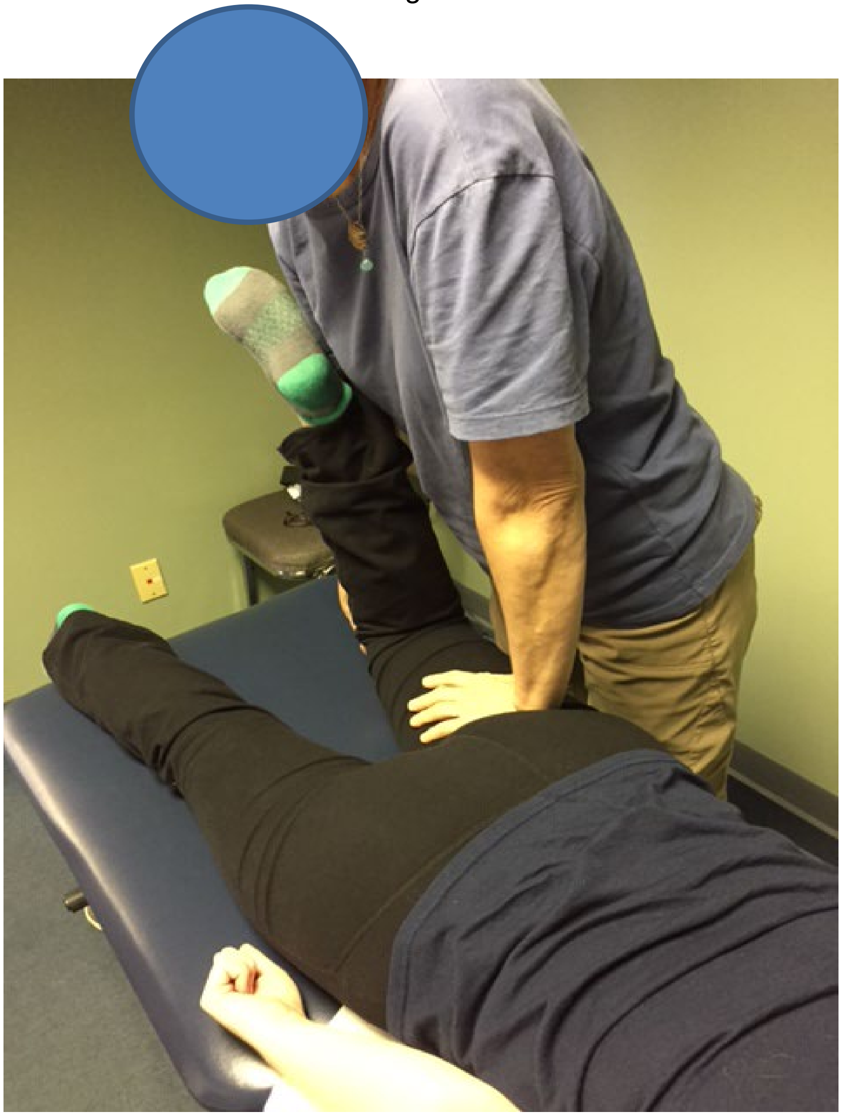

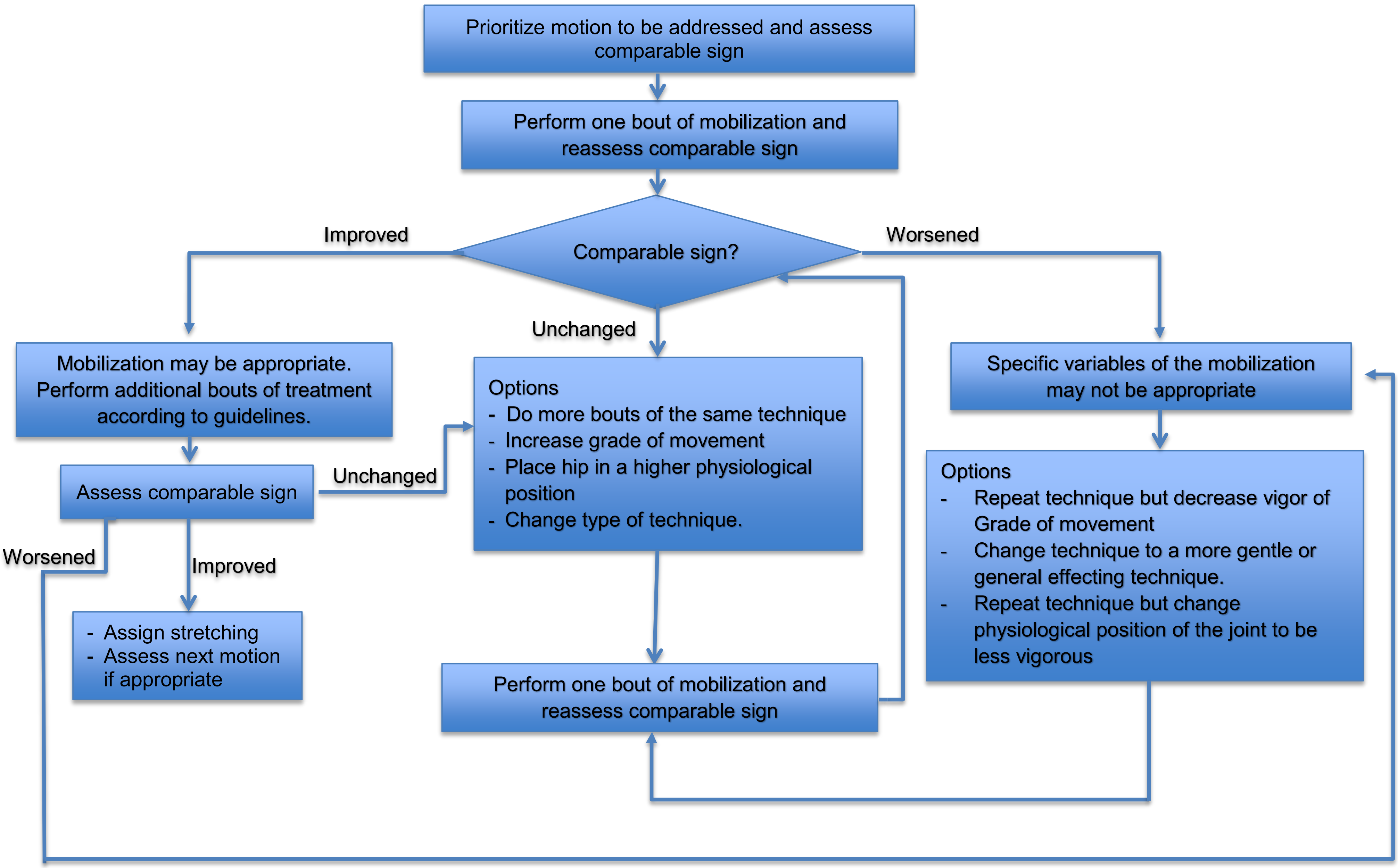

Figure 1. Decision tree demonstrating treatment providers' assessment and decision-making procedures related to joint mobilization techniques.

Table 1. Treatment techniques and home stretching exercise per hip joint restrictions

**Impaired Hip Motion**	**Therapeutic Manual Techniques: Accessory and Physiological motions**	**Therapeutic Stretches to be performed as a home program.**
Flexion	• Flexion<br>• Caudal glide<br>• Posterior glide<br>• Mobilization with movement<br>• Combinations of flexion, adduction and Internal rotation	• Hamstring stretch<br>• Two joint hip flexor stretch for flexion<br>• Figure 4 stretch in supine<br>• Single knee to chest<br>• Quadruped rocking back
External Rotation	• External rotation in supine<br>• External rotation in prone<br>• Caudal glide<br>• Anterior glide in prone	• Figure 4 stretch in supine<br>• External rotation in prone<br>• Figure 4 stretch in sitting<br>• Standing hip abduction stretch<br>• External rotation in prone
Internal Rotation	• Internal rotation in Supine<br>• Internal rotation in Prone<br>• Caudal glide<br>• Lateral glide<br>• Combinations of flexion, adduction and internal rotation	• Internal rotation in prone<br>• Two joint hip flexor emphasizing internal rotation with flexion
Adduction	• Adduction<br>• Caudal glide<br>• Lateral glide<br>• Posterior glide<br>• Combinations of flexion, adduction and internal rotation	• Two joint hip flexor emphasizing adduction with flexion<br>• Prone hip internal rotation stretch
Abduction	• Abduction<br>• Caudal glide	• Standing hip abduction stretch<br>• Figure 4 stretch in sitting or supine<br>• Standing abduction stretch<br>• Hook lying with bilateral hip abduction/external rotation
Extension	• Extension in prone<br>• Caudal glide<br>• Anterior glide in prone<br>• Internal Rotation in prone	• Two joint hip flexor stretch<br>• Hip extension in kneeling or standing<br>• Single heel slide from hook lying
FABER	• Combinations of flexion, abduction and external rotation<br>• Anterior glide	• Single knee to chest<br>• Figure Four stretch sitting or supine<br>• Hip abduction stretch in standing<br>• Prone hip external rotation stretch<br>• Hamstring stretch

FADIR	• Combinations of flexion, adduction and internal rotation<br>• Caudal glide<br>• Posterior glide<br>• Lateral glide<br>• Mobilization with movement	• Single knee to chest<br>• Prone hip external rotation stretch<br>• Quadruped rocking<br>• Two joint hip flexor stretch

Patient Education
Joint Mobilization

ID: ___ ___ ___

Visit #: ___

Date: ___ /___/ ___

Hip joint pain

- Hip joint pain may be due to one or more of the following:
  - Trauma, such as an accident or a fall
  - Related to participating in sporting activities such as running, soccer, hockey and others.
- Factors that may also contribute to why you have hip joint include
  - Bony abnormalities of the femur or pelvis that affect the amount of motion at your hip.
  - Hip muscle tightness or decreased joint flexibility
  - Participation in sports that require twisting, cutting or running
  - Nerve Sensitization – When pain lasts longer than expected – the brain and nervous system get more sensitive, or go on "high alert." These changes make your nerves more efficient at telling your brain about what is going on in your body tissues (i.e. your hip). This means small signals from the hip that should not normally cause pain may be enough to trigger a pain response.

Components of your treatment

As a participant in this study, your treatment will include a comprehensive regimen of activities to ensure adequate attention to all of the components of your impairment / problem. In the 10 P.T. sessions, you will receive the following:

- Education regarding activity modification and pain control.
- Hands-on manual therapy techniques
- Flexibility exercises as a home exercise program

Overall Goals of treatment

- Reduce pain by decreasing sensitization
- Increase pain-free range of motion of the hip joint and surrounding soft tissues
- Stretching muscles of the lower extremities
- Modifying of symptom-aggravating activities

Education

- Pain management
  - Ice
  - Rest
- Pain-relieving medications
- Limit time in excessively hip flexed positions
- Activity/ADL modification by reducing time spent participating in activities that aggravate symptoms
- Considerations regarding post-treatment soreness location and duration

Manual therapy treatment

You will be receiving hands-on techniques during your treatment sessions. These techniques may cause discomfort during and / or after the technique. This is a normal response to passive treatment. The symptoms should not be severe. Your therapist will be asking you about your symptoms before, during and after these techniques are performed at each visit. It is important for you to focus on the symptoms so you can relate this information to your therapist during your visit and at subsequent visits. Increased soreness after therapy

should resolve within a day. If you have significant increased soreness after therapy, you may benefit from the following:

- Increase the time of resting your hip in the most comfortable position you can find. Stay in this position frequently through the day.
- Decrease the amount of time you spend in weight bearing activities that tend to worsen your symptoms.
- Discontinue stretching exercises that may increase your symptoms.
- Use ice or heat on the painful area for 10 minutes, 3-4 times a day as needed. Be sure to monitor your skin to avoid burns.

Continue to adhere to these tips until your pain returns to normal baseline symptoms. Then you may resume stretching exercises and normal activity as prescribed by your therapist. If the increased symptoms linger for more than 48 hours, call your therapist for advice.

Exercises

You will be receiving exercises to improve your flexibility. These exercises may result in muscle soreness, however the exercises should not increase your hip joint pain. If any exercise increases your hip joint pain, try the following:

1. Review exercise handout to be sure you are performing correctly. If you were performing incorrectly, correct performance. If pain is decreased with corrected performance, continue with exercise as instructed.
2. If you are performing correctly, and you are
    a. experiencing pain at the very end of the motion, reduce the range of motion that you are performing. Continue to perform the exercise with the limited motion until you see your physical therapist.
    b. experiencing increased pain after a certain number of repetitions, perform only the number you can perform without increased pain. Continue to perform the exercise at this reduced number of repetitions until you see your physical therapist.
3. If pain is not alleviated with the above corrections, discontinue the aggravating exercise until you see your physical therapist again.

Activity modifications

1. If your pain is related to a fitness or sporting activity, modification of this activity will allow for your injury to heal. You may modify your activity by reducing the frequency, the intensity or the duration of the activity.

<u>RETURN TO FITNESS</u>

It is important to participate in physical activity to maintain or improve your overall health. Because of your injury, you may need to refrain from or limit your activities to allow for healing. Once you and your physical therapist have determined that it is time to return to your fitness routine, your physical therapist will assist you in designing a program to gradually return to your activity. Below is an example of a return to running program. Please discuss with your physical therapist prior to initiating the running program or other physical activity.

Example program to progress running:

Basic instruction for progressing program

Patient Education
Joint Mobilization

ID: ___ ___ ___

Visit #: ___

Date: ___ /___/ ___

- stay at each level for 2 runs minimum
- if pain is experienced during a level, go down to the previous level

____Level 1: 15 seconds run/45 seconds walk x 4

____Level 2: 30 seconds run/1 minute walk x 4

____Level 3: 1 minute run/1-2 minutes walk x 4 building up to 10 (4-10 minutes total of running)

____Level 4: 2 minutes running/1 minute walk x 5 building up to 12 (10-24 minutes total of running)

____Level 5: 3 minutes running/1 minute walk x 8 (24 minutes running)

____Level 6: 4 minutes running/1 minute walk x 6 (24 minutes running)

____Level 7: 6 minutes running/1 minute walk x 4 (24 minutes running)

____Level 8: 8 minutes running/1 minute walk x 3 (24 minutes running)

____Level 9: 12 minutes running/1 minute walk x 2 (24 minutes running)

____Level 10: 15-20 minute solid run

____Level 11: Build by 1-3 minutes per run until you are at your goal distance

Flexibility – Doorway Hamstring Stretch

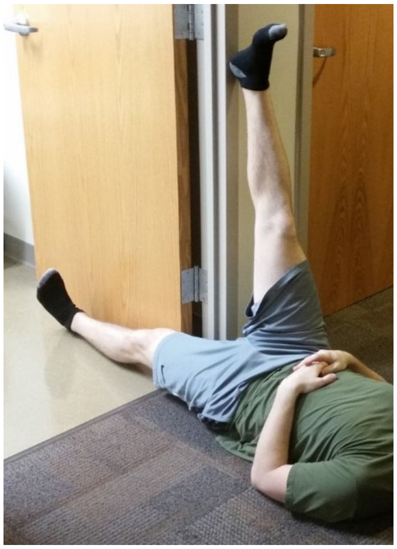

Purpose: To improve hip flexion motion.

1. Lie on your back near a doorway
2. Keeping your knee straight, bring your leg up along the doorframe.
3. You should feel the stretch in the back of your thigh.
4. Make sure your lower leg stays flat on the floor.
5. Stretch to the point of feeling tightness or slight discomfort.

Hold 30 secs, 2-4 reps (can adjust hold and reps, but should accumulate 60 sec of stretch total), perform 5x/week

Flexibility – Standing Hamstring Stretch

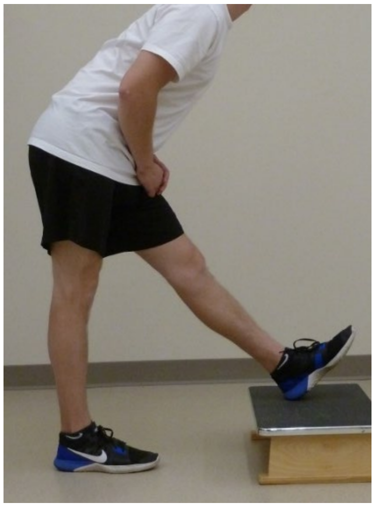

Purpose: To improve hip flexion motion.

1. Place the heel of the leg to be stretched on a short step.
2. Keeping your knee straight and back straight, lean forward at the hip
3. You should feel the stretch in the back of your thigh.
4. Stretch to the point of feeling tightness or slight discomfort.

Hold 30 secs, 2-4 reps/side (can adjust hold and reps, but should accumulate 60 sec of stretch total), perform 5x/week

Flexibility – Supine Hamstring Stretch

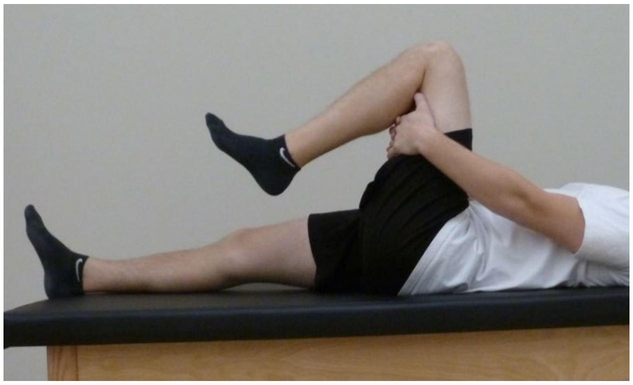

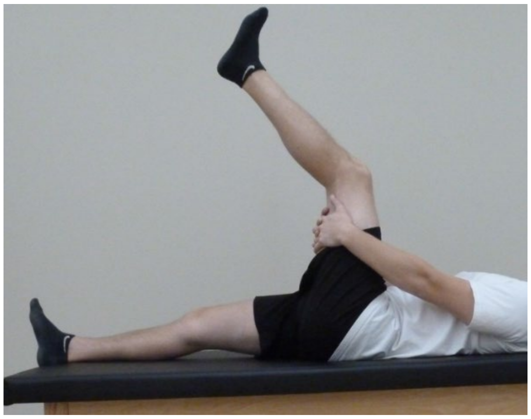

Purpose: To improve hip flexion motion.

1. Lie on your back
2. Bring the thigh of the leg to be stretched towards your chest and hold with your hands.
3. Keeping your back straight, straighten the knee.
4. You should feel the stretch in the back of your thigh.
5. Stretch to the point of feeling tightness or slight discomfort.

Hold 30 secs, 2-4 reps/side (can adjust hold and reps, but should accumulate 60 sec of stretch total), perform 5x/week

Flexibility – Single Knee to Chest Stretch

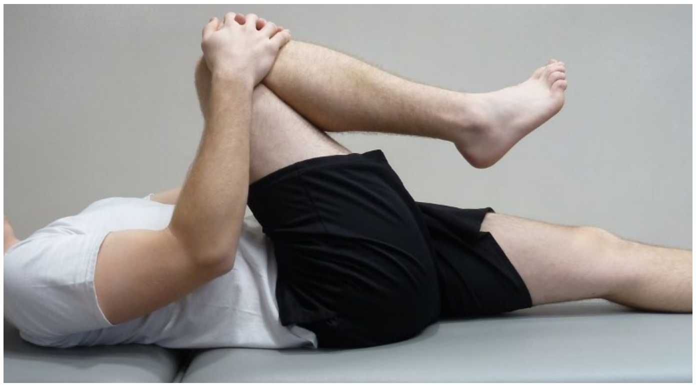

Purpose: To improve hip flexion.

1. Lie on table or firm bed with legs straight.
2. Bring involved knee towards the chest.
3. Feel stretch in the back of the hip and buttock.
4. Stretch to the point of feeling tightness or slight discomfort.

Hold 30 secs, 2-4 reps (can adjust hold and reps, but should accumulate 60 sec of stretch total), perform 5x/week

Flexibility – Quadruped Rocking Back

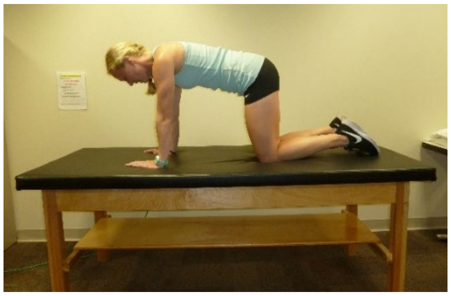
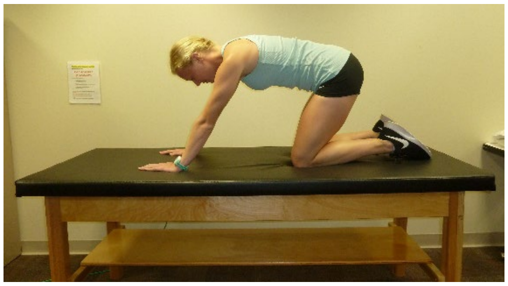
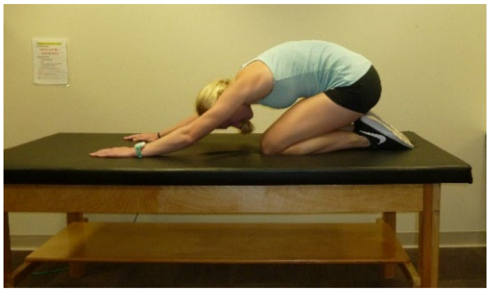

Purpose: To improve hip flexion motion.

1. Position yourself on your hands and knees.
2. Keeping your hands still, rock hips backwards towards your heels.
3. Feel stretch in the back of your hips and buttocks.
4. Stretch to the point of tightness or slight discomfort.
5. Resume starting position.

Hold 30 secs, 2-4 reps (can adjust hold and reps, but should accumulate 60 sec of stretch total), perform 5x/week

Flexibility – Figure Four Stretch in Sitting

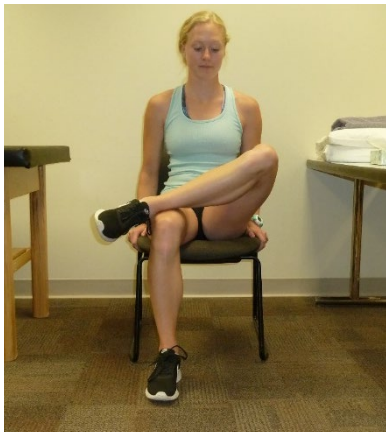

Purpose: To improve

___hip external rotation motion.
___hip abduction motion.

1. Sit in a chair.
2. Bring ankle of the leg to be stretched over to the other knee.
3. Allow gravity to stretch the bent leg.
4. Stretch to the point of tightness or discomfort.

Hold 30 secs, 2-4 reps (can adjust hold and reps, but should accumulate 60 sec of stretch total), perform 5x/week.

____OPTION: apply pressure to the bent knee towards the ground to increase the stretch.

Flexibility – Figure Four Stretch in Supine

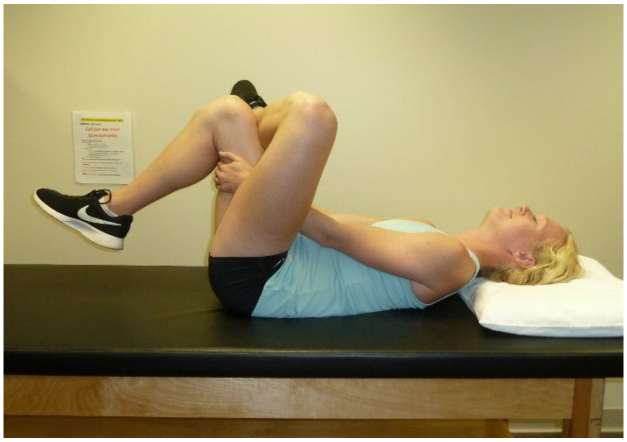

Purpose: To improve

___hip flexion motion.
___hip external rotation motion.
___hip abduction motion.

1. Lying on your back with knees bent.
2. Cross the ankle of the leg to be stretched over the other knee. Put your arms around the thigh as shown.
3. Bring your thigh towards your stomach. Feel the stretch in your buttock.
4. Stretch to the point of feeling tightness or slight discomfort.

Hold 30 secs, 2-4 reps (can adjust hold and reps, but should accumulate 60 sec of stretch total), perform 5x/week

Flexibility – External Rotation in Prone

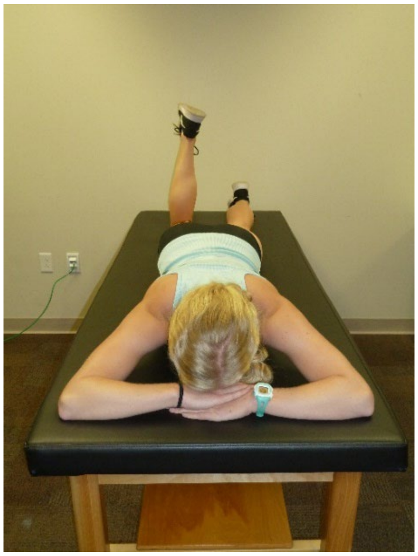

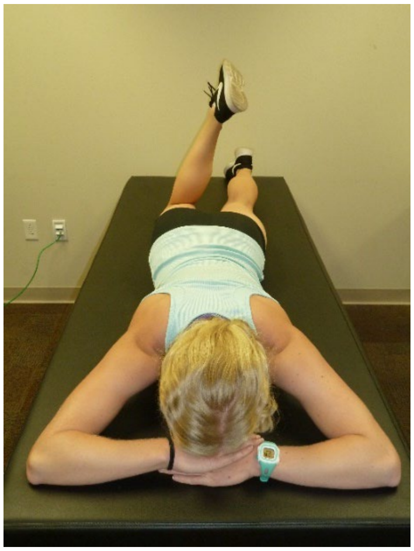

Purpose: To improve
___hip external rotation motion.

1. Lie on your belly.
2. Bend the knee of the hip to be stretched as shown.
3. Rotate the leg in towards the opposite leg.
4. You should feel the stretch in the outside of your lateral hip.
5. Stretch to the point of feeling tightness or slight discomfort.

Hold 30 secs, 2-4 reps (can adjust hold and reps, but should accumulate 60 sec of stretch total), perform 5x/week

____OPTION: place a pillow under your hips

Flexibility – Internal Rotation in Prone

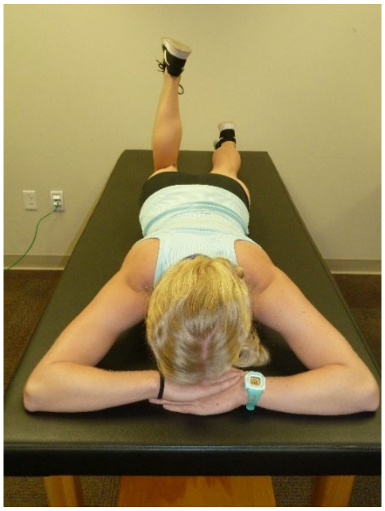
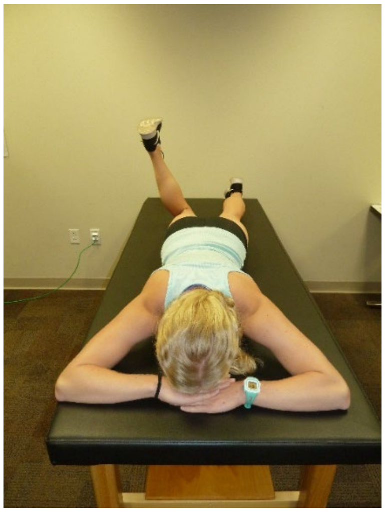

Purpose: To improve

___hip internal rotation motion.
___hip adduction motion.

1. Lie on your belly.
2. Bend the knee of the hip to be stretched as shown.
3. Rotate the leg away from the opposite leg.
4. You should feel the stretch in the buttocks.
5. Stretch to the point of feeling tightness or slight discomfort.

Hold 30 secs, 2-4 reps (can adjust hold and reps, but should accumulate 60 sec of stretch total), perform 5x/week

____OPTION: place a pillow under your hips

Flexibility – Hooklying with Bilateral Hip Abduction/External Rotation

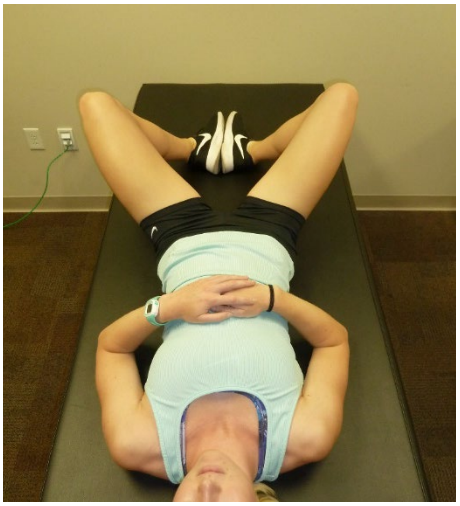

Purpose: To improve hip adduction motion.

1. Lie on back with knees bent.
2. Allow your knees to fall out to the sides. Feel the stretch along your inner thighs.
3. Stretch to the point of feeling tightness or slight discomfort.
4. Return to the starting position.

Hold 30 secs, 2-4 reps (can adjust hold and reps, but should accumulate 60 sec of stretch total), 5x/week

Flexibility – Standing Abduction Stretch

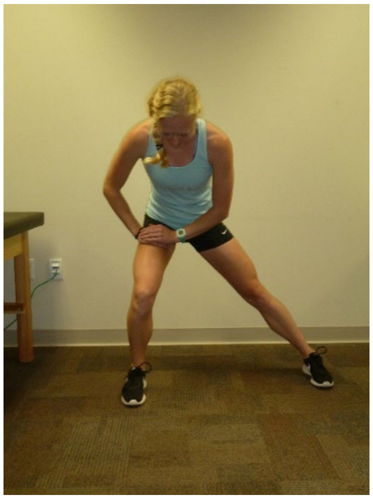

Purpose: To improve hip abduction motion.

1. Standing with leg to be stretched out and away and the opposite extremity slightly bent as shown.
2. Place your hands on the bent knee and lean away from the leg to be stretched.
3. Bring your chest towards your bent knee. Feel the stretch in your inner thigh of the straight leg.
4. Stretch to the point of feeling tightness or slight discomfort.

Hold 30 secs, 2-4 reps (can adjust hold and reps, but should accumulate 60 sec of stretch total), perform 5x/week

Flexibility – Single Heel from Hooklying

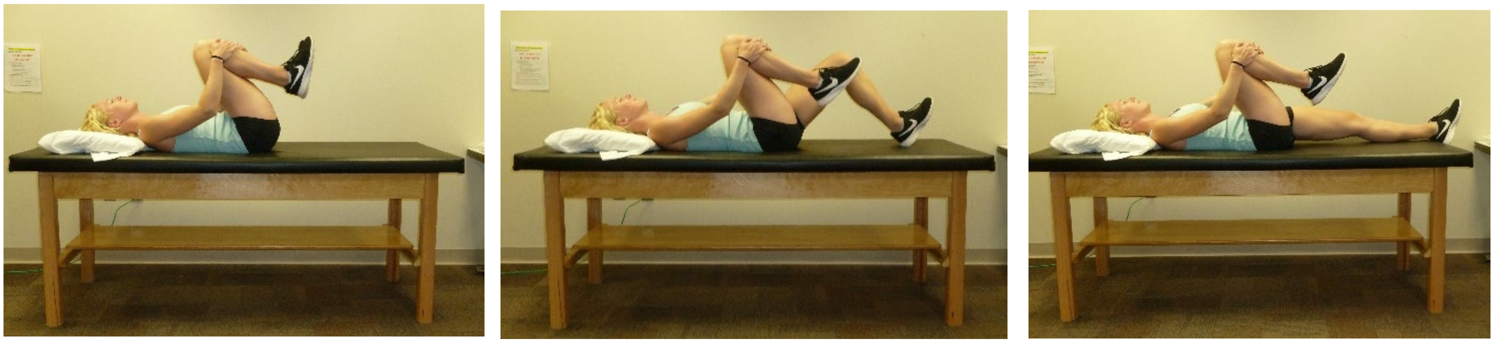

Purpose: To improve hip extension.

1. Begin with both knees bent and feet flat on the surface.
2. Bring your knees one at a time towards your chest to make the low back flat on the surface.
3. Allow the involved leg to stretch towards the surface.
4. Feel the stretch in the front of the hip and/or thigh.
5. Stretch to the point of feeling tightness or slight discomfort.

Hold 30 secs, 2-4 reps (can adjust hold and reps, but should accumulate 60 sec of stretch total), perform 5x/week

____ OPTION: Place a pillow under your involved thigh. Lower your thigh to the pillow and allow your leg to relax

Flexibility – Hip Extension in Kneeling

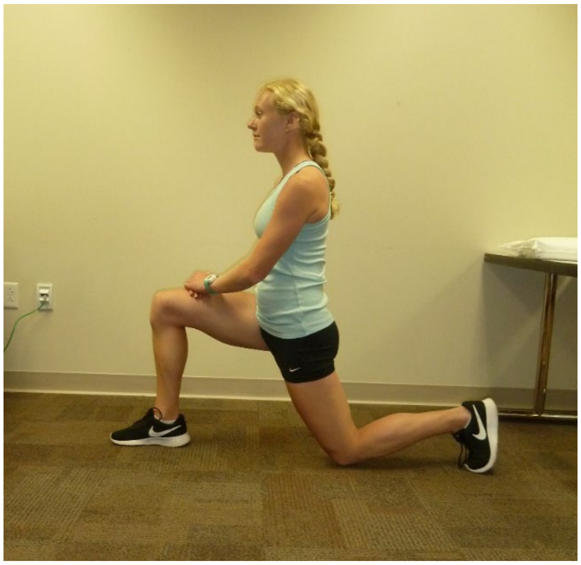

Purpose: To improve hip extension motion.

1. Get into the kneeling position with one leg in front of the other.
2. Keeping back straight, lean weight forward towards front leg.
3. Stretch to the point of feeling tightness or slight discomfort.

Hold 30 secs, 2-4 reps/side (can adjust hold and reps, but should accumulate 60 sec of stretch total), perform 5x/week

Flexibility –Hip Extension in Standing

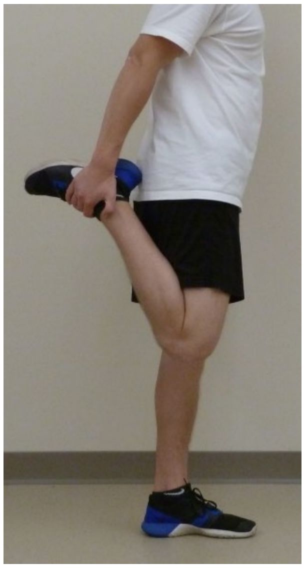

Purpose: To improve hip extension motion.

1. Get into the standing position.
2. Keeping back straight, bend your knee and pull your foot toward your buttocks as shown in the picture.
3. Stretch to the point of feeling tightness or slight discomfort.

Hold 30 secs, 2-4 reps/side (can adjust hold and reps, but should accumulate 60 sec of stretch total), perform 5x/week

Flexibility – Two Joint Hip Flexor Stretch

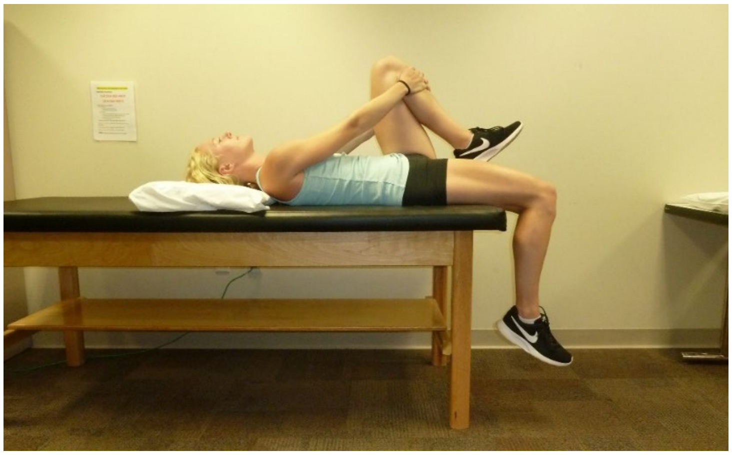

Purpose: To improve hip motion

1. Lie on a table or firm bed as shown.
2. Place your hands behind involved/ uninvolved knee and pull it towards your chest to keep the low back flat on the surface.
3. Allow the other leg to stretch over the edge and to the floor.
4. You may feel the stretch on the front of the hip/thigh lying over the edge or in the buttocks of the bent hip.
5. Stretch to the point of feeling tightness or slight discomfort.

Hold 30 secs, 2-4 reps (can adjust hold and reps, but should accumulate 60 sec of stretch total), perform 5x/week

___hip flexion motion
___hip extension motion

References

1. Harris-Hayes M, Steger-May K, Bove AM, et al. Movement pattern training compared with standard strengthening and flexibility among patients with hip-related groin pain: results of a pilot multicentre randomised clinical trial. *BMJ Open Sport Exer Med.* 2020;6:e000707. PMCID: PMC7254120.
2. Harris-Hayes M, Czuppon S, Van Dillen LR, et al. Movement-pattern training to improve function in people with chronic hip joint pain: a feasibility randomized clinical trial. *J Orthop Sports Phys Ther.* 2016;46:452-461. PMCID: PMC4889512.
3. Deyle GD, Henderson NE, Matekel RL, Ryder MG, Garber MB, Allison SC. Effectiveness of manual physical therapy and exercise in osteoarthritis of the knee. A randomized, controlled trial. *Ann Intern Med.* 2000;132:173-181.
4. Wright AA, Hegedus EJ, Taylor JB, Dischiavi SL, Stubbs AJ. Non-operative management of femoroacetabular impingement: A prospective, randomized controlled clinical trial pilot study. *J Sci Med Sport.* 2016;19:716-721.
5. Deyle GD, Gill NW, Rhon DI, et al. A multicenter randomised, 1-year comparative effectiveness, parallel-group trial protocol of a physical therapy approach compared to corticosteroid injection on pain and function related to knee osteoarthritis (PTA Trial). *BMJ open.* 2016;6:e010528. PMC4823390.
6. Maitland GD. *Peripheral Manipulation.* Vol 2nd Great Britain: Butterworth & Co.; 1977.
7. Hartman LS. *Handbook of Osteopathic Technique.* 3rd ed: Springer US; 1996.
8. Wise CH. The Paris Approach. In: *Orthopaedic Manual Physical Therapy: From Art to Evidence.* Vol 1st. Philadelphia, PA: F.A. Davis Company; 2015.
9. Abbott JH, Robertson MC, Chapple C, et al. Manual therapy, exercise therapy, or both, in addition to usual care, for osteoarthritis of the hip or knee: a randomized controlled trial. *Osteo Cart.* 2013;21:525-534.
10. Mansell NS, Rhon DI, Marchant BG, Slevin JM, Meyer JL. Two-year outcomes after arthroscopic surgery compared to physical therapy for femoracetabular impingement: A protocol for a randomized clinical trial. *BMC Musculoskelet Disord.* 2016;17:60. PMCID: PMC4743428.
11. Hengeveld E, Backs K. *Maitland's Peripheral Manipulation: Management of Neuromusculoskeletal Disorders.* Vol 2. 5th ed: Churchill Livingstone; 2013.
12. Priya i. Effect of Maitland spinal mobilization therapy versus conventional therapy in lumbar spondylosis with radiculopathy. *Indian J Physiother Occupl Ther* 2013;7:177-183.
13. Dueñas L, Balasch-Bernat M, Aguilar-Rodríguez M, Struyf F, Meeus M, Lluch E. A manual therapy and home stretching program in patients with primary frozen shoulder contracture syndrome: a case series. *J Orthop Sports Phys Ther.* 2019;49:192-201.
14. Kumar A, Kumar S, Aggarwal A, Kumar R, Das PG. Effectiveness of Maitland techniques in idiopathic shoulder adhesive capsulitis. *ISRN Rehabilitation.* 2012;2012:710235.
15. Mahakul B, Singh H, Sahoo J, Samant S. Effectiveness of Maitland mobilization technique on pain and hand functions in the postoperative management of Colles fracture. *Int J Orthop Sci.* 2017;3:397-399.
16. Vermeulen HM, Rozing PM, Obermann WR, le Cessie S, Vliet Vlieland TP. Comparison of high-grade and low-grade mobilization techniques in the management of adhesive capsulitis of the shoulder: randomized controlled trial. *Phys Ther.* 2006;86:355-368.
17. Deyle GD, Allison SC, Matekel RL, et al. Physical therapy treatment effectiveness for osteoarthritis of the knee: a randomized comparison of supervised clinical exercise and manual therapy procedures versus a home exercise program. *Phys Ther.* 2005;85:1301-1317.
