# Supplementary material for: Comparison of Joint Mobilization and Movement Pattern Training for Patients With Hip-Related Groin Pain: A Pilot Randomized Clinical Trial
Source: Phys Ther. 2023 Aug 22;103(11):pzad111. doi: 10.1093/ptj/pzad111 (PMC10683042; doi:10.1093/ptj/pzad111)
Supplement: b_appendix_2_final_pzad111 [file b_appendix_2_final_pzad111.pdf]

Appendix 2: Movement Pattern Training Protocol

The content in this appendix has been modified and is provided with permission from Harris-Hayes et al. Movement pattern training compared with standard strengthening and flexibility among patients with hip-related groin pain: results of a pilot multicentre randomised clinical trial. *BMJ Open Sport Exer Med.* 2020;6:e000707.

The procedures used in this treatment arm have been described previously.[1-3] Resources used to develop the movement pattern training protocol included previous publications,[1-7] communications with physical therapists with expertise in movement pattern training, and feedback from the patients and physical therapists who participated in the previous trials.[1-3]

- Progression table for task-specific training
    - Handout used by the physical therapist to provide the general overview of the progression for task-specific training.
- Patient education
    - Handouts provided to each patient during their first treatment session.
- Patient-specific task instructions
    - Handouts with instructions for task-specific modifications that were provided to a patient if they reported that task as symptom-provoking.
- Exercise instructions
    - Handouts with exercise instructions that were provided to a patient as each exercise was prescribed.
- Protocol modifications
    - Summary of modifications made to the protocol published in Harris-Hayes et al. Movement pattern training compared with standard strengthening and flexibility among patients with hip-related groin pain: results of a pilot multicentre randomised clinical trial. *BMJ Open Sport Exer Med.* 2020;6:e000707.

Movement Pattern Training – Progression Table for Task-specific training					
**Task – Movements***	**Level 1**	**Level 2**	**Level 3**	**Level 4**	**Level 5**
1. Sit to Stand Squat	Slow lowering to seat w/o resistance	Slow lowering to seat with resistance	Slow lowering to seat (tap only) with resistance	Bilateral squat	Bilateral squat with resistance
2. Single leg activities	Weight shift	Single leg stance with hold	Single leg squat with hand support	Single leg squat	Single leg squat with resistance
3. Stair ambulation Goal: 8 inch minimum	Stairs with upper extremity support	Stairs w/o upper extremity support	Stairs with increased speed, no resistance	*Stairs with resistance	*Stairs progress resistance
				*For resistance, try theraband, however may modify by using weights or backpack	
4. Side stepping	Swing leg to side and tap (hip abduction)	Side step	Side step with resistance	Side step progress resistance	Side step with increase speed
5. Stance with movement	Stand on step, swing opp leg, large arc	Stand on step, swing opp leg, large arc fast	Stand on step, swing opp leg, large arc fast (compliant surface)	Stand on step, swing opp leg like run	Stand on step, swing opp leg like run (compliant surface)
**Task – Position**					
Standing	Participant will be provided instruction in at least one position task during the initial 2 visits. Each activity will be assessed during follow up visits, until the participant is independent in the task. To be independent in the task, the participant must verbalize and demonstrate proper performance. Once the participant is independent in a positioning task, they no longer need instruction, unless upon patient request.				
Sitting					
Sleeping					
**Patient-specific tasks†**	Tasks will be identified by the participant during their baseline assessment using the Patient Specific Functional Scale. Activities typically include work-related activities such as standing and sitting or fitness/sport activities. Tasks will be practiced and progressed in difficulty based on participant performance. See example of Return to Running program below. Similar concepts may be used for other tasks				

* **The goal of exercises 1-5**: To improve impaired movement pattern of the lower extremity during functional tasks. The primary movement impairments targeted during these exercises are movements of excessive hip adduction and internal rotation. Excessive hip extension should also be addressed. The treating physical therapist may use verbal cues, demonstration, mirrors or tactile cues to assist the patient.

***To assess for appropriate level at the initial visit (Ex 1-5):** Based on the clinical examination, the principle investigator will recommend a level to begin with. For each task, first observe the participant's natural movement pattern. Note any excessive movement impairments such as excessive hip adduction (medial collapse pattern or excessive pelvic drop) or hip and knee hyperextension.

- If impairment is present, educate the participant in the corrected movement pattern and determine if participant can perform the corrected task independently. Key concepts for each task are provided on the exercise handouts.
  - If the participant in unable to correct the movement pattern after instruction, you may assess the level below and repeat the process.
  - If the participant is able to correct the impaired movement pattern easily, you may assess the level above and repeat the process.
- If no impairment is present, educate the participant in the key concepts for that movement and assess for repetition prescription. They will likely begin with a higher level.

***Prescription for repetitions (Ex 1-5):** The appropriate level of exercise is one in which the participant fatigues between 8-25 reps. Begin with level determined in the previous step (appropriate level). At each tested level, if participant is able to complete > 25 reps without fatigue and using the appropriate movement pattern, assess the next level. If the therapist believes that patient can complete a higher level, a level may be skipped. Repeat until the appropriate level is determined. Use the same procedure to determine level of resistance. The number of repetitions the person can perform will be used to determine the exercise parameters. **Parameters:** Perform 2-3 sets, 8-10 reps 5x/week. Resistance can be provided with theraband, ankle weights or weight equipment. Participant is progressed to the next level when they can perform 3 sets of 8-10 repetitions with ease and demonstrating good mechanics at the hip and knee.

If patient experiences a production of or an increase in hip joint pain compared to at rest with a specific exercise, modify exercise by

1. Ensuring the patient is performing correctly.
2. Regressing to an exercise of a lower level.
3. Limit the range of motion the patient is performing.
4. If neither of the above modifications result in reduction of hip joint pain experienced, do not assign the exercise. Try the exercise the following week.

†Patient-specific tasks: The primary goal for patient-specific tasks is to improve an impaired movement pattern during performance of the task. First observe the participant's natural movement pattern. Note any excessive movement impairments such as excessive hip adduction (medial collapse pattern or excessive pelvic drop) or hip and knee hyperextension.

- If impairment is present, educate the participant in the corrected movement pattern and determine if participant can perform the corrected task independently. Key concepts will be task-specific, using similar key concepts provided for the daily task.
  - If the participant has difficulty with correcting the movement pattern, break down the movement and have them practice specific phases of the task. Once they are able to perform the task correctly, encourage to continue to use key concepts during performance of the task. The task is then progressed by increasing the time participating in the task to reach the participant's goal.
  - If the participant is able to correct the impaired movement pattern easily or if no impairment is present, encourage the participant to use the key concepts during performance of the task. The task is then progressed by increasing the time participating in the task to reach the participant's goal.

- If no impairment is present, educate the participant in the key concepts and encourage the participant to use the key concepts during performance of the task. The task is then progressed by increasing the time participating in the task to reach the participant's goal.
    - In addition to addressing movement impairments, patient-specific activities can be modified (progressed or regressed) using the general principles of loading, increasing/decreasing intensity, frequency and/or duration of the activity.

ID: ___ ___ ___

Patient Education
Movement Pattern Training

Visit #: ___

Date: ___ /___/ ___

Hip joint pain

- Hip joint pain may be due to one of more of the following:
  - Trauma, such as an accident or a fall.
  - Related to <u>repetitive movement patterns</u> used during daily activities, including work and fitness.
- Factors that may also contribute to why you have hip joint pain include:
  - Bony abnormalities of the femur or pelvis that affect the amount of motion at your hip.
  - Hip muscle imbalances (weakness, stiffness) due to performance of repetitive movement patterns.
  - Nerve Sensitization – When pain lasts longer than expected – the brain and nervous system get more sensitive, or go on "high alert." These changes make your nerves more efficient at telling your brain about what is going on in your body tissues (i.e. your hip). This means small signals from the hip that should not normally cause pain may be enough to trigger a pain response.

Components of your treatment

As a participant in this study, your treatment will include a comprehensive regimen of activities to ensure adequate attention to all of the components of your impairment / problem. In the 10 P.T. sessions, you will receive the following:

- Education regarding activity modification and pain control.
- Activity modification based on the best positioning and movements of the hip joint.
- Home exercise program to improve your positioning and movements of the hip joint during functional tasks.

Overall Goals of treatment

- Reduce stresses on the hip joint by <u>optimizing biomechanics</u> during functional tasks.
- Improve muscle performance through active practice of functional tasks.
- <u>Modify abnormal movement and alignment</u> performed during symptom-aggravating tasks.
- Reduce pain by decreasing sensitization

Education

- Pain management
  - Ice
  - Rest
- Pain-relieving medications
- Activity modification based on the best positioning and movements of the hip joint

Exercises

You will be receiving exercises to improve lower extremity alignment during daily activities. These exercises may result in muscle soreness, however the exercises should not increase your hip joint pain. If any exercise increases your hip joint pain, try the following:

1. Review exercise handout to be sure you are performing correctly. If you were performing incorrectly, correct performance. If pain is decreased with corrected performance, continue with exercise as instructed.

Patient Education
Movement Pattern Training

2. If you are performing correctly, and you are
    a. experiencing pain at the very end of the motion, reduce the range of motion that you are performing. Continue to perform the exercise with the limited motion until you see your physical therapist.
    b. experiencing increased pain after a certain number of repetitions, perform only the number you can perform without increased pain. Continue to perform the exercise at this reduced number of repetitions until you see your physical therapist.
3. If pain is not alleviated with the above corrections, discontinue the aggravating exercise until you see your physical therapist.

Activity modifications

1. Painful postures and movements may be due to an abnormal alignment between your pelvis, knees and feet while you are performing your regular daily activities. The exercises that you will be receiving will directly address any abnormal alignment or movements that you may demonstrate while you are moving throughout your day. Every move without pain causes less irritation and leads to the road to recovery!
2. See the following pages for modifications for positions, such as standing, sitting and sleeping that may also reduce stresses to the hip joint structures and help to decrease your pain.

ID: ___ ___ ___

Patient Education
Movement Pattern Training

Visit #: ___

Date: ___ /___/ ___

STANDING

Key concepts

- Avoid prolonged periods of standing if possible.
- Allow there to be a slight bend in your knees. Don’t stand with your knees locked.
- Keep your shoulders over your hips. Don’t stand with your hip swayed forward.

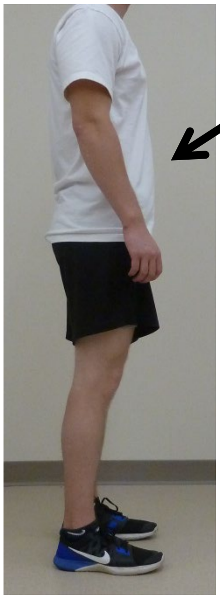

Correct:
- Shoulders over hips
- Knees slightly bent

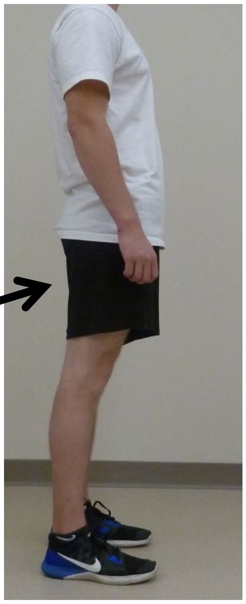

Incorrect:
- Hip swayed forward in front of shoulders
- Knees locked

- Stand with equal weight on both legs. Don’t stand with weight on one leg for prolonged period of time.

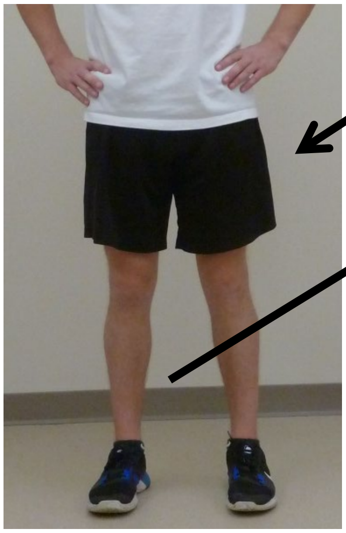

Correct:
- Equal weight (preferred)
- May switch from leg to leg

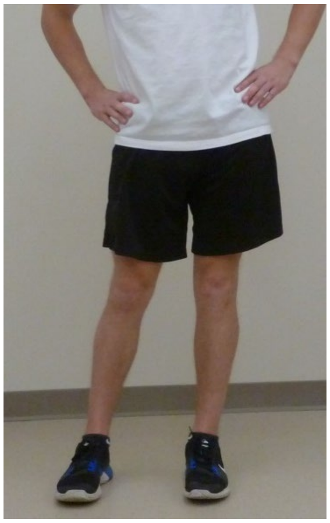

Incorrect:
- Weight shifted to left leg

ID: ___ ___ ___

Patient Education
Movement Pattern Training

Visit #: ___

Date: ___ /___/ ___

SITTING

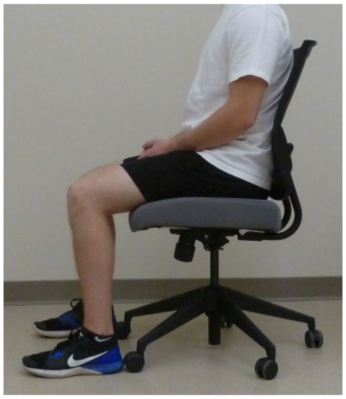

Key concepts

- Position yourself so that your knees are aligned with your ankles. While sitting, your feet should be supported (flat on the floor). Back should be supported, with shoulders positioned over hips.
- Limit amount of time sitting. At minimum, get up out of chair every 30 minutes. During times of prolonged sitting, set a timer to provide a reminder.
- Try to maintain symmetry of hips
- Try to maintain hip in neutral or unrotated position (if not possible, rolling outward slightly is better than rolling inward)

Correct

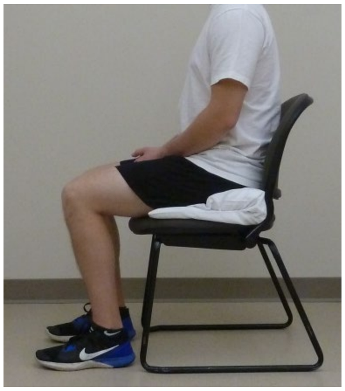

- Sit with your knees at same level of slightly higher than your hips. You may need to raise your chair or use a pillow, towel or wedge in your chair.
- If unable to sit fully back in chair, sit with as much thigh as possible supported by the seat pan. Avoid sitting at the edge of the chair.
- If feet do not touch the floor while sitting, support feet with a small stool.

Correct

- Do not cross your legs: leg around leg, thigh over thigh, ankle over knee, or sit on one leg/having one leg pulled up under you. You may cross at the ankles.

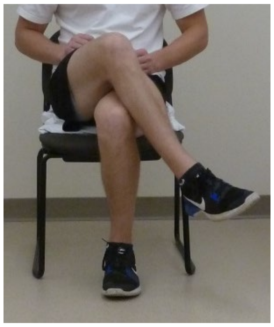

Incorrect

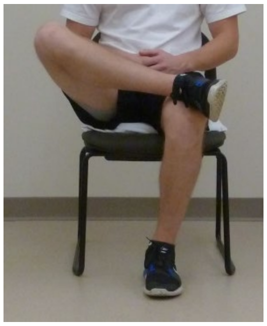

Incorrect

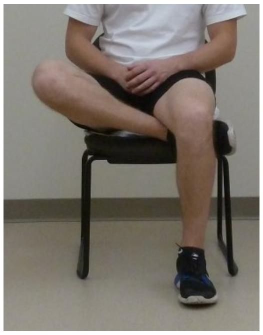

Incorrect

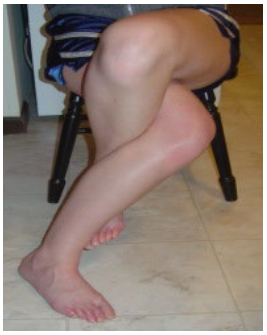

Incorrect

DRIVING: use the above concepts for sitting to adjust your seat for driving
- If your car has bucket seat, a pillow may be placed in the seat to raise the hips.
- Don't allow the thigh to roll in for prolonged periods of time.

Patient Education
Movement Pattern Training

- If you have pain when you lift your leg to change pedals, try to keep heel on the floor and pivot your foot from pedal to pedal.

SLEEPING

Key concepts

- Avoid extreme hip flexion or rotation:

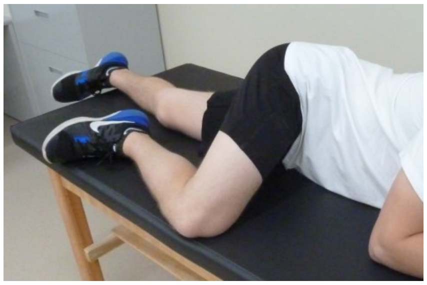

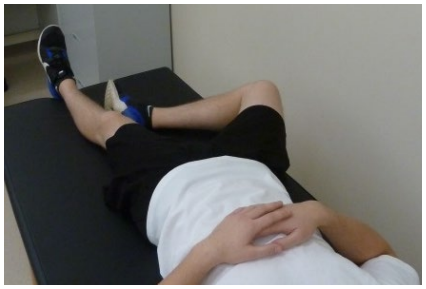

Incorrect

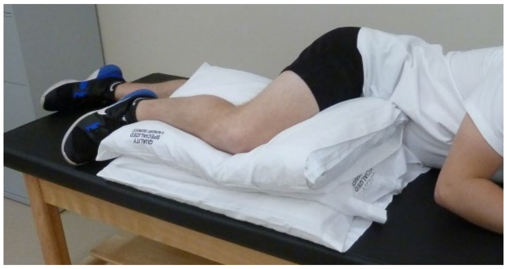

Correct

- When sleeping on your *side*, use pillows between your knees to keep your legs parallel to the floor.

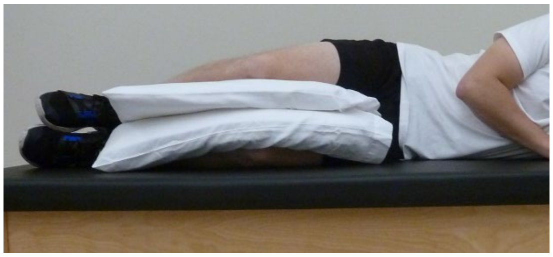

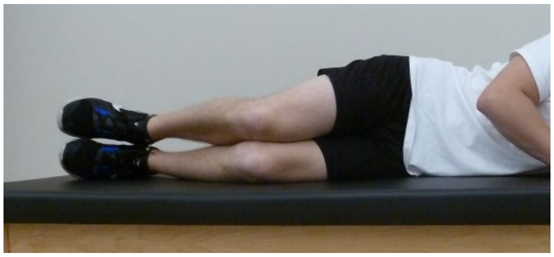

Correct

Incorrect

ID: ___ ___ ___

Patient Education
Movement Pattern Training

Visit #: ___

Date: ___ /___/ ___

SLEEPING cont.

Key concepts

If you have pain in the following positions, try these modifications:

- When sleeping on your stomach, place a pillow under your hips.

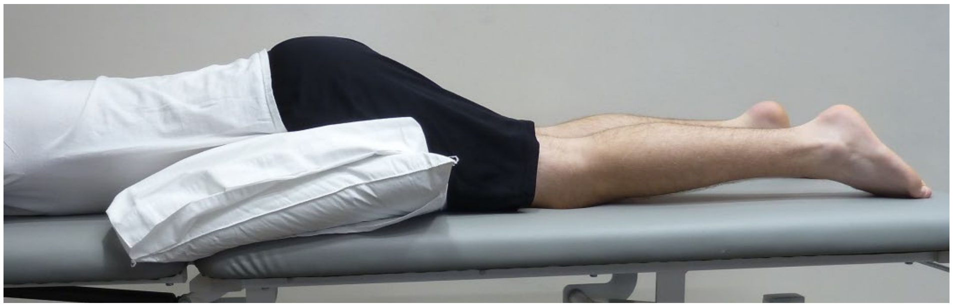

- When sleeping on your back, place a pillow under your knees.

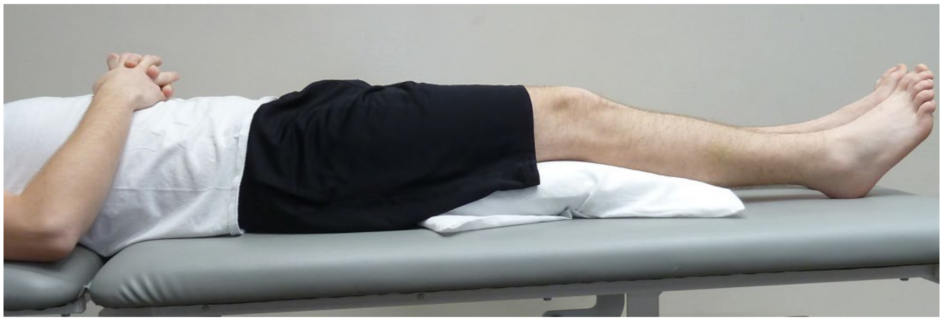

- When sleeping on your side, you may reduce pressure on your hip by
  - placing towel rolls above and below the hip bone.
  - a towel roll under the waist.

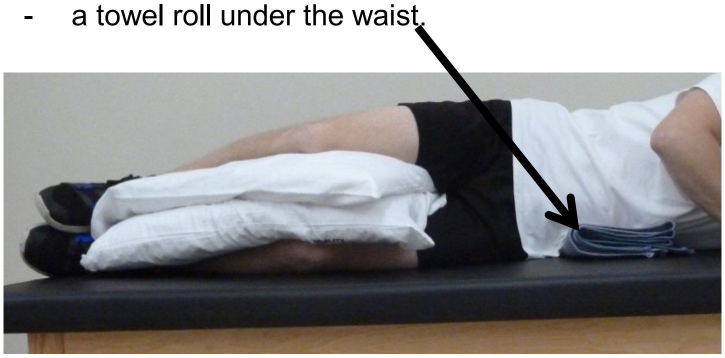

MOVING IN BED

___ When getting out of bed, roll to your side first, then sit up from the side lying position. Do not sit straight up.

___ When rolling on to your side, keep your hips in line with your shoulders. Do not twist. Roll like a log. It may be helpful to place a pillow between your knees.

ID: ___ ___ ___

Patient Education

Movement Pattern Training

Visit #: ___

Date: ___ /___/ ___

RETURN TO FITNESS

It is important to participate in physical activity to maintain or improve your overall health. Because of your injury, you may need to refrain from or limit your activities to allow for healing. Once you and your physical therapist have determined that it is time to return to your fitness routine, your physical therapist will assist you in designing a program to gradually return to your activity.

Patient Specific Tasks – MovePat

Running

Purpose: To optimize lower extremity movement pattern during running

Key Concepts

- Keep knees in line with feet
- Don't let your knee roll in, your pelvis tilt or trunk lean

Keep in Mind

- Same mechanics as walking just at faster speed (gluteals activate when same side foot hits the ground).
- Shorten stride to use gluteals more, attempt to land more on middle of foot than heel.

Progression

- if pain is experienced during a level, go down to the previous level
- stay at each level for 2 runs minimum before progressing

____ Level 1: 15 seconds run/45 seconds walk x 4

____ Level 2: 30 seconds run/1 minute walk x 4

____ Level 3: 1 minute run/1-2 minutes walk x 4 building up to 10 (4-10 minutes total of running)

____ Level 4: 2 minutes running/1 minute walk x 5 building up to 12 (10-24 minutes total of running)

____ Level 5: 3 minutes running/1 minute walk x 8 (24 minutes running)

____ Level 6: 4 minutes running/1 minute walk x 6 (24 minutes running)

____ Level 7: 6 minutes running/1 minute walk x 4 (24 minutes running)

____ Level 8: 8 minutes running/1 minute walk x 3 (24 minutes running)

____ Level 9: 12 minutes running/1 minute walk x 2 (24 minutes running)

____ Level 10: 15-20 minute run

____ Level 11: Build by 1-3 minutes per run until you are at your goal distance

Cycling

Purpose: To optimize lower extremity movement pattern during bike riding

Key Concepts

- Don't let your knee roll in
- Don't let the hips bend too much towards chest

Keep in Mind

- Think of your kneecaps like headlights on a car, keep them pointing out straight ahead or slightly out to the side.
- Keep the knees in this position whether the knee is bent or straight, sitting on the seat or not. Adjust clips if necessary.
- Increase seat height to keep hips above knees through the revolution.
- Squeeze glutes when pedaling, focus on legs pushing down rather than pulling pedal up.

Recumbent bikes

- Move seat further from pedals
- If able, recline seat to decrease hip flexion
- If the seat does not recline, slide hips forward in the seat and lean trunk back to decrease hip flexion.

Sitting on Floor

Purpose: To optimize lower extremity movement pattern during sitting on floor

Key Concepts

- Avoid prolonged position of hip flexion

Keep in Mind

- Sit on small stool or support whenever possible.
- Sitting legs crossed with thighs rotated outward is preferred over W sitting (both hips rotated inward) or sway sitting (one hip rotated outward while the other rotated inward).
- Avoid twisting the back or hip region while in crisscross, have trunk facing what you are doing.

Walking

Purpose: To optimize the lower extremity movement pattern during walking.

Key concepts

- Contract gluteals on the stance limb
- Don't let your knee roll in or your pelvis tilt
- Increase push off, keeping knee flexed

1. Use heel to toe pattern(right side demonstrated).
2. Tighten gluteals as you step onto the foot.
3. Don't allow the stance knee to roll in.
4. Gradually increase walking time to tolerance.

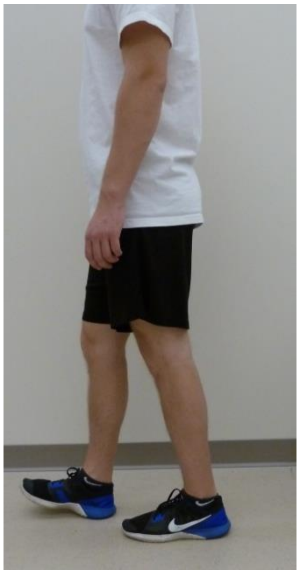

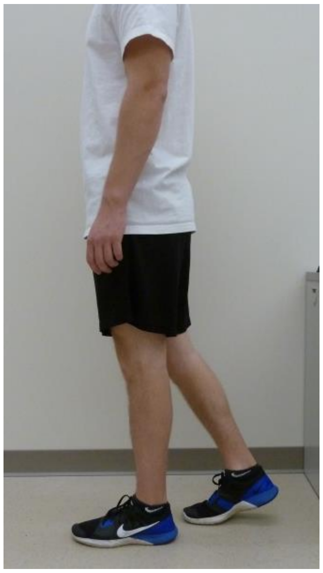

Soccer

Purpose: To optimize lower extremity movement pattern during soccer

Key Concepts

- Keep knees in line with feet
- Don't let your knee roll in or your pelvis tilt
- Keep trunk square and erect, avoid excessive side bending

Keep in Mind

- Incorporate concepts learned with running including gradual progression of pain free activity

Intercourse

Purpose: To optimize lower extremity movement pattern during intercourse

Key Concepts

- Minimal hip bend
- Don’t allow the knees to roll in

Keep in Mind

- When on back, support legs whenever possible with pillows/blankets, and aim for symmetry of hip position.
- When lying on side, place a pillow between legs if possible
- Sitting legs crossed with thighs rotated outward is preferred over W sitting (both hips rotated inward) or sway sitting (one hip rotated outward while the other rotated inward).
- Crisscross is better than
- Steer away from positions where your knees or hip roll inward.
- Avoid or modify positions where the knees are close to the chest, don’t bend hip more than 90 degrees.
- Avoid or modify positions where the knees move toward each other, widen the knees instead of narrowing them.

Example of good position: on your back with legs in “frog” position, with pillows under things and knees.

Weightlifting

Purpose: To optimize lower extremity movement pattern during weightlifting

Key Concepts

- Keep knees in line with feet
- Don’t let your knee roll in or your pelvis tilt

Keep in Mind

- Widen stance with knees and hips slightly bent
- With squatting techniques, limit depth of the squat
- Activate your abdominals to prevent arching the back or tilting the pelvis forward.
- Use a stool/chair for prolonged positions of hip flexion.
- Keep knees over 2nd and 3rd toes, squeeze glutes when coming up.

Hiking

Purpose: To optimize lower extremity movement pattern during hiking

Key Concepts

- Keep knees in line with feet
- Don’t let your knee roll in, your pelvis tilt or trunk lean

Keep in Mind

- Take smaller steps, increase push off with back foot, and squeeze glutes of standing side.
- Don't hyperextend, overly straingthen your hips or knees.
- Decrease speed, incline, and duration.
-

Yoga

Purpose: To optimize lower extremity movement pattern during yoga

Key Concepts

- Don’t let your knee roll in or your pelvis tilt
- Keep trunk square and erect, avoid excessive side bending
- Avoid prolonged positions into hip flexion

Keep in Mind

- Decrease range of yoga poses to limit amount of maximum hip bend
- Avoid painful endrange stretching

Driving Long Distances

Purpose: To optimize lower extremity movement pattern during long distance driving

Key Concepts

- Keep knees in line with feet
- Don’t let your knee roll in, your pelvis tilt or trunk lean

Other options

- Place folded towel or small pillow under the hips so that hips are higher than the knees.

Stretching

Purpose: To optimize lower extremity movement pattern during stretching

Key Concepts

- Don’t let your knee roll in, your pelvis tilt or trunk lean
- Avoid prolonged positions into hip bend

Keep in Mind

- Decrease range of stretch to limit amount of maximum hip bend
- Avoid painful endrange stretching

Sit to Stand, Squat – Level 1
Lowering slowly

Purpose: To optimize the lower extremity movement pattern during sit to stand.

Key concepts

- Slide to edge of seat
- Keep knees in line with feet
- Don’t allow the knees to roll in

1. Stand with feet hip-width apart.
2. Bend at your hips and knees while simultaneously contracting your thigh and gluteal muscles to slowly lower yourself onto the chair.
3. Keep the knees aligned with the toes. Don’t allow knees to roll in.
4. Return to the starting position.

Perform: ______sets, _____reps, 5 days/week **Goal: 3 sets, 8-10 reps, 5 days/week**

Continue to use the instructed strategy when you perform sit to stand during the day.

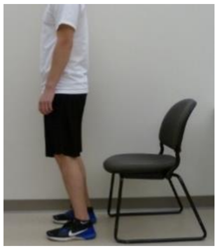
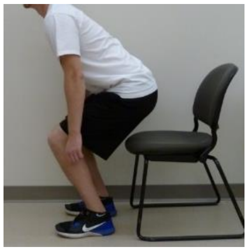
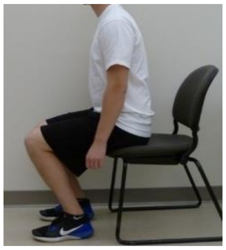
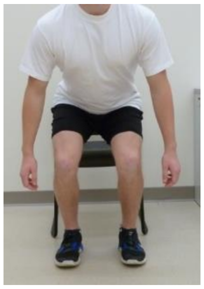
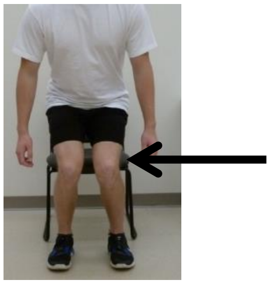

Sit to Stand, Squat – Level 2
Lowering slowly with resistance

Purpose: To optimize the lower extremity movement pattern during sit to stand.

Key concepts

- Slide to edge of seat
- Keep knees in line with feet
- Don’t allow the knees to roll in

1. Place theraband around both legs, just above the knee (see picture). Theraband should be snug.
2. Stand with feet hip-width apart.
3. Bend at your hips and knees while simultaneously contracting your thigh and gluteal muscles to slowly lower yourself onto the chair.
4. Keep the knees aligned with the toes. Don’t allow knees to roll in.
5. Return to the starting position.

Theraband: yellow (canary) red green blue black grey yellow (gold)

Perform: ______sets, _____reps, 5 days/week **Goal: 3 sets, 8-10 reps, 5 days/week**

Continue to use the instructed strategy when you perform sit to stand during the day.

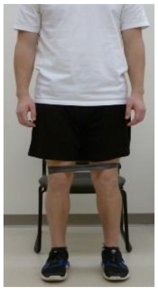
Correct start position

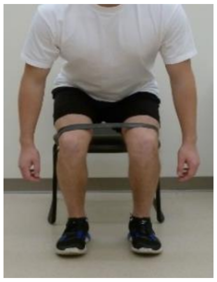
Correct

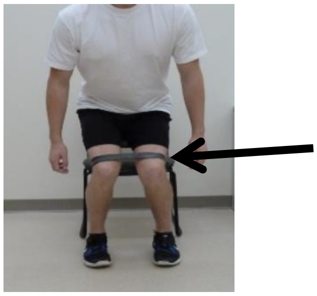
Incorrect

Sit to Stand, Squat – Level 3
Slowly lower with resistance + tap

Purpose: To optimize the lower extremity movement pattern during sit to stand.

Key concepts

- Slide to edge of seat
- Keep knees in line with feet
- Don’t allow the knees to roll in

1. Place theraband around both legs, just above the knee (see picture). Theraband should be snug.
2. Stand with feet hip-width apart.
3. Bend at your hips and knees while simultaneously contracting your thigh and gluteal muscles to slowly lower yourself towards the chair.
4. **Tap the buttocks** to the chair and return to the starting position. Do not sit.
5. Keep the knees aligned with the toes. Don’t allow knees to roll in.

Theraband: yellow (canary)   red   green   blue   black   grey   yellow (gold)

Perform: ______sets, _____reps, 5 days/week   **Goal: 3 sets, 8-10 reps, 5 days/week**

Continue to use the instructed strategy when you perform sit to stand during the day.

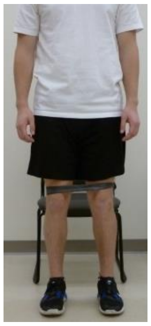

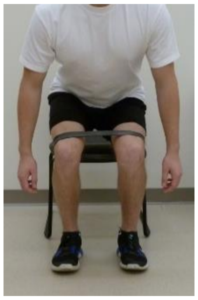

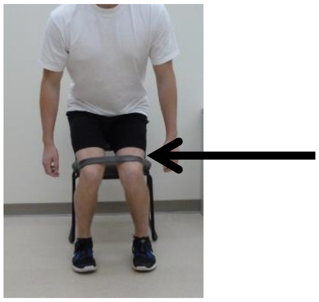

Correct start position   Correct   Incorrect

Sit to Stand, Squat-Level 4
Bilateral squat

Purpose: To optimize the lower extremity movement pattern during sit to stand.

Key concepts

- Keep knees in line with feet
- Don’t allow the knees to roll in

1. Stand with feet hip-width apart.
2. Bend at your hips and knees while simultaneously contracting your thigh and gluteal muscles to slowly lower yourself down. Lower to ____________________
3. Keep the knees aligned with the toes. Don’t allow knees to roll in.
4. You may place your arms in front of you to maintain balance.
5. Return to the starting position.

Perform: ______sets, _____reps, 5 days/week   **Goal: 3 sets, 8-10 reps, 5 days/week**

Continue to use the instructed strategy when you perform sit to stand during the day.

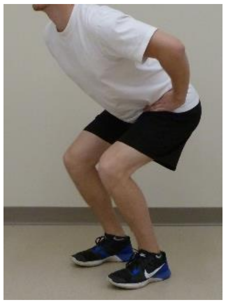

Sit to Stand, Squat – Level 5
Bilateral squat with resistance

Purpose: To optimize the lower extremity movement pattern during sit to stand.

Key concepts

- Keep knees in line with feet
- Don't allow the knees to roll in

1. Place theraband around both legs, just above the knee (see picture). Theraband should be snug.
2. Bend at your hips and knees while simultaneously contracting your thigh and gluteal muscles to slowly lower yourself down. Lower to ____________________
3. Keep the knees aligned with the toes. Don't allow knees to roll in.
4. You may place your arms in front of you to maintain balance.
5. Return to the starting position.

Theraband: yellow (canary) red green blue black grey yellow (gold)

Perform: ______sets, _____reps, 5 days/week **Goal: 3 sets, 8-10 reps, 5 days/week**

Continue to use the instructed strategy when you perform sit to stand during the day.

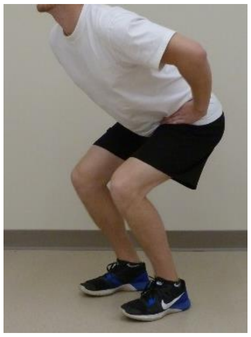

Single Leg Activities – Level 1
Weight Shift

Purpose: To optimize the lower extremity movement pattern while performing single limb activities.

Key concepts

- Contract gluteals on stance limb
- Don't let your knee roll in, your pelvis tilt or trunk lean

1. Stand with both feet relatively close together.
2. Both knees should be "unlocked".
3. Shift your weight to one leg.
4. Tighten your gluteal muscle on the side of your stance leg.
5. Repeat to the opposite leg.
6. Alternate the weight shift from one leg to the other.
7. Keep your pelvis level and trunk upright.
8. Don't let your knee turn inward or your pelvis tilt.
9. You may place your hands on your pelvis to monitor your motion.

Perform: ______sets, _____reps, 5 days/week **Goal: 3 sets, 8-10 reps, 5 days/week**

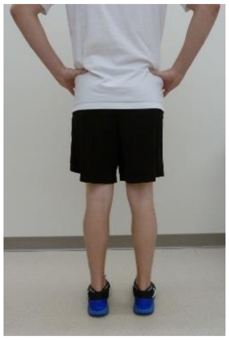
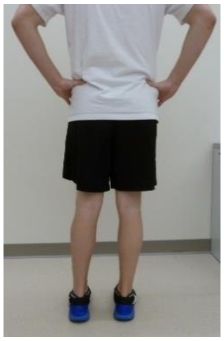
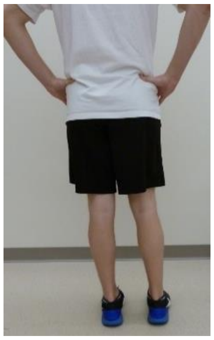

Single Leg Activities – Level 2
Single leg stance

Purpose: To optimize the lower extremity movement pattern while performing single limb activities.

Key concepts

- Contract gluteals on stance limb
- Don't let your knee roll in, your pelvis tilt or trunk lean

1. Perform weight shift (level 1) to one leg.
2. Tighten your gluteal muscle on the side of your stance leg.
3. Lift your opposite leg in front of you as if marching in place.
4. Repeat to the opposite leg.
5. Alternate the weight shift from one leg to the other.
6. Keep your pelvis level and trunk upright.
7. Don't let your knee turn inward or your pelvis tilt.
8. You may place your hands on your pelvis to monitor your motion.

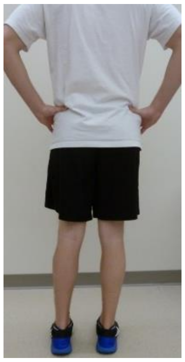

Correct shift

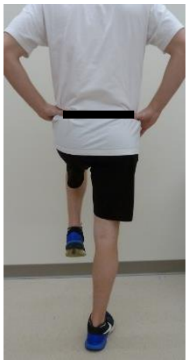

Correct
Note hands at same level

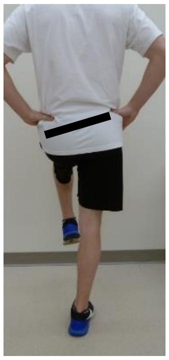

Incorrect
Note pelvis tilt left hand lower than right

___Option: Hold ______ seconds.

___Option: Hold onto countertop for balance.

___Option: If hip pain is reproduced during hip flexion, flex knee behind you.

Perform: ______sets, _____reps, 5 days/week **Goal: 3 sets, 8-10 reps, 5 days/week**

Single Leg Activities – Level 3
Single leg squat with hand support

Purpose: To optimize the lower extremity movement pattern while performing single limb activities.

Key concepts

- Contract gluteals on stance limb
- Don't let your knee roll in, your pelvis tilt or trunk lean

1. Hold onto countertop for balance.
2. Perform single leg stance (level 2).
3. Slowly lower self into a squat (like you are going to sit in a chair). Do not allow the knee you are standing on to collapse in or rotate toward your other leg.
4. Once lowered, push back up to start position by squeezing your gluteals. Again it is important to not let the knee roll in.
5. Only go deep enough where the movement is controlled on the way down and up.

Perform: ______sets, _____reps, 5 days/week **Goal: 3 sets, 8-10 reps, 5 days/week**

Repeat for the opposite side.

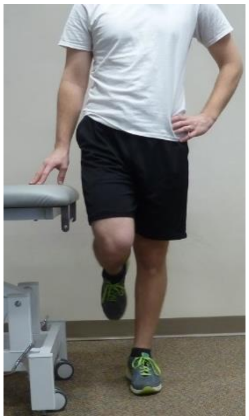

Correct start

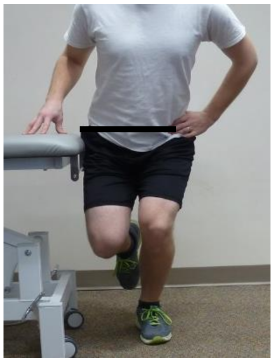

Correct

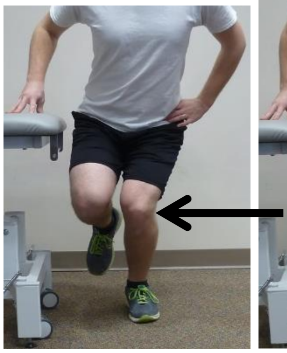

Incorrect

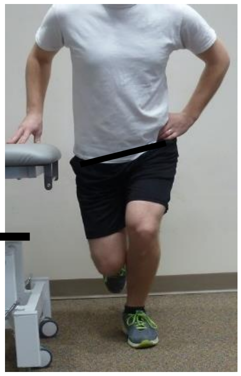

Incorrect

Single Leg Activities – Level 4
Single leg squat without hand support

Purpose: To optimize the lower extremity movement pattern while performing single limb activities.

Key concepts

- Contract gluteals on stance limb
- Don’t let your knee roll in, your pelvis tilt or trunk lean

1. Perform single leg stance (level 2).
2. Slowly lower self into a squat (like you are going to sit in a chair). Do not allow the knee you are standing on to collapse in or rotate toward your other leg.
3. Once lowered, push back up to start position by squeezing your gluteals. Again it is important to not let the knee collapse in or rotate.
4. Only go deep enough where the movement is controlled on the way down and up.

Perform: ______sets, _____reps, 5 days/week
Goal: 3 sets, 8-10 reps, 5 days/week
Repeat for the opposite side.

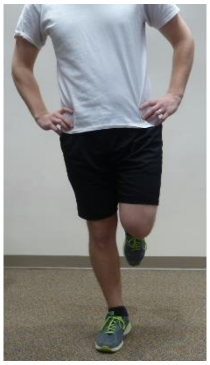
Correct

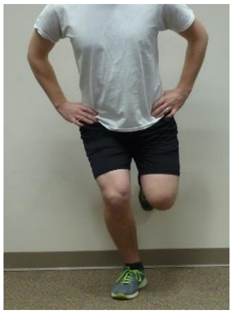
Correct
(Keep the lifted leg under the hip)

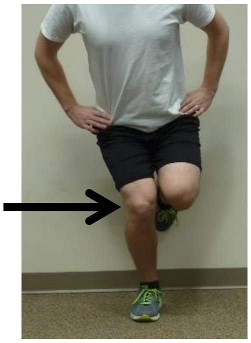
Incorrect

Single Leg Activities – Level 5
Single leg squat with resistance

Purpose: To optimize the lower extremity movement pattern while performing single limb activities.

Key concepts

- Contract gluteals on stance limb
- Don’t let your knee roll in, your pelvis tilt or trunk lean

1. Place theraband around the leg to be exercised (see picture). Theraband should be snug.
2. Perform single leg stance (level 2).
3. Slowly lower self into a squat (like you are going to sit in a chair). Do not allow the knee you are standing on to collapse in or rotate toward your other leg.
4. Once lowered, push back up to start position by squeezing your gluteals. Again it is important to not let the knee collapse in or rotate.
5. Only go deep enough where the movement is controlled on the way down and up.

Theraband: yellow (canary) red green blue black grey yellow (gold)

Perform: ____sets, ___reps, 5 days/week
Goal: 3 sets, 8-10 reps, 5 days/week
Repeat for the opposite side.

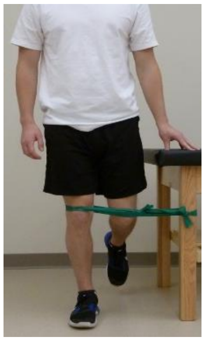
Correct

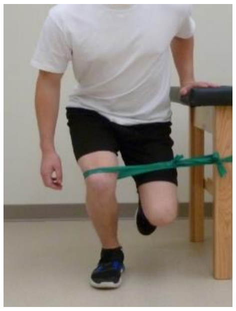
Correct

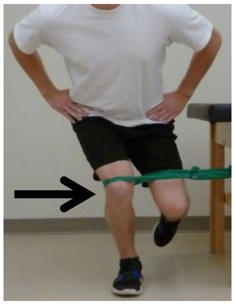
Incorrect

(may use light touch with hand for balance if needed)

Stair Ambulation – Level 1
Step up/down stairs with hand support

Purpose: To optimize the lower extremity movement pattern while using stairs.

Key concepts

- Contract gluteals on stance limb
- Don't let your knee roll in or your pelvis tilt

Going up:

1. Use a handrail to assist with the movement
2. Lean forward and use your thigh and gluteal muscles to move your body forward to the next stair.
3. Don't let your knee roll in or your pelvis tilt.
4. Avoid pulling your knee back to meet the body. Instead propel the body forward to the next stair.

Going down:

1. Use a handrail to assist with the movement
2. Use your thigh and gluteal muscles to slowly lower yourself onto the next stair.
3. Don't let your knee roll in or your pelvis tilt excessively.
4. Do not use momentum to perform the activity.

Perform: ______sets, _____reps, 5 days/week **Goal: 3 sets, 8-10 reps, 5 days/week**

___Option: if difficult to perform correctly, a smaller step height may be used.

Repeat for the opposite side.

Practice the instructed strategy when you are using the stairs during the day.

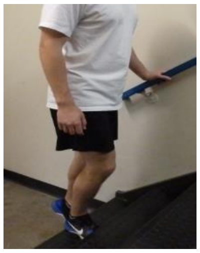
Correct

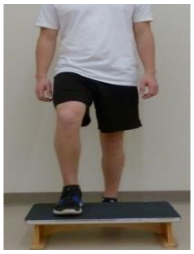
Correct

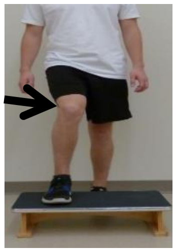
Incorrect

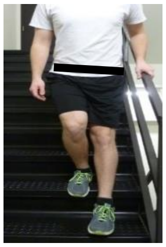
Correct

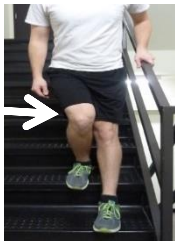
Incorrect

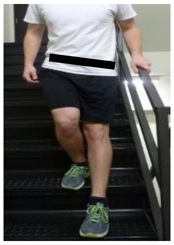
Incorrect

Stair Ambulation – Level 2
Step up/down stairs without hand support

Purpose: To optimize the lower extremity movement pattern while using stairs.

Key concepts

- Contract gluteals on stance limb
- Don't let your knee roll in or your pelvis tilt

Going up:

1. Lean forward and use your thigh and gluteal muscles to move your body forward to the next stair.
2. Don't let your knee roll in or your pelvis tilt.
3. Avoid pulling your knee back to meet the body. Instead propel the body forward to the next stair.

Going down:

1. Use your thigh and gluteal muscles to slowly lower yourself onto the next stair.
2. Don't let your knee roll in or your pelvis tilt excessively.
3. Do not use momentum to perform the activity.

Perform: ______sets, _____reps, 5 days/week **Goal: 3 sets, 8-10 reps, 5 days/week**

___Option: if difficult to perform correctly, a smaller step height may be used.

Repeat for the opposite side.

Continue to use the instructed strategy when using the stairs during the day.

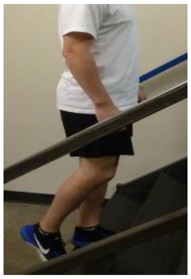
Correct

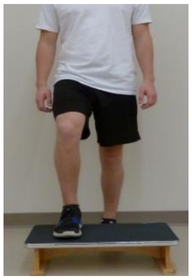
Correct

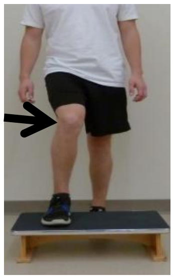
Incorrect

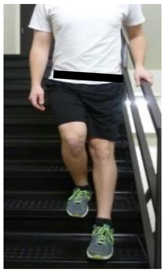
Correct

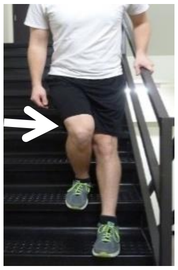
Incorrect

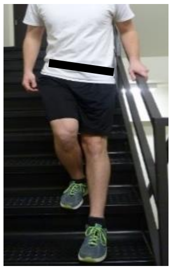
Incorrect

Stair Ambulation – Level 3
Step up/down stairs – increase speed (no resistance)

Purpose: To optimize the lower extremity movement pattern while using stairs.

Key concepts
- Contract gluteals on stance limb
- Don't let your knee roll in or your pelvis tilt

Going up:
1. Perform step up with increased speed.
2. Lean forward and use your thigh and gluteal muscles to move your body forward to the next stair.
3. Don't let your knee roll in or your pelvis tilt.
4. Avoid pulling your knee back to meet the body. Instead propel the body forward to the next stair.

Going down:
1. Use your thigh and gluteal muscles to slowly lower yourself onto the next stair.
2. Don't let your knee roll in or your pelvis tilt excessively.
3. Do not use momentum to perform the activity.

Perform: ______sets, _____reps, 5 days/week **Goal: 3 sets, 8-10 reps, 5 days/week**

Repeat for the opposite side.

Continue to use the instructed strategy when using the stairs during the day.

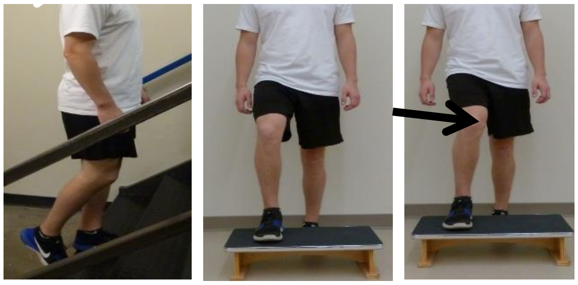

Correct Correct Incorrect

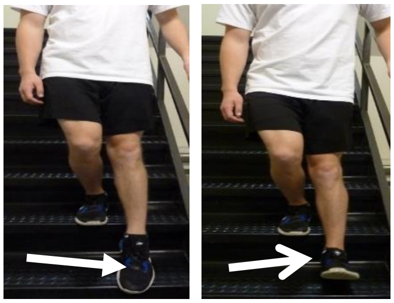

Correct with toe tap Correct with heel tap

Stair Ambulation – Level 4 and 5
Step up/down stairs with resistance

Purpose: To optimize the lower extremity movement pattern while using stairs.

Key concepts
- Contract gluteals on stance limb
- Don't let your knee roll in or your pelvis tilt

Going up:
1. Use a taller step or place theraband around the leg to be exercised (see picture). Theraband should be snug.
2. Lean forward and use your thigh and gluteal muscles to move your body forward to the next stair.
3. Don't let your knee roll in or your pelvis tilt.
4. Avoid pulling your knee back to meet the body. Instead propel the body forward to the next stair.

Going down:
1. Use a taller step or place theraband around the leg to be exercised (see picture). Theraband should be snug.
2. Use your thigh and gluteal muscles to slowly lower yourself onto the next stair.
3. Don't let your knee roll in or your pelvis tilt excessively.
4. Do not use momentum to perform the activity.

Theraband: yellow (canary) red green blue black grey yellow (gold)

Repeat for the opposite side.

Perform: ______sets, _____reps, 5 days/week **Goal: 3 sets, 8-10 reps, 5 days/week**

Continue to use the instructed strategy when using the stairs during the day.

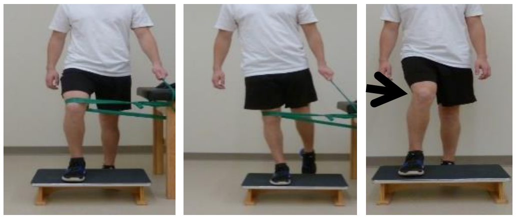

Correct Correct Incorrect

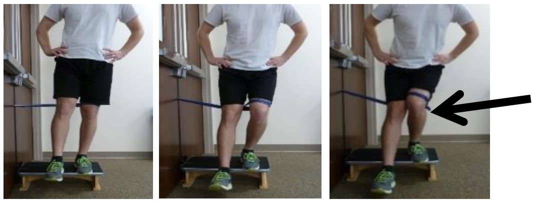

Correct Start Correct Incorrect

Side Stepping – Level 1
Initial step with tap

Purpose: To optimize the lower extremity movement pattern while performing activities with lateral movement.

Key concepts

- Contract gluteals on stance limb
- Don’t let either knee roll in or your pelvis tilt

1. Stand with both feet relatively close together.
2. Both knees should be “unlocked”.
3. Shift your weight to one leg.
4. Tighten your gluteal muscles on the side of your stance leg.
5. Lift your opposite leg out to the side, tap the toe on the ground and return to the starting position.
6. Don’t allow the knee to roll in or your pelvis tilt.
7. Repeat

Perform: ______sets, _____reps, 5 days/week **Goal: 3 sets, 8-10 reps, 5 days/week**

Repeat exercise on the opposite side.

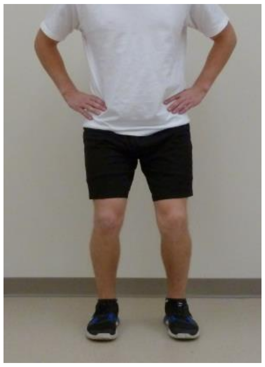

Correct

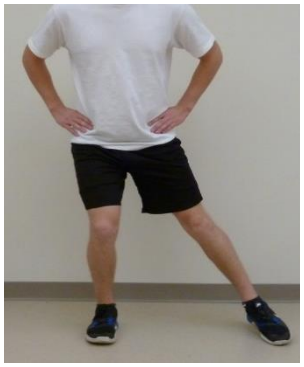

Correct

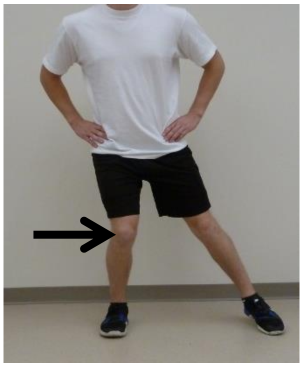

Incorrect

Side Stepping – Level 2
Side Step

Purpose: To optimize the lower extremity movement pattern while performing activities with lateral movement.

Key concepts

- Contract gluteals on stance limb
- Don’t let either knee roll in or your pelvis tilt

1. Stand with both feet relatively close together.
2. Both knees should be “unlocked”.
3. Shift your weight to one leg.
4. Tighten your gluteal muscles on the side of your stance leg.
5. Step to the side with the opposite foot.
6. Bring the stance leg toward the stepping foot.
7. Keep your pelvis level and trunk upright.
8. Don’t allow the knee to roll in or your pelvis tilt during stance of either limb.
9. Repeat

Perform: ______sets, _____reps, 5 days/week **Goal: 3 sets, 8-10 reps, 5 days/week**

Repeat exercise in the opposite direction.

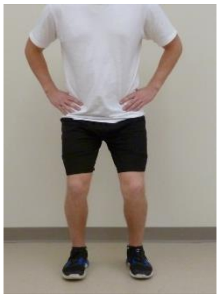

Correct

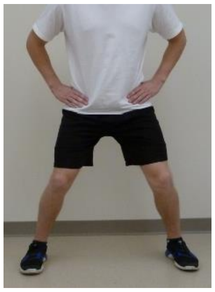

Correct

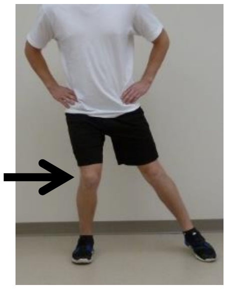

Incorrect

Side Stepping – Level 3 &4
Side Step with resistance

Purpose: To optimize the lower extremity movement pattern while performing activities with lateral movement.

Key concepts

- Contract gluteals on stance limb
- Don't let either knee roll in or your pelvis tilt

1. Wrap theraband around both legs, just above the knee (see picture) or ankle. Theraband should be snug.
2. Stand with both feet relatively close together.
3. Shift your weight to one leg.
4. Tighten your gluteal muscle on your stance leg.
5. Step to the side with the opposite foot.
6. Slowly bring the stance leg toward the stepping foot.
7. Don't allow the knee to roll in or your pelvis tilt during stance of either limb.
8. Repeat, maintaining pressure against theraband.

Theraband: yellow (canary) red green blue black grey yellow (gold)

Perform: ______sets, _____reps, 5 days/week **Goal: 3 sets, 8-10 reps, 5 days/week**

Repeat exercise in the opposite direction.

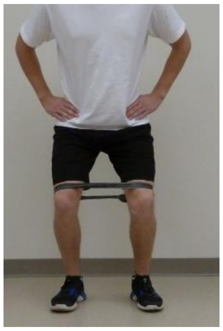

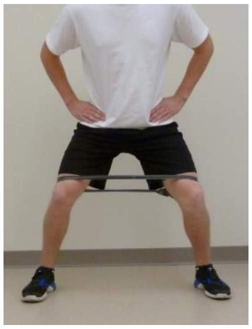

Side Stepping – Level 5
Side Step with resistance and increased speed

Purpose: To optimize the lower extremity movement pattern while performing activities with lateral movement.

Key concepts

- Contract gluteals on stance limb
- Don't let either knee roll in or your pelvis tilt

1. Stand with both feet relatively close together.
2. Shift your weight to one leg.
3. Tighten your gluteal muscle on your stance leg.
4. Step to the side with the opposite foot.
5. Wrap theraband around both legs, just above the knee (see picture) or ankle. Theraband should be snug.
6. Slowly bring the stance leg toward the stepping foot.
7. Don't allow the knee to roll in or your pelvis tilt during stance of either limb.
8. Repeat, maintaining pressure against theraband.

Theraband: yellow (canary) red green blue black grey yellow (gold)

Perform: ______sets, _____reps, 5 days/week **Goal: 3 sets, 8-10 reps, 5 days/week**

Repeat exercise in the opposite direction.

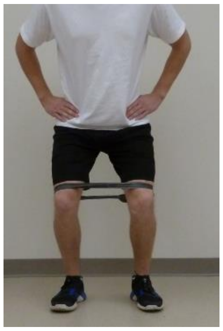

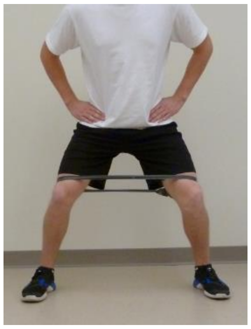

Stance with movement – Level 1
Large arc

Purpose: To optimize movement of hip and pelvis during walking/running.

Key concepts

- Contract gluteals on the stance limb
- Don't let your knee roll in or your pelvis tilt

1. Stand with one foot on a sturdy step.
2. Tighten the gluteal muscles on the stance limb.
3. Using a large arc of motion, swing the opposite leg forward as if taking a step.
4. Then swing the leg back behind you.
5. Don't let your knee roll in or your pelvis tilt.
6. Repeat.

Perform: ______sets, _____reps, 5 days/week **Goal: 3 sets, 8-10 reps, 5 days/week**

___Option: For safety, may use light touch for balance

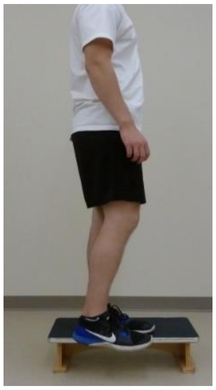
Correct

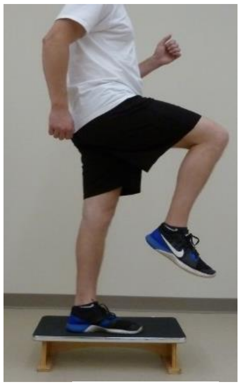
Correct

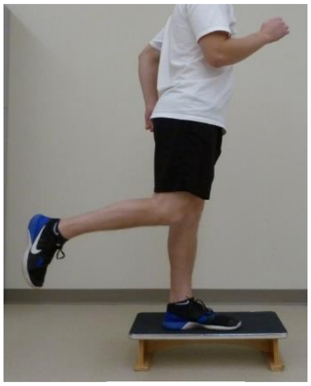
Correct

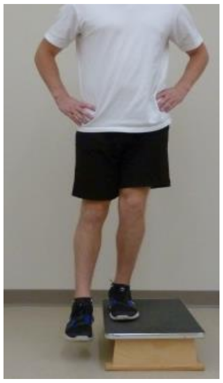
Incorrect

Stance with movement – Level 2
Large arc, fast speed

Purpose: To optimize movement of hip and pelvis during walking/running.

Key concepts

- Contract gluteals on the stance limb
- Don't let your knee roll in or your pelvis tilt

1. Stand with one foot on a sturdy step.
2. Tighten the gluteal muscles on the stance limb.
3. Using a large arc of motion, swing the opposite leg forward as if taking a step.
4. Then quickly swing the leg back behind you.
5. Don't let your knee roll in or your pelvis tilt.
6. Repeat maintaining faster speed.

Perform: ______sets, _____reps, 5 days/week **Goal: 3 sets, 8-10 reps, 5 days/week**

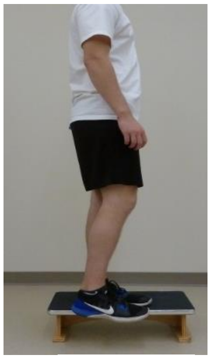
Correct

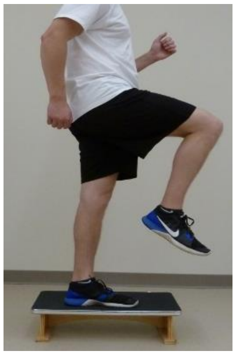
Correct

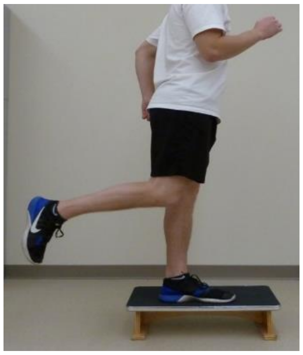
Correct

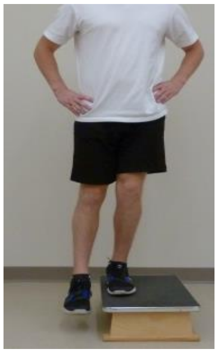
Incorrect

Stance with movement – Level 3
Large arc, fast speed (compliant surface)

Purpose: To optimize movement of hip and pelvis during walking/running.

Key concepts

- Contract gluteals on the stance limb
- Don't let your knee roll in or your pelvis tilt

1. Stand with one foot on a pillow, foam or balance disk.
2. Tighten the gluteal muscles on the stance limb.
3. Using a large arc of motion, swing the opposite leg forward as if taking a step.
4. Then quickly swing the leg back behind you.
5. Don't let your knee roll in or your pelvis tilt.
6. Repeat maintaining faster speed.

Perform: ______sets, _____reps, 5 days/week **Goal: 3 sets, 8-10 reps, 5 days/week**

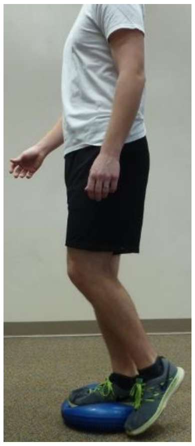

Correct

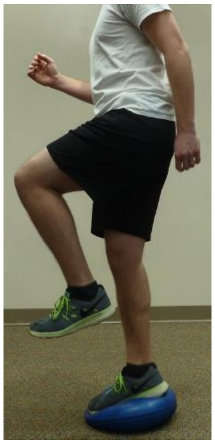

Correct

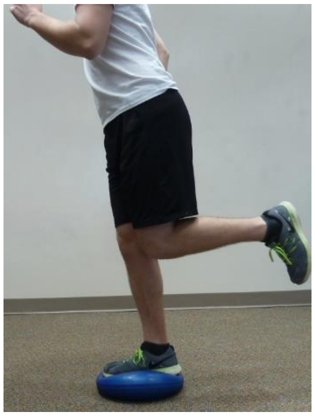

Correct

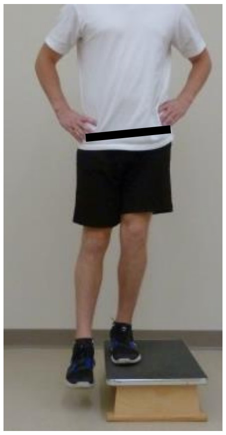

Incorrect
Note pelvis tilt

Stance with movement – Level 4
Running-like

Purpose: To optimize movement of hip and pelvis during walking/running.

Key concepts

- Contract gluteals on the stance limb
- Don't let your knee roll in or your pelvis tilt

1. Stand with one foot on a sturdy step.
2. Tighten the gluteal muscles on the stance limb.
3. Using a large arc of motion, swing the opposite leg forward quickly as if in a sprint.
4. Then quickly swing the leg back behind you.
5. Don't let your knee roll in or your pelvis tilt.
6. Repeat maintaining faster speed.

Perform: ______sets, _____reps, 5 days/week **Goal: 3 sets, 8-10 reps, 5 days/week**

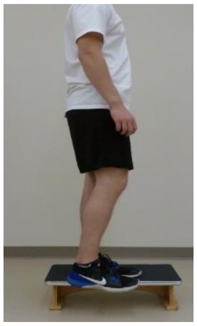

Correct

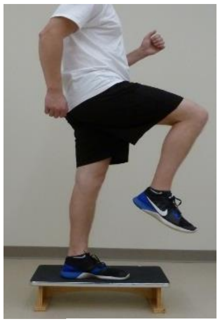

Correct

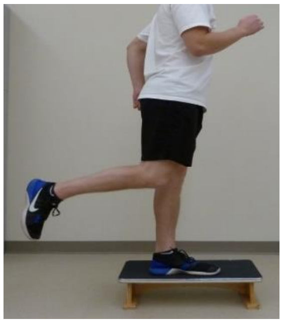

Correct

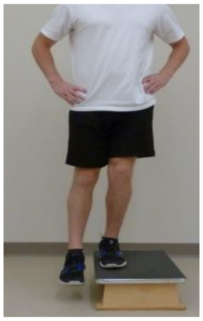

Incorrect
Note
pelvis tilt

Stance with movement – Level 5
Running-like (compliant surface)

Purpose: To optimize movement of hip and pelvis during walking/running.

Key concepts

- Contract gluteals on the stance limb
- Don't let your knee roll in or your pelvis tilt

1. Stand with one foot on a pillow, foam or balance disk.
2. Tighten the gluteal muscles on the stance limb.
3. Using a large arc of motion, swing the opposite leg forward quickly as if in a sprint.
4. Then quickly swing the leg back behind you.
5. Don't let your knee roll in or your pelvis tilt.
6. Repeat maintaining faster speed.

Perform: ______sets, _____reps, 5 days/week **Goal: 3 sets, 8-10 reps, 5 days/week**

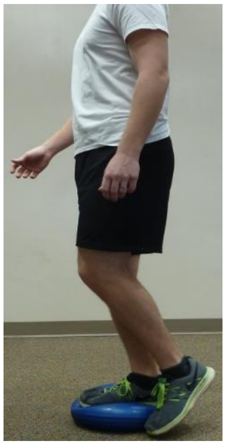
Correct

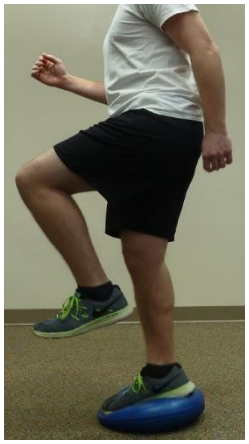
Correct

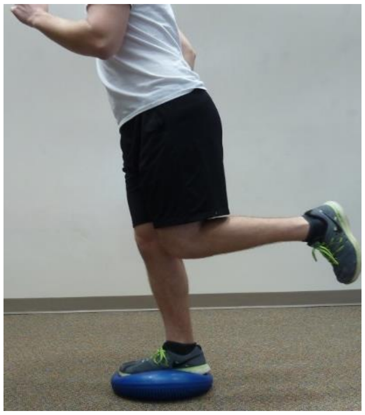
Correct

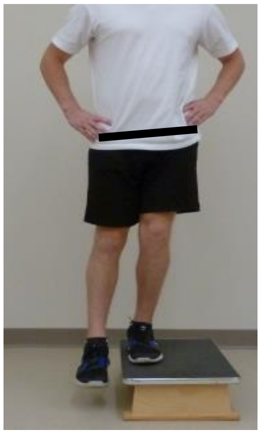
Incorrect
Note pelvis tilt

Protocol Modifications

The content in this appendix has been modified and is provided with permission from Harris-Hayes et al. Movement pattern training compared with standard strengthening and flexibility among patients with hip-related groin pain: results of a pilot multicentre randomised clinical trial. *BMJ Open Sport Exer Med.* 2020;6:e000707.

1. Within patient education handout
   - In the current study, we incorporated new information related to peripheral and central sensitization.
   - To provide each patient with a succinct summary of their assigned treatment, we added a section titled "Components of your treatment"
   - We provided more specific instruction for a varied range of seated positions a patient may encounter on a daily basis.
2. To reduce the total time a patient spent on their HEP and improve adherence to the exercise program, the number of prescribed exercise/tasks were reduced from 8 in the previous study to 5 in the current study. This modification was in response to patient and physical therapist feedback from the previous study.
3. Parameters for the exercise prescription were changed from 1x/day to 5x/week to allow more flexibility to fit around a patient's schedule and improve adherence. This modification was in response to patient and physical therapist feedback from the previous study.
4. Many exercises were revised to add more difficult levels as some patients in the previous study were reaching maximum levels early on in their course of treatment.
5. In the prior study, some daily tasks, such as walking, were included in all patients' HEP. In the current study, these tasks were only included in a patient's HEP if the patient reported pain with that daily task.
6. During the previous study, we recorded the patient specific tasks identified by the patients as symptomatic. Prior to launching the current study, we developed new written instruction sheets for the key concepts to be used during performance of those identified tasks.
7. Minor editorial changes were made within the document. These changes did not affect the instruction of the task.

References

1. Harris-Hayes M, Czuppon S, Van Dillen LR, et al. Movement-pattern training to improve function in people with chronic hip joint pain: a feasibility randomized clinical trial. *J Orthop Sports Phys Ther.* 2016;46:452-461. PMCID: PMC4889512.
2. Harris-Hayes M, Steger-May K, Bove AM, et al. Movement pattern training compared with standard strengthening and flexibility among patients with hip-related groin pain: results of a pilot multicentre randomised clinical trial. *BMJ Open Sport Exer Med.* 2020;6:e000707. PMCID: PMC7254120.
3. Harris-Hayes M, Steger-May K, A MB, Mueller MJ, Clohisy JC, Fitzgerald GK. One-year outcomes following physical therapist-led intervention for chronic hip-related groin pain: Ancillary analysis of a pilot multicenter randomized clinical trial. *J Orthop Res.* 2021;39:2409-2418. PMCID: PMC8285461.
4. Sahrmann SA. *Diagnosis and treatment on movement impairment syndromes.* St. Louis: Mosby, Inc; 2002.
5. Van Dillen LR, Norton BJ, Sahrmann SA, et al. Efficacy of classification-specific treatment and adherence on outcomes in people with chronic low back pain. A one-year follow-up, prospective, randomized, controlled clinical trial. *Man Ther.* 2016;24:52-64. PMCID: PMC4921225.
6. Van Dillen LR, Sahrmann SA, Norton BJ, Caldwell CA, McDonnell MK, Bloom N. The effect of modifying patient-preferred spinal movement and alignment during symptom testing in patients with low back pain: a preliminary report. *Arch Phys Med Rehabil.* 2003;84:313-322.
7. Salsich GB, Yemm B, Steger-May K, Lang CE, Van Dillen LR. A feasibility study of a novel, task-specific movement training intervention for women with patellofemoral pain. . *Clin Rehabil* 2018;32:179-190.
