# Supplementary material for: Comparison of Joint Mobilization and Movement Pattern Training for Patients With Hip-Related Groin Pain: A Pilot Randomized Clinical Trial
Source: Phys Ther. 2023 Aug 22;103(11):pzad111. doi: 10.1093/ptj/pzad111 (PMC10683042; doi:10.1093/ptj/pzad111)
Supplement: c_appendix_3_final_pzad111 [file c_appendix_3_final_pzad111.pdf]

2D Kinematic Assessment Methods

Examiner

The primary author (XXX) performed all data collection, developed the measurement methods and trained the research assistant who completed the measurements. The primary author has 25 years of clinical experience and 17 years of experience in kinematic data collection and assessment. Both the examiner and the research assistant were blinded to the patient's assigned treatment group.

Set up

To assist with measurement, five markers were placed on the patient: one at each ASIS; one centered on each thigh, five cm above the superior pole of the patella; and one just inferior to the sternal notch. A digital camera (Panasonic DMC-ZS7) was placed, on a tripod, three meters in front of the patient, with the camera position at the level of the patient's proximal thigh.

Instruction

The motion was described and demonstrated to the patient. The examiner stood to the side of the patient, so the patient could observe the depth of the squat, but would not be able to observe the frontal plane motion of the examiner's lower extremity. The patient was instructed to place their hands across their chest and to flex the knee on their non-weightbearing limb so the hip was maintained in 0 degrees of extension. The patient was instructed to squat as far as they could. The patient was then allowed to perform the motion up to three times to familiarize themselves with the motion and for the examiner to assess the depth of their squat. A goniometer was used to determine if the patient was able to achieve 60 degrees of knee flexion. If the participant did not reach at least 60 degrees of knee flexion, as judged visually by the examiner, they were instructed to increase the depth of the squat. Once the patient indicated they were comfortable with the movement, three trials were collected. If the patient lost their balance during a recorded trial, another trial was collected. Loss of balance was defined as the following: 1) placing the untested limb on the ground before completing the movement; 2) demonstrating extraneous movement of the upper extremities; 3) trunk lean that resulted in excessive motion of the untested limb; or 4) movement of the stance limb by sliding, hopping, or twisting the stance foot.

Data Storage

Immediately after the session, the videos were downloaded from the digital camera to a secure server, then deleted from the camera.

Measurement

A research assistant completed the measurement using a free, commercially available software (Kinovea). The methods for measurement are outlined in the Figure below. Initial position was defined as the frame just before the person begins to flex the knee of their weightbearing limb. The final depth of the squat was defined as frame just before the person begins to extend their knee after they have reached the maximum depth of their squat.

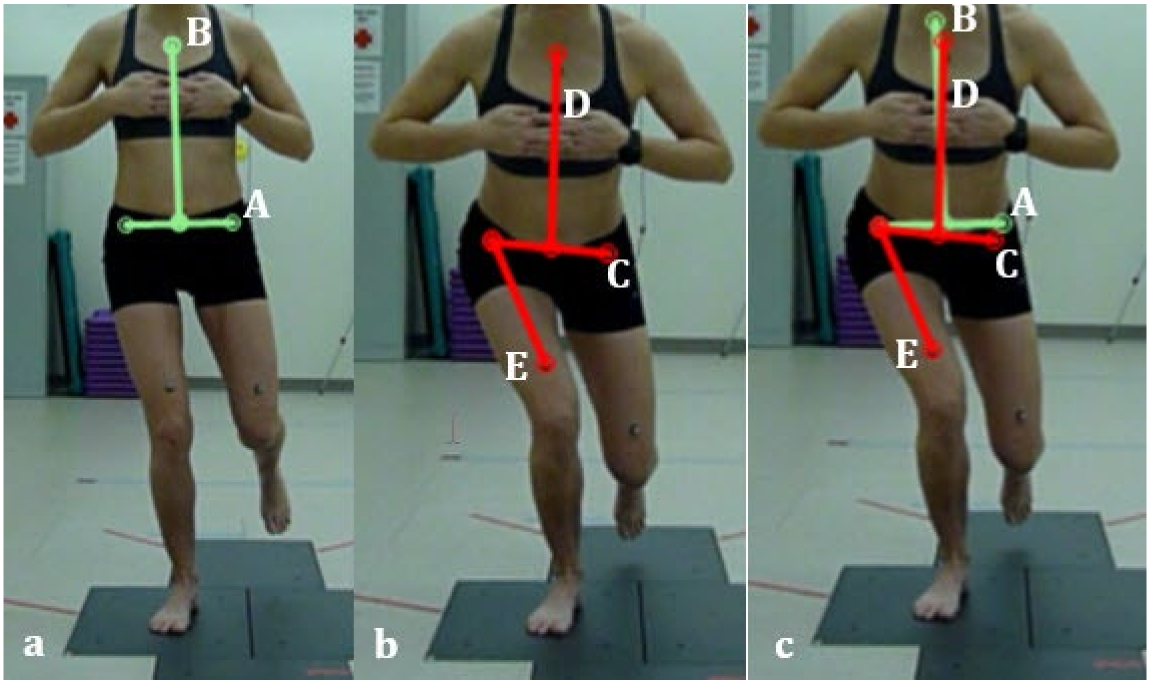

Figure. (A) Initial position. Line A is drawn between the anterior superior iliac spine (ASIS) markers and is the initial position of the pelvis. Line B is drawn from the sternal marker to the midpoint of line A and is the initial position of the trunk. (B) Final depth of squat. Line C is drawn between the ASIS markers and is the final position of the pelvis. Line D is drawn from the sternal marker to the midpoint of line C and is the final position of the trunk. Line E is drawn from the ASIS marker to the femur marker on the weight-bearing leg and is the final position of the femur. (C) Angle measurement. Hip adduction angle is defined by lines C and E. Positive values indicate hip adduction; pelvic excursion is defined by lines A and C. Negative values indicate a pelvic tilt in which the non-weight- bearing side is lowering; trunk excursion is defined by lines B and D. Positive values indicate the trunk is leaning towards the weightbearing limb. Reprinted from [Blinded for review].
